# Surgical implementation gap: an interrupted time series analysis with interviews examining the impact of surgical trials on surgical practice in England

**DOI:** 10.1136/bmjqs-2022-015077

**Published:** 2022-10-21

**Authors:** Kelly Ann Schmidtke, Felicity Evison, Amy Grove, Laura Kudrna, Olga Tucker, Andy Metcalfe, Andrew W Bradbury, Aneel Bhangu, Richard Lilford

**Affiliations:** 1 Arts and Sciences, University of Health Sciences and Pharmacy in St Louis, Saint Louis, Missouri, USA; 2 Warwick Medical School, University of Warwick, Coventry, UK; 3 Department of Informatics, University Hospitals Birmingham NHS Foundation Trust, Birmingham, UK; 4 Division of Health Sciences, Warwick Medical School, University of Warwick, Coventry, UK; 5 Institute of Applied Health Research, University of Birmingham, Birmingham, UK; 6 University Hospitals Birmingham NHS Foundation Trust, Birmingham, Birmingham, UK; 7 Institute of Cardiovascular Sciences, University of Birmingham, Birmingham, UK

**Keywords:** Surgery, Implementation science, Evidence-based medicine, Performance measures

## Abstract

**Objectives:**

Landmark studies published near the turn of the 21st century found an implementation gap concerning the effect of evidenced-based findings on clinical practice. The current study examines the uptake of six trials that produced actionable findings to describe the effects of evidence on practice and the reasons for those effects.

**Design:**

A sequential, explanatory mixed methods study was conducted. First, a quantitative study assessed whether actionable findings from large, publicly funded elective surgical trials influenced practice. Subsequently, qualitative interviews were conducted to explain the quantitative findings.

**Setting:**

Changes in NHS-funded practice were tracked across hospitals in England. Interviews were conducted online.

**Data and participants:**

The six surgical trials were funded and published by England’s National Institute for Health Research’s Health Technology Assessment programme between 2006 and 2015. Quantitative time series analyses used data about the frequencies or proportions of relevant surgical procedures conducted in England between 2001 and 2020. Subsequently, qualitative interviews were conducted with 25 participants including study authors, surgeons and other healthcare staff in the supply chain. Transcripts were coded to identify major temporal events and Consolidated Framework for Implementation Research (CFIR) domains/constructs that could influence implementation. Findings were synthesised by clinical area.

**Results:**

The quantitative analyses reveal that practice changed in accordance with findings for three trials. In one trial (percutaneous vs nasogastric tube feed after stroke), the change took a decade to occur. In another (patella resurfacing), change anticipated the trial findings. In the third (abdominal aortic aneurysm repair), changes tracked the evolving evidence base. In the remaining trials (two about varicose veins and one about gastric reflux), practice did not change in line with findings. For varicose veins, the results were superseded by a further trial. For gastric reflux, surgical referrals declined as medical treatment increased. The exploratory qualitative analysis informed by CFIR found that evidence from sources apart from the trial in question was mentioned as a reason for non-adoption in the three trials where evidence did not affect practice and in the trial where uptake was delayed. There were no other consistent patterns in the qualitative data.

**Conclusion:**

While practice does not always change in the direction indicated by clinical trials, our results suggest that individuals, official committees and professional societies do assimilate trial evidence. Decision-makers seem to respond to the totality of evidence such that there are often plausible reasons for not adopting the evidence of any one trial in isolation.

**WHAT IS ALREADY KNOWN ON THIS TOPIC**

- Publicly funded randomised controlled trials comparing the efficacy of two or more treatments can produce directive results for clinical practice that stand to improve health.
- However, previous studies have suggested that implementation of results is slow and may not take place at all.

**WHAT THIS STUDY ADDS**

- Systematic failure to respond to evidence is no longer apparent, at least in the domain of elective surgery in a high-income country (England).
- As trial evidence accumulates, there is an increasing chance that the findings of one trial will be superseded by findings from other contemporaneous studies.
- Recommended (or evidence-based) changes in practice may be delayed while policy makers wait for additional evidence and a gradual change in structures and norms.

**HOW THIS STUDY MIGHT AFFECT RESEARCH, PRACTICE OR POLICY**

- Research commissioners and trial authors could be jointly responsible for ensuring that trial findings are accessible to inform implementation.
- Evidence-based practice should be build around assimilating the totality of evidence rather than a simple ‘question and answer’ paradigm.

## Introduction

Organisations that fund clinical research often prioritise pragmatic randomised controlled trials (RCTs) that can generate robust evidence to improve healthcare.^1^ Such agencies include, but are not limited to, the National Natural Science Foundation in China, the National Institute for Health (NIH)–Healthcare Systems Research Collaboratory programme in the USA and the National Institute for Health and Care Research (NIHR)–Health Technology Assessment (HTA) programme in the UK. However, even results that appear to yield clear benefits for one treatment over another may not change practice.^2^ Based on trials published in the late 20th century, widely reported studies find that only half of actionable trial findings are implemented in practice and that it may take 17 years for robust evidence-based practices to become routine.^3–5^ These studies contributed to the development of ‘implementation science’, which seeks to understand the circumstances that facilitate the implementation of evidence-based findings.

Two large studies have examined the impact of the UK’s HTA programme between 1993 and 2013.^6 7^ Both concluded that the programme could positively impact patient outcomes through changes in perceived policy and practice. To improve impact, the later study recommended targeted funding for dissemination and increased transparency around patient involvement. The study also called on researchers to consider implementation from the outset.^7^ However, what these studies lack is a contribution to our understanding of what factors and challenges impact successful implementation of the intervention itself or how these factors can be addressed.

The reasons describing why implementation does, or does not occur, can be organised using an internationally regarded framework called the Consolidated Framework for Implementation Research (CFIR).^8^ The CFIR includes 41 empirically supported constructs organised across five domains, including characteristics of the innovation in question (eg, two constructs include evidence strength and cost), individuals involved (eg, knowledge and self-efficacy), inner setting (eg, culture and available resources), outer setting (eg, external policies/incentives and patient needs) and the process encouraging uptake (eg, planning and patient engagement). In this study, we aim to use the CFIR to theoretically inform our data collection and help to organise our interpretation of the qualitative results.

In a previous study, we used the Hospital Episode Statistics (HES) database to track the performance of emergency surgical procedures assessed in three trials funded by the NIHR HTA programme.^9^ In two trials, Distal Radius Acute Fracture Fixation Trial (DRAFFT)^10^ and Proximal Fracture of the Humerus: Evaluation by Randomisation (ProFHER),^11^ we found that use of the superior option increased in practice. But surprisingly, that increase started before study findings were published. In a third trial, the Ankle Injury Management (AIM),^12^ we found that the frequency of the intervention remained high despite the trial findings favouring the less invasive comparator. Overall, we found that publication of trial results was not followed by a change in practice. Similar to the previous HTA evaluations,^6 7^ we failed to conceptualise why or how practice had or had not changed.

In the current study, we aim to extend our previous work to provide this missing evidence. We adopt a mixed methods design with an expanded number of elective surgical trials of mixed surgical populations. Our first aim is to describe quantitatively whether practice changed after the publication of each trial. Our second aim is to qualitatively explore why practice had or had not changed.

## Methods

### Study design

A sequential, explanatory, mixed methods study design was used in which the quantitative phase was followed by the qualitative phase to contextualise the quantitative findings.^13^ We aimed to increase the number and type of trials considered in our analysis which can increase the depth, breadth and usefulness of our findings. The quantitative study was approved by the University Hospitals Birmingham NHS Foundation Trust. The mixed methods study was reviewed by the UK’s Health Research Authority which delegated responsibility for ethical approval to the University of Warwick. The study was preregistered on the Open Science Framework platform (osf.io/j6qdc). The methodological orientation underpinning the study was subtle realism, in which the research aims to represent the reality of clinical practice.

### Research team

The core research team was led by a professor with over 40 years of experience in medicine (RL), an assistant professor trained in mixed methods research and psychology (KAS), and a hospital statistician with experience using the HES database (FE). The team was further complimented by academic experts in implementation science (AG and LK) and clinicians specialising in the clinical areas examined (AM, OT, AWB, AB).

### Patient and public involvement

Before obtaining ethical approval, the study was discussed with four public contributors whose comments shaped our semistructured interview guide. After the transcripts were coded, four additional contributors reviewed the meaningfulness and trustworthiness of our interpretations.

### Trial selection

The trials were selected by reviewing the titles and abstracts of 655 studies published in the *Health Technology Assessment* journal between 2006 and 2015 (inclusive). We included surgical trials with actionable findings, that is, the trials with the greatest potential to influence practice.^14^

We defined ‘actionable’ findings as those in which the experimental treatment was found to be superior to the comparator(s), or not inferior to comparator(s) with known lower costs and side effects. We excluded trials that did not yield actionable findings. We also excluded pilot/feasibility studies.

We selected surgical trials because we can track the uptake of findings electronically through routine data (using the HES database). We defined ‘surgery’ as an invasive procedure with some cutting of tissues. Nine trials were initially identified, including three that were in our previous study (DRAFFT, ProFER and AIM)^9^ and six new trials (FOOD, EVAR, REFLUX, KAT, REACTIV and CLaSS). The trials selected were reviewed by three NIHR HTA administrators who did not identify any missed surgery trials. Each trial is described below. Further details are in online supplemental material 1.

10.1136/bmjqs-2022-015077.supp1 Supplementary data

- Stroke: The Feed Or Ordinary Diet (FOOD) trial compared the proportion of patients surviving without disability after being admitted to hospital with a stroke and experiencing either nasogastric (NG) tube feeding or percutaneous endoscopic gastrostomy tube feeding. NG tube feeding was identified as the superior treatment.^15^
- Gastro-oesophageal reflux disease: The Randomised Evaluation oF Laparoscopic sUrgery for refluX (REFLUX) trial compared reflux severity after laparoscopic fundoplication to continued medical management. Surgery was identified as the superior treatment.^16 17^
- Abdominal aortic aneurysm: The EndoVascular Aneurysm Repair (EVAR) 1 trial compared mortality for patients after experiencing endovascular or open repair. Their 30-day results favoured endovascular repair.^18^ EVAR 2 compared endovascular repair to no surgery for patients unfit for open surgery and its results were more nuanced. The current study focuses on EVAR 1.
- Knee replacement: The Knee Arthroplasty Trial (KAT) compared patient-reported outcomes for patients who experienced a total knee replacement with or without patella resurfacing.^19^ While the outcomes did not differ, the cost-effectiveness analysis supported resurfacing.
- Varicose veins 1: The Randomised and Economic Assessment of Conservative and Therapeutic Interventions for Varicose Veins (REACTIV)^20^ trial compared patient-reported outcomes after experiencing surgery over conservative treatments. Surgery was identified as the superior treatment.
- Varicose veins 2: The Comparison of LAser, Surgery and foam Sclerotherapy (CLaSS)^21^ trial compared patient-reported outcomes after experiencing endovenous laser ablation, surgery or sclerotherapy. Endovenous laser ablation was identified as the superior treatment.

### Quantitative data

#### Collection

Quantitative data were retrieved from the HES database.^22^ The HES database captures single records of NHS-funded activity to inform hospital remuneration and policy. HES records are given in ICD-10 (International Classification of Diseases 10th revision) diagnosis codes and OPCS-4 (Office of Population Censuses and Surveys Classification of Interventions and Procedures version 4) procedure codes. Patient details (eg, age) and administrative details (eg, emergency/elective admissions) are also captured. Hospital coders and surgeons provided advice to capture the procedures described in each HTA report. The HES database does not contain information about why patients are referred to hospital and coding activity can be affected by policy changes.

We planned to plot the treatments considered in each clinical area as comparable proportions of use in 3-month intervals, starting in 2001 and ending in the first quarter of 2020. However, for the FOOD trial, data could not be extracted for NG tube insertions; here the denominator was the first admission for all patients admitted with stroke who spent at least one night in hospital. For the REFLUX trial, data could not be captured about conservative medical management; here data were plotted using the number of treatments, and we did not restrict to the first surgical intervention per patient. For the REACTIV/CLaSS trials, the timeline starts in 2006 because this is when outpatient data about endovenous laser ablation became available on HES. Full details on data extraction are contained in online supplemental material 2.

#### Analysis

Quantitative tests involved fitting a linear model to the time series data, where the outcome variable was the respective indicator for the trial and the predictor variable was the period. To assess whether there was a break in the trend, we used a cumulative sum test of recursive residuals. Where a break was identified, the date of the break was located using a Wald test. Then, separate linear models were fitted before and after this date. The analyses were performed using STATA statistical software: Release V.15 SE (StataCorp LLC, Texas, USA), p values <0.05 were considered statistically significant. Trials for which the trend ultimately moved in the direction anticipated by trial findings were considered ‘implemented’ and trials for which the trend did not move in the anticipated direction were considered ‘not implemented’.

### Qualitative data

#### Collection

Graphs summarising the quantitative analyses were produced to discuss with interview participants. The graphs included lines indicating when trial recruitment ended and when the results were published in the *Health Technology Assessment* journal.

Snowball sampling methods were used to purposively recruit interview participants who would have knowledge of the trial and the procedures investigated. Clinical area specialists on our research team were provided with a template email to contact the participants on behalf of the project. Our recruitment started with up to two trial authors who would be aware of clinical practice, for example, chief investigators, but not statisticians. These participants were then asked to identify surgeons and other healthcare staff who could offer varying perspectives; each new participant could recommend further participants. The chief investigators for the REFLUX and EVAR trials were not available. For the REFLUX trial, none of the coauthors responded to recruitment emails, and for EVAR, an interview with an alternative author was delayed until December 2021. In both trials, our interviews started with surgeons recommended by our clinical area specialists.^23^

Participants were provided with an information sheet describing our study aims and indicated their informed consent before their interview started. Interviews were conducted from February 2021 to December 2021 by KAS (identifies as female) using Microsoft Teams according to a semistructured guide (online supplemental material 3) and typically lasted less than 30 min. The guide was pilot tested and revised with coauthor input. During the interviews, the relevant graph(s) were presented for discussion. Participants were invited to freely discuss what they believed influenced practice across the 20 years displayed on the graphs. Probing questions included in our interview guide were used flexibly to capture information according to the Consolidated Framework for Implementation Research’s (CFIR) theoretical domains: that is, one question for each domain.^8^ Transcripts of the audio recordings were created with identifiable information redacted. Videos of the interviews were retained to check for accuracy during analyses and thereafter deleted.

#### Analyses

Anonymised transcripts were uploaded to NVivo V.1.0 for coding. Initial coding was conducted by a single researcher (KAS) with emerging codes reviewed by KAS and AG. The coding approach was deductive and involved two types of codes. The first code type described temporal events that could influence implementation, for example, a new National Institute for Health and Care Excellence (NICE) guideline. Only events confirmed by reviews of documents or online searches were added to our graphs. The second type of code depicted each of the 41 CFIR constructs organised by domain according to the 2014 CFIR codebook, 8 24 with an opportunity to add inductive codes as and when identified during our analysis. While the interview probe questions broadly reflected the CFIR domains, the second type of more exploratory coding took place at the level of the CFIR constructs to enable a higher level conceptualisation of the raw data.

#### Within trial analysis

Next, to explore patterns within each trial, we examined the data across the CFIR domains. We searched the data for evidence of barriers or facilitators to implementation and examples of these barriers or facilitators which could provide illustrative quotes.^8^ The results are presented as narratives to illustrate the most illuminating information captured in the interviews. We present all coded data online supplemental tables.

#### Across trial analysis

Finally, we examined the data across trials. We explored patterns across constructs for all six trials (ie, the whole dataset). This stage of our analysis focused on the abstraction of the data to identify the overarching lessons for implementation of trials across our dataset. To enhance the transparency of this process, a summary table was created to identify constructs across trials that consistently represented barriers or facilitators for trials.

In this stage of analysis, we confirmed that no new themes arose from the data about implementation beyond those given by the CFIR.^25^ The final codes and our interpretations were cross-checked through conversations with the research team, public contributors and administrators from the NIHR Centre for Engagement and Dissemination. Online supplemental material 4 contains all extracted data.

## Results

**Figure 1:**
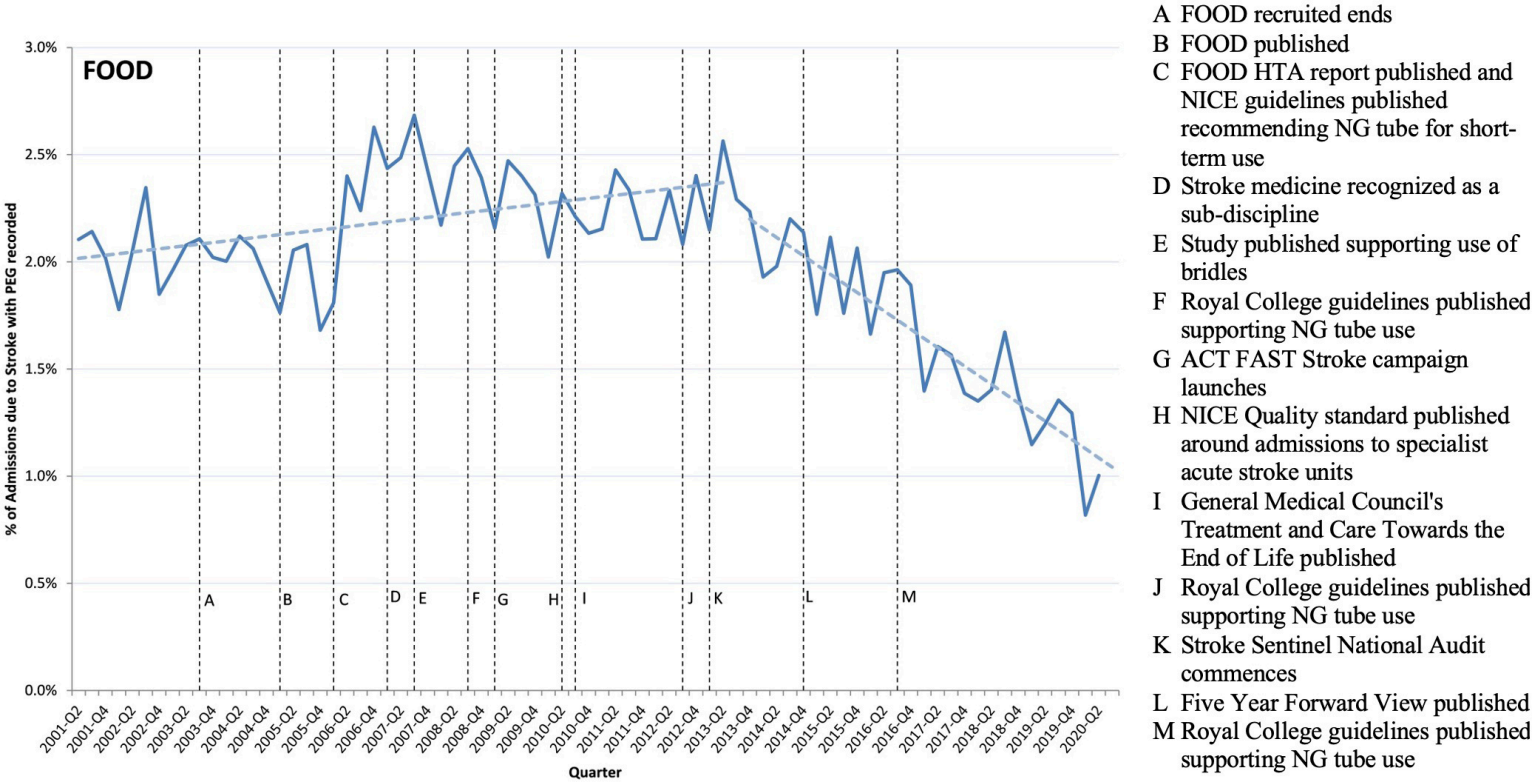
FOOD trial timeline. Note: This figure shows the percentage of admissions with primary diagnosis of stroke where the patient stayed overnight and had a PEG recorded during their stay, along with events potentially influencing implementation of evidence-based findings from the FOOD trial. FOOD, Feed Or Ordinary Diet; HTA, Health Technology Assessment; NG, nasogastric; PEG, percutaneous endoscopic gastrostomy.

**Figure 2:**
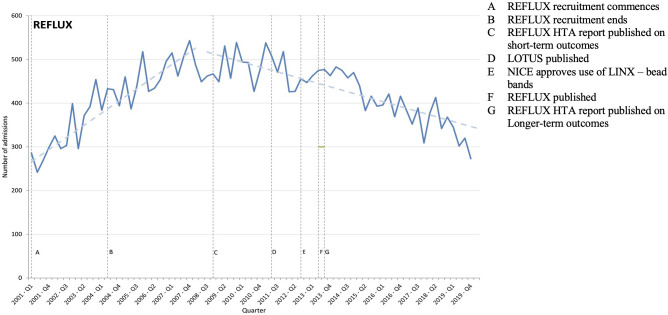
REFLUX trial timeline. *Note:* This figure shows the number of admissions with a primary diagnosis of gastro-oesophageal reflux disease where the patient underwent anti-reflux fundoplication surgery, along with events potentially influencing implementation of evidence-based findings from the REFLUX trials. HTA, Health Technology Assessment; NICE, National Institute for Health and Care Excellence; REFLUX, Randomised Evaluation oF Laparoscopic sUrgery for refluX.

**Figure 3:**
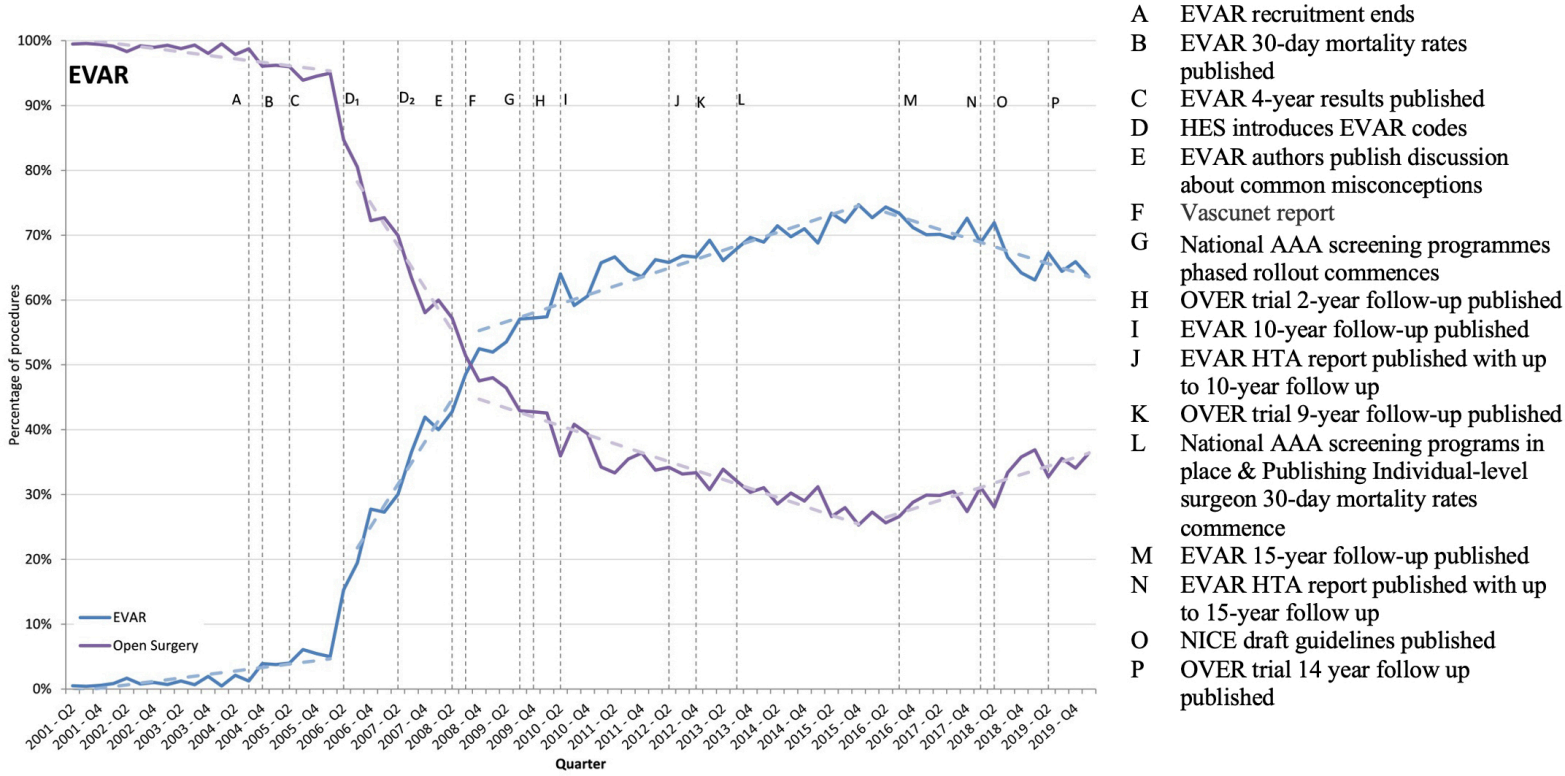
EVAR trial timeline. Note: This figure shows the percentage of EVAR and open surgeries performed for elective admissions with a primary diagnosis, along with events potentially influencing implementation of evidence-based findings from the EVAR trial. EVAR, EndoVascular Aneurysm Repair; HTA, Health Technology Assessment.

**Figure 4:**
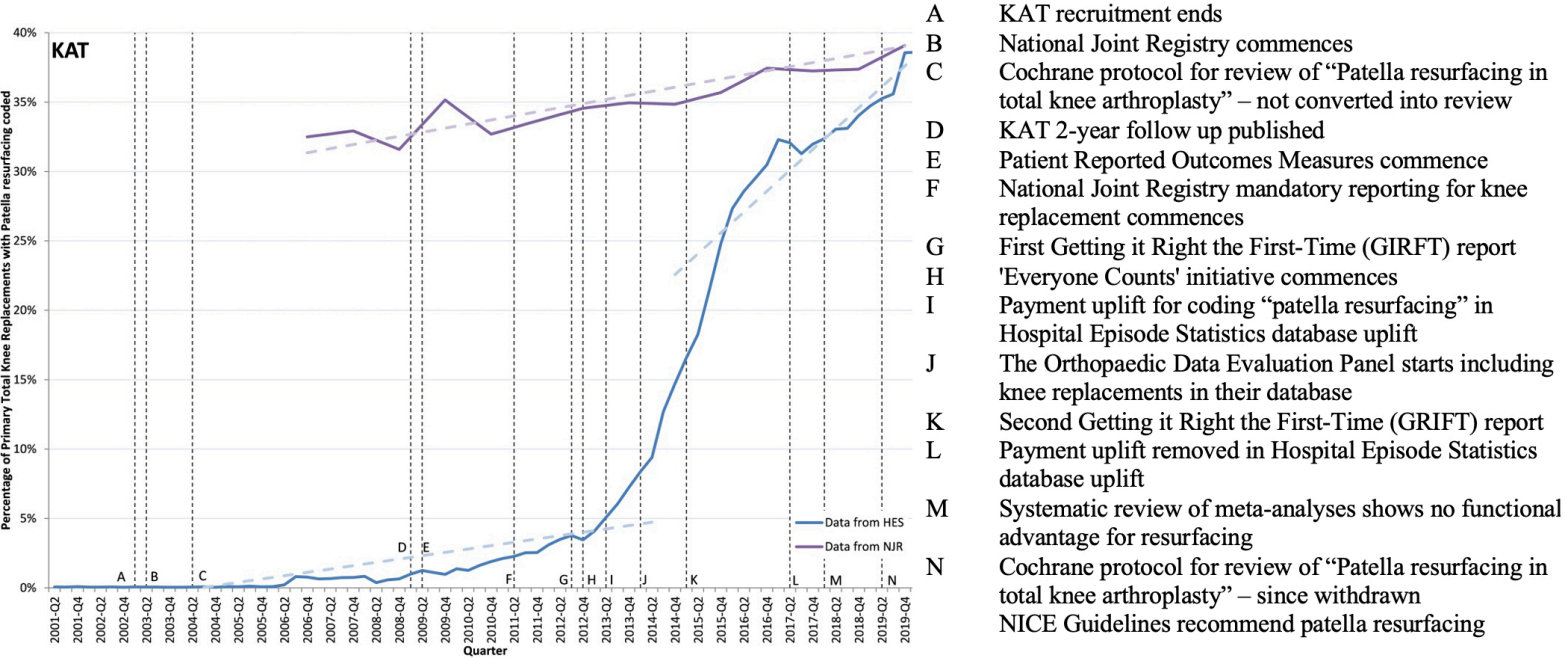
KAT trial timeline. Note: This figure shows the percentage of patients having total knee replacement, who also have a code for resurfacing the patella, along with events potentially influencing implementation of evidence-based findings from the KAT trials. The blue line shows data reported in the Hospital Episodes Statistic database and the purple line shows data reported in the National Joint Registry (NJR). Data from the NJR are only published annually, so the true quarterly line may not be as smooth as is shown. KAT, Knee Arthroplasty Trial; NICE, National Institute for Health and Care Excellence.

**Figure 5:**
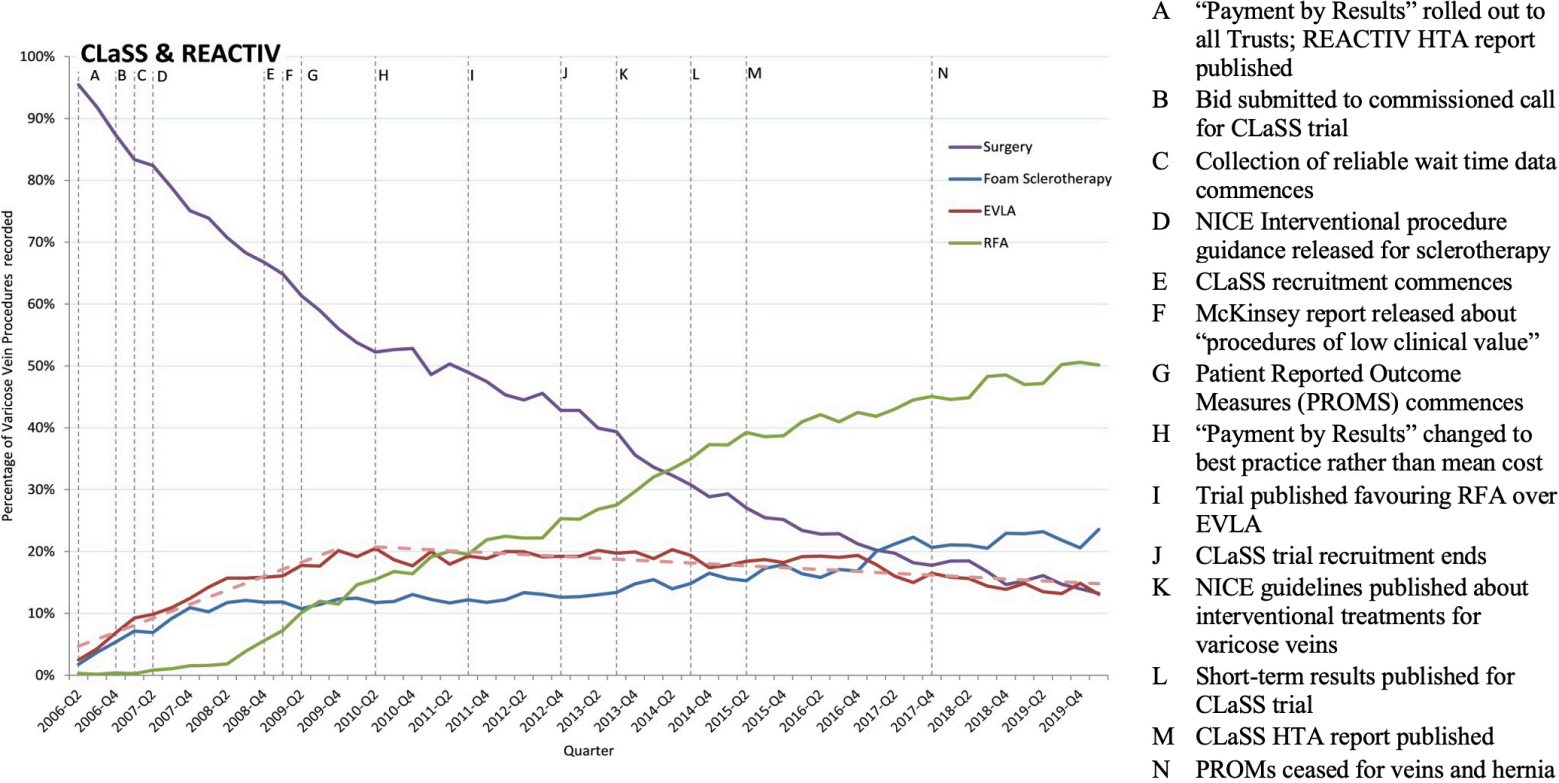
REACTIVE and CLaSS trials timelines. Note: This figure shows the percentage of varicose vein procedures undertaken in hospital (either inpatient or outpatient settings) by the type of procedure, along with events potentially influencing implementation of evidence-based findings from the REACTIV and CLaSS trials. CLaSS, Comparison of LAser, Surgery and foam Sclerotherapy; HTA, Health Technology Assessment; REACTIV, Randomised and Economic Assessment of Conservative and Therapeutic Interventions for Varicose Veins.

The quantitative results are presented in graphical time series (figures 1–5), where solid lines represent how often each treatment was used and dashed lines represent the estimated trends. This information is presented within the qualitative results as narratives to describe the major temporal events and the CFIR domains that influenced implementation by clinical area. Our exploratory findings are mapped across trials according to the CFIR domains which are presented in square brackets.

### Participant characteristics

The 25 interview participant characteristics are summarised in table 1. The job titles of other stakeholders were not predefined; this category included dietician, speech and language therapist, radiologist, gastroenterologist and general practitioner. Four general practitioners contributed insights across multiple trials, and one participant took part in an interview about EVAR (as a surgeon) and varicose veins (as an author); for this reason, the number of participants provided in the total column does not equal the total number of interviews. The participants had a median of 20 years (5–44 years) of work experience.

**Table 1:** Participant characteristics

Topic	Trial	Authors	Surgeons	Other	Total
Stroke	FOOD	1	1	2	4
Gastro-oesophageal reflux disease	REFLUX	0	1	6	7
Abdominal aortic aneurysm	EVAR	1	2	5	8
Knee replacement	KAT	1	2	5	8
Varicose veins	REACTIV/CLaSS	3	3	5	11

CLaSS, Comparison of LAser, Surgery and foam Sclerotherapy; EVAR, EndoVascular Aneurysm Repair; FOOD, Feed Or Ordinary Diet; KAT, Knee Arthroplasty Trial; REACTIV, Randomised and Economic Assessment of Conservative and Therapeutic Interventions for Varicose Veins.

### Within trial results

#### Stroke: FOOD trial

The FOOD trial, published in 2005, found that percutaneous endoscopic gastrostomy (PEG) tube feeding was no more effective and caused more negative side effects than NG tube feeding. PEG tube use started to decrease only in 2013 (figure 1). A break was estimated in 2013 (p<0.001, Supremum Wald statistic=127.59), followed by a significant downward trend (beta coefficient: −0.04 (95% CI −0.05 to –0.03) p<0.001).

Before the FOOD trial, PEG tube use was supported by evidence from a trial published in 1996 with just 30 participants.^26^ The FOOD trial produced higher quality evidence supporting the use of NG tubes with 321 participants (CFIR Intervention domain). Our interviews shed light on the delay of at least 8 years between findings and practice. Early on, staff were reluctant to use NG tubes because patients tended to pull them out (CFIR Inner Setting domain). A study supporting the use of some restraints was published in 2007 that increased staff confidence.^27^

There was a lot of nursing literature, which was very much pushing against any form of restraint [which was] seen as unethical. And I think, hopefully, we now have a more balanced view, that you've got to take a holistic view of what you’re trying to achieve. (Author)

This was followed in 2008 by the National Stroke Guidelines which adopted an earlier recommendation to switch to NG tubes from the NICE (Outer setting).^28^ Additionally, the General Medical Council’s (GMC) 2010 guide increased staff confidence (Individual) in decisions not to tube feed patients whose quality of life would be low if they survived.^29^

We are much more explicit now with families about the value of surviving with severe disability and ensuring that we've established the patient’s wishes to a much greater extent than we did in the past. So, it’s interesting, isn’t it? Because that wasn’t one of the original hypotheses that the FOOD trial was testing. But it’s proved to be part of a landscape which has prompted us to think in more detail about what it means to survive with a severe disability. (Surgeon)

Increasing awareness of the importance of quality care in stroke was also aided by Public Health England’s Act FAST (Face, Arm, Speech, and Time) campaign in 2009,^30^ the NICE quality standard prompting admission to specialty stroke wards in 2010,^31^ and the start of the Stroke Sentinel National Audit in 2013 (Outer setting).^32^ These guidelines support collaborations across a diverse array of staff, including but not limited to dieticians and speech and language therapists (Individual).

In conclusion, the FOOD trial was the first stage in a series of events that unfolded over many years that did eventually result in a change in practice. In terms of the CFIR framework, the FOOD trial provided the necessary preliminary evidence to motivate a change in practice that only occurred after changes in the outer setting: additional evidence and publication of national guidelines convinced practitioners that they could use the NG tubes safely and effectively.

#### Gastro-oesophageal reflux disease: REFLUX trial

Despite the REFLUX trial finding superior outcomes for laparoscopic surgery (fundoplication) compared with conventional medications, the use of surgery declined (figure 2). The cumulative sum test confirms a break in 2008 (p<0.001, Supremum Wald statistic=222.36), after which there is a downward trend (beta coefficient: −3.78 (95% CI −4.59 to –2.98), p<0.001).

The downward trend that started in 2008 continued despite the 2011 publication of the large LOTUS trial replicating the REFLUX trial findings (Intervention).^33^ One potential explanation for the continuing decrease could be that an alternative surgery system, called LINX, was approved by NICE in 2012 (Outer setting and Intervention).^34^ However, very few LINX surgeries have been recorded on HES. Across participant categories, interviews quickly converged on an explanation for why surgical interventions had not increased: reduced general practitioner referrals (Outer setting).

We’re sort of dependent on our referral pathways which often will come either through gastroenterology or direct from GPs [general practitioners]. And then once they are referred to us, normally that’s people that are already a bit or at least partially aware of what anti-reflux surgery involves. And a lot of the patients we see, if they’re diagnosed with pathological reflux, we’ll proceed with surgery in general. (Surgeon)

General practitioners believed that patient symptoms could be managed through medication-based treatments and lifestyle modifications (Individual). This was supported by NICE guidelines that recommend surgery only for patients who do not wish to continue acid suppression therapy (Outer setting). While the REFLUX trial’s longer term cost-effectiveness analyses support surgery, shorter term barriers appeared to preclude increases. For instance, general practitioners believed that the system lacked the capacity to support a large increase in referrals (Individual and Inner setting), and commissioning bodies were not convinced by the formal cost-effectiveness model (Outer setting).

You’ve missed out on probably the most influential layer and that’s the CCG [clinical commissioning group] layer. Bottom line is if the medical conservative therapy, omeprazole, lansoprazole, whatever, it’s relatively cheap as chips, and I wouldn’t say we quite dish it out like smarties but it’s a nice easy fix. (General practitioner)

In conclusion, clinical practice has not changed in the direction anticipated by the REFLUX trial. While evidence from two large trials suggests that surgery is effective, the use of low-cost medication of established effectiveness dominates surgical interventions for gastro-oesophageal reflux disease.

#### Abdominal aortic aneurysms: EVAR trial

In line with the EVAR trial’s initial 30-day trial, the use of endovascular repair increased rapidly from 2004 to about 2012 (figure 3). For endovascular surgery, a break is identified in 2006 (p<0.001, Supremum Wald statistic=616.90), after which there is an upward trend (beta coefficient: 3.27 (95% CI 2.31 to 4.24) p<0.001). Another break occurs in 2008 (p<0.001, Supremum Wald statistic=165.08), followed by a flatter increasing trend (beta coefficient: 0.69 (95% CI 0.59 to 0.79) p<0.001). A final change occurs in 2016 (p<0.001, Supremum Wald statistic=165.08), followed by a decreasing trend (beta coefficient: −0.66 (95% CI −0.88 to –0.44), p<0.001).

All changes in practice closely track the evolving evidence (Intervention). The initial increase in 2004 tracks the 30-day findings favouring endovascular surgery, first published in *The Lancet* that year.^35^ The second change tracks publication of the 8-year follow-up, which was published in 2010 in the *New England Journal of Medicine* and found no differences in mortality between treatments.^36^ The final change tracks publication of the 15-year follow-up, which was published in 2016 in *The Lancet* and revealed a mortality cross-over, such that the all-cause mortality rate was higher for endovascular than for open surgery after 8 years.^37^ ^38^

Interview participants noted that the initial results favouring endovascular repair were appealing to clinicians, patients and hospital administrators (Outer setting, Inner settings, Individual, Process). Not only did endovascular repair initially result in lower mortality rates but also reduced pain, and quicker hospital discharge.

The surgeon’s main preoccupation is reducing the absolute risk in the perioperative period … it is a very painful event both from the family and from the surgeons’ [point of view]. (Surgeon)

Patients get quicker better, they like it [EVAR]. Hospital beds are becoming fewer in number, and critical care beds are becoming fewer in number and difficult to get. These are quicker operations from which, compared to open surgery, you can send them quicker. (Surgeon)

In 2008, a Vascunet report stated that the UK had the highest 30-day mortality rates for elective open repair in Europe (Outer setting).^39^ In response, the National Health Service’s annual screening programme started a phased rollout (Process), during which increases in EVAR were facilitated by training programmes to enhance individual surgeon capabilities (Individual) and hospital capacity to manage increased caseloads (Inner setting). Efforts were also put into improving the design of stents.

I’ve gone to many, many, many vascular surgical meetings, and it was always about the EVAR and always about how you could improve EVAR, and I never once heard anyone talk about open surgery and how I’ve learnt to do something differently that improves my outcomes. And it’s almost like you were a dinosaur if you were talking about open surgery rather than the latest gizmo, so I think there’s a huge amount of psychology and finance that is driven these manufacturers want to sell. (Radiologist)

Large-scale meta-analyses support the mortality cross-over^40^ found in EVAR’s 15-year follow-up. In 2016, the use of endovascular repair started to decline. In 2018, NICE published draft recommendations that elective endovascular repair should not be offered to patients, largely informed by their cost-effective analyses (Intervention and Outer setting).^41^ Interviewees questioned whether the proposed guidelines meet patient needs and whether they were feasible to implement (Inner setting and Individual). In March 2020, NICE’s revised guidelines were published emphasising a need for shared decision-making (Outer setting and Process).^42 43^

In summary, changes in practice tracked the actional findings as they matured over lengthening follow-up periods. We found use of EVAR increased in line with short-term benefits before declining. The latest findings show a trade-off between short-term and long-term mortality outcomes. This nuance is reflected in the current NICE guidelines.

#### Total knee replacement: KAT trial

In line with the KAT trial’s finding, HES data show patella resurfacing started increasing before publication of the trial, see the blue line in figure 4.^44^ A cumulative sum test confirms a change in practice during 2015 in the third quarter (p<0.001, Supremum Wald statistic=532.0298), followed by a steep upward shift (beta coefficient=0.008; 95% CI 0.005 to 0.015), p<0.001).

Interview participants expressed concerns about the validity of the HES data (Individual and Inner setting). A coding expert explained that the payment structure was altered in 2013/2014, such that knee replacements coded with resurfacing received a payment uplift, which was removed in 2017/2018. Multiple participants suggested that we consider data from the National Joint Registry (NJR) for which reporting this procedure became mandatory in 2011.^45^ In figure 4, the purple line displays data from the NJR and shows a more gradual increase from 33% in 2007 to 39% in 2019.^46^

The increasing trend in resurfacing is supported by NICE’s 2020 guidance (Outer setting).^47^ The participants perceived the recommendation as largely driven by cost-effectiveness evidence (Intervention).

The evidence is really around the cost. The recommendation stems from the cost-effective analysis and the cost of secondary surgery. So, I think surgeons put different weight on that information than they do on satisfaction, functional outcomes, and other metrics. (Surgeon)

Participants noted geographical variations in practice, where resurfacing never occurs in some countries and in other countries is the norm; yet, patient outcomes do not differ.^48^ Additionally, there are variations in outcomes across implant brands and types.^49^ In the absence of reliable patient benefits (Outer setting), participants interpreted the move towards resurfacing as being defensive in preventing a temptation to resurface later, and as being largely guided by practitioner training and habits (Inner setting and Individual). A participant noted that the 20-year follow-up is in progress, which could generate new evidence.

It may be there are more problems with the patella resurfacing in the longer term. And if there is a problem with the resurfacing, they tend to be catastrophic, whereas just a late resurfacing is not catastrophic. So, I think there’s still a lot more to go with this trial. (Author)

In summary, we found that practice is increasing in line with KAT trial evidence and that current NICE guidelines support these practice changes.

#### Varicose veins: REACTIV and CLaSS trials

Changes in practice have not occurred in the direction anticipated by the REACTIV and CLaSS trials. Three changes in practice can be observed in figure 5. First, the use of traditional surgery has decreased from approximately 95% to 10%. Second, in 2010 the use of endovenous laser ablation increased (p<0.001, Supremum Wald statistic=387.05), but this increase started before the study results were published. Third, for radiofrequency laser ablation, there was a break in 2013 (p<0.001, Supremum Wald statistic=80.45), after which its use increased, and it becomes the dominant procedure.

Interview participants converged on common explanations for the decrease in traditional surgery having to do with decommissioning in the early 2000s bolstered by the McKinsey report in 2009 (Outer setting).^50^

There was a list of low-priority treatments that you ought to look outright and find somewhere, which would produce, oh, there was a lot of argy-bargy about it… and varicose veins were on it. And that also, you see, will have been influential. (Author)

Additionally, as the NICE approved less invasive surgical procedures, traditional varicose vein surgery became less attractive (Outer setting and Intervention). The NICE approved radiofrequency laser ablation in 2004,^51^ endovenous laser ablation in 2005^52^ and ultrasound-guided sclerotherapy therapy in 2007.^53^

The same mechanism of action underlies radiofrequency laser ablation and endovenous laser ablation, that is, both are ‘endothermal’ treatments. Participants expressed that the use of either procedure would be largely influenced by what equipment organisations made available (Inner setting).

Radiofrequency ablation got quite heavily sold by the people who made the equipment … the big teaching hospitals in vascular units have tended to adopt the endovenous laser, the laser therapies, whereas district general hospitals have been more inclined to take radiofrequency ablation. And part of that is about equipment. (Author)

The use of radiofrequency laser ablation became dominant over endovenous laser ablation in 2011, coinciding with the publication of a Danish trial finding superior outcomes for radiofrequency laser ablation (Intervention).^54^ Finally, NICE’s 2013 treatment guidelines recommend that patients are first offered endothermal ablation, and if unsuitable then ultrasound-guided foam sclerotherapy, and if unsuitable then traditional surgery (Outer setting).^55^

In conclusion, treatments applied for varicose veins have not changed in the direction anticipated by the REACTIV trial or the CLaSS trials. Instead, changes were more greatly influenced by commissioning constraints, the availability of equipment and evidence produced by a Danish trial which favoured an alternative procedure not included in REACTIV and CLaSS trials.

### Across trial results

Many of the CFIR constructs were identified as both barriers and facilitators in each trial (detailed in table 2 for completeness). In the three implemented trials, a greater number of constructs were identified as facilitators (n=44) than barriers (n=34). For non-implemented trials, a greater number of constructs were identified as barriers (n=41) than facilitators (n=28).

**Table 2:** Across trial results: barriers and facilitators to implementation across the six HTA trials by CFIR domains and constructs

	Implementation did occur						Implementation did not occur					
	FOOD		EVAR		KAT		REFLUX		REACTIV		CLaSS	
CFIR domains and constructs	Barrier	Facilitator	Barrier	Facilitator	Barrier	Facilitator	Barrier	Facilitator	Barrier	Facilitator	Barrier	Facilitator
1. Innovation characteristics												
1.A. Innovation source												
1.B. Evidence strength		√	X	√	X	√	X	√	X	√	X	√
1.C. Relative advantage	X	√	X	√	X		X		X			√
1.D. Adaptability												
1.E. Trialability												
1.F. Complexity	X	√	X		X	√		√	X		X	√
1.G. Design quality and packaging												
1.H. Cost			X	√		√	X		X		X	
2. Outer setting												
2.A. Needs and resources of those served	X	√	X	√			X		X		X	√
2.B. Cosmopolitanism												
2.C. Peer pressure					X	√					X	√
2.D. External policies and incentives	X	√	X	√		√	X		X	√	X	√
3. Inner setting												
3.A. Structural characteristics		√	X	√		√	X					
3.B. Networks and communications		√				√	X	√	X	√	X	√
3.C. Culture		√	X	√	X	√			X		X	
3.D. Implementation climate			X									
3.D1. Tension for change			X									
3.D2. Compatibility	X		X	√		√	X					
3.D3. Relative priority		√					X					
3.D4. Organisational incentives and rewards			X									
3.D5. Goals and feedback												
3.D6. Learning climate												
3.E. Readiness of implementation										√		√
3.E1. Leadership engagement												
3.E2. Available resources	X	√	X	√		√	X		X		X	√
3.E3. Access to knowledge and information		√			X	√	X					
4.Characteristics of individual												
4.A. Knowledge and beliefs		√	X	√	X	√	X	√	X		X	√
4.B. Self-efficacy		√	X	√		√	X	√	X	√	X	√
4.C. Individual stage of change					X	√						
4.D. Individual identification with organisation		√										
4.E. Other personal attributes												
5. Process												
5.A. Planning						√						
5.B. Engaging											X	√
5.B1. Opinion leaders			X	√						√	X	√
5.B2. Appointed internal implementation leaders												√
5.B3. Champions	x											
5.B4. External change agents			X			√					X	
5.B5. Key stakeholders												
5.B6. Innovation participants			X	√	X	√	X	√	X	√	X	√
5.C. Executing												
5.D. Reflecting and evaluating												

Evidence from sources apart from the trial in question was mentioned as a reason for non-adoption in the three trials where evidence did not affect practice and in the trial where uptake was delayed. Alternative sources of information justified non-implementation of the results of the two varicose vein trials and the REFLUX trial. Alternative information regarding risks of tube removal delayed adoption of the FOOD trial. We discerned no further clear patterns to describe implementation versus non-implementation of trial findings. For example, while the ‘Cost’ construct was a consistent barrier for all three non-implemented trials, ‘Cost’ was also discussed as a barrier for an implemented trial. While constructs related to the inner setting (eg, ‘Structure’, ‘Culture’ and ‘Available Resources’) were identified as facilitators in the three implemented trial, these constructs also acted as barriers in the some implemented trials or were not consistently identified as barriers in the non-implemented trials.

## Discussion

Our mixed method study illustrates that many factors influence the implementation of evidence-based findings. All six trials included in our study produced clear conclusions, and all were rigorously conducted and adequately powered to confirm their original hypotheses. The expectation of the funder at the time the trial was funded was that practice should change where a hypothesis was confirmed. We found that clinical practice moved in the direction anticipated in three trials only (50% were implemented and 50% were not implemented). Therefore, our study supports the previous literature.^3–5^ However, our study adds an understanding of why this happens and reveals a more nuanced evolution of implementation over the previous two decades.

Consider first the three trials where practice did follow evidence. In the FOOD trial, it was new evidence regarding the advantages of NG tube feeding and accumulating endorsements by respected organisations in the outer setting, such as NICE, that produced a gradual shift in stroke practice. In the other two trials, KAT and EVAR, trial evidence was also interpreted in context of other evidence. Such evidence must have influenced adoption of KAT trial findings even before the trial findings became available. Then when the findings were published, we find that evidence outside the trial tempered wholesale adoption of evidence from the trial itself.^56^ In EVAR, the evidence evolved, and this was reflected in practice. First, when the initial positive findings were published NICE ruled in favour of EVAR, and then funds were quickly allocated to the inner setting to purchase equipment and to train practitioners. Later, when the long-term results showed increased complications from EVAR, NICE first recommended against EVAR but then took a softened line to accommodate trade-offs between short-term and long-term outcomes that may turn on patient preferences.^57^

Next, we consider the three trials where practice did not move in the anticipated directions. In all trials, the relative merits of the intervention decreased as alternative evidence mounted. With regard to REFLUX, NICE supported surgery only if patients do not improve with medication treatments offered by general practitioners in the context of evidence on the effectiveness of such drug therapy.^58^ Implementation of the finding for varicose vein trials (REACTIV and CLaSS) superseded by evidence favouring a third treatment: radiofrequency ablation. NICE currently supports varicose vein surgery as a third-line treatment, after radiofrequency ablation and sclerotherapy, which demonstrates the ability of policy organisations to synthesise expanding pools of evidence.

### Strengths and limitations

A strength of our study stems from its mixed methods design. Our previous study tracked the implementation of three emergency orthopaedic trial findings using quantitative methods only.^9^ We extend these findings by examining six new trials. Our explanatory mixed method approach allowed us to consult subject experts on the topics of interest and helped to expand on the limited conclusion we can draw from quantitative HES data alone. For example, the interviewees highlighted potential inaccuracies in the KAT trial and helped us access alternative data to cross-check our findings. For the REFLUX trial, our test relied on the frequency of procedures rather than the proportion, as the HES database does not record the reasons these participants are referred. If medications are managing severe symptoms well, then this appears appropriate. However, if patients with severe symptoms are unduly suffering by not being offered a cost-effective surgery, then evidence alone may not change practice where other factors do not support its use.

A limitation of our study relates to the scope and size. The procedures identified in our prioritisation process were all elective surgeries and findings may be different in other areas, such as emergency surgery. Even within the domain of surgery, we have only six trials in our series, and cannot make any quantitative generalisations. Within each trial, we conducted a small number of interviews. Although theoretical saturation was judged to have been reached, it may be that a different pool of interviews could produce new themes. As a consequence of our snowball sampling, many healthcare professionals would be known by the study authors and clinical/research community. Despite this, we expect readers will find these six trials illustrative of reasons why results from pragmatic RCTs may or may not be adopted in practice.

We analysed our qualitative findings according to the CFIR.^8^ Our interview questions were framed openly and allowed participants to explore the issues they felt were most important in explaining the quantitative results displayed as graphical findings. This flexible method of interviewing means that we may have overlooked some constructs. However, all the CFIR domains were highlighted in our results, although some (eg, Outer setting) were identified more than others (eg, Process) (see table 2). It is possible that some constructs were not identified that could have altered uptake of findings but did not. For example, lack of training or equipment would have limited uptake of EVAR or patella resurfacing, but this problem did not arise.

### Implications for research commissioners

Our findings suggest that clinical and managerial practice are responding to research evidence. However, it is the totality of evidence that influences uptake, not just the results of individual trials. Questions remain for the research commissioning process regarding how implementation should be considered before a trial is funded. For example, the varicose vein trials did not produce the anticipated change, but this was because another technology was preferable to those evaluated in the trials. It would be unrealistic to expect funding bodies to only support ‘winners’. We could argue that the NIHR HTA programme has made a valuable contribution to the question of varicose vein treatment, notwithstanding its failure to influence practice in the hypothesised direction.

Our study provides strong evidence that the whole system is sensitive to emerging evidence and that organisational structures are in place to assimilate accumulating evidence holistically. In line with the previous evaluations of the HTA programme,^9^ patient involvement in innovation or implementation was evident across our trials and the knowledge generated is disseminated to promote awareness of the trial results.

We found that decisions often turn on evidence external to any particular study, and it follows that the investigators in a particular study may not be the most appropriate vehicle for promoting the uptake of their findings. In our view, funders should not focus on ensuring applicants state how they will disseminate their findings but instead need to work in partnership with authors and be jointly responsible for ensuring that findings are accessible and properly considered in the UK and abroad promptly where actionable results emerge. Situating implementation scientists in this collaborative process could facilitate the translation of evidence-based findings. While we are aware of instances, such as CRASH 2 trial,^59^ where one trial has substantially influenced practice, our findings suggest that such a result is the exception rather than the rule. Evidence-based practice should be built around assimilating the totality of evidence rather than a simple ‘question and answer’ paradigm.

## Conclusion

Early in the 2000s, independent research teams converged on a common time lag for evidenced-based findings to influence clinical practice: 17 years.^4 5^ Nearly 20 years later, we have no such simple message. Where the evidence from a trial was not implemented this was not because that evidence was not considered. While practice does not always change in the direction indicated by clinical trials, our results suggest that individuals, official committees and professional societies do assimilate trial evidence. Research trial evidence was considered along with evidence from other trials and relevant non-trial evidence. Decision-makers seem to respond to the totality of evidence such that there are often plausible reasons for not adopting the evidence of any one trial in isolation.

## Data Availability

No data are available. Data for our quantitative methods are available via the Hospital Episode Statistics (HES) database, but not from the research team. Quotes extracted from our qualitative methods are available as supplemental materials. In accordance with our ethical approvals, complete transcripts cannot be shared.
