# Supplementary material for: Surgical implementation gap: an interrupted time series analysis with interviews examining the impact of surgical trials on surgical practice in England
Source: BMJ Qual Saf. 2022 Oct 21;32(6):341–56. doi: 10.1136/bmjqs-2022-015077 (PMC10314058; doi:10.1136/bmjqs-2022-015077)
Supplement: Supplementary data [file bmjqs-2022-015077supp001.pdf]

Supplemental Materials 1. Summary of Six HTA Randomized Controlled Trial findings

- Stroke: The Feed Or Ordinary Diet (FOOD) trial compared the proportion of patients surviving without disability after being admitted to hospital with a stroke and experiencing either nasogastric tube feeding or percutaneous endoscopic gastrostomy tube feeding. Nasogastric tube feeding was identified as the superior treatment.[1]
- Gastro-oesophageal reflux disease: The Randomised Evaluation oF Laparoscopic sUrgery for refluX (REFLUX) trial compared reflux severity after laparoscopic fundoplication to continued medical management. Surgery was identified as the superior treatment.[2,3]
- Abdominal Aortic Aneurysm: The EndoVascular Aneurysm Repair (EVAR) 1 trial compared mortality for patients after experiencing endovascular or open repair. Their 30-day results favoured endovascular repair.[4] EVAR 2 results compared endovascular repair to no surgery for patients unfit for open surgery and were more nuanced; thus, the current study focuses on EVAR 1.
- Knee Replacement: The Knee Arthroplasty Trial (KAT) compared patient-reported knee function and pain after a total knee replacement with or without patella resurfacing.[5] While the patient-reported outcomes did not differ, the cost-effectiveness analysis supported patella resurfacing.
- Varicose Veins 1: The Randomised and Economic Assessment of Conservative and Therapeutic Interventions for Varicose Veins (REACTIV)[6] trial compared patient-reported outcomes after experiencing surgery over conservative treatments. Surgery was identified as the superior treatment.
- Varicose Veins 2: The Comparison of LAser, Surgery and foam Sclerotherapy (CLaSS)[7] compared patient-reported outcomes after experiencing endovenous laser ablation, surgery, or sclerotherapy. Endovenous laser ablation was identified as the superior treatment.

Clinical Area	Trial acronym (year the trial was published in the *Health Technology Assessment* journal)	Relevant Comparison	Patient Inclusion criteria	Primary outcomes	Superior treatment
Stoke	FOOD (2006)	nasogastric vs percutaneous endoscopic gastrostomy feeding tubes	admitted to hospital with stroke	• mortality<br>• poor outcome at follow-up (Modified Rankin Scale of 4 or 5)	nasogastric feeding
Gastro-oesophageal reflux disease	REFLUX (2008)	laparoscopic surgery (fundoplication) vs continued medical management	reflux disease symptoms for 12+ months controlled by medication	• REFLUX questionnaire score<br>• NHS costs	laparoscopic surgery
	REFLUX (2013)	5-year follow-up	5-year follow-up	• REFLUX questionnaire	laparoscopic surgery
Abdominal aortic aneurysm (AAA)	EVAR 1 (2012)	endovascular repair vs open repair	fit for open repair, 60+ years old, and AAA measuring 5.5+ cm	• mortality (operative, aneurysm related and all cause)	endovascular repair
Knee replacement	KAT (2014)	resurfacing patella vs not resurfacing	experiencing primary knee replacement surgery	• Oxford Knee Score	resurface patella
Varicose Veins	REACTIV (2006)	surgical treatment (ligation, stripping, and phlebectomies) vs conservative treatment	having a leg varicose vein larger than 5mm with reflux	• clinical effectiveness measured with short-form 6D	traditional surgery
	CLaSS (2015)	endovenous laser ablation vs surgery vs sclerotherapy	having a leg varicose vein, CEAP grade 2 or above	• Aberdeen Varicose Vein Questionnaire<br>• health-related quality of life at 6 months<br>• cost-effectiveness	endovenous laser ablation

Supplemental Materials 2. Data extraction quantitative codes

Trial	Diagnosis codes	Procedure/Operation codes	Time period	Indicator	Additional details about our data extraction.
FOOD	Primary diagnosis of:<br>I61.x – Intracerebral haemorrhage<br>I63.x – Cerebral Infarction<br>I64.x – Stroke, not specified as haemorrhage or infarction<br><br>(exclude anyone who didn't stay overnight unless they died)	G44.5 - fibreoptic endoscopic percutaneous insertion of gastrostomy **OR**<br>(G34.2 – creation of temporary gastrostomy **AND**<br>G44.8 - other therapeutic fibreoptic endoscopic operations on upper gastrointestinal tract)	Q2 2001-Q1 2020	**Proportion**<br>Denominator First admission for a patient with first recorded ICD-10 diagnosis code matching those in previous column, and who stayed at least one night in hospital<br><br>Numerator: Admissions meeting the denominator criteria and the procedure codes outlined in previous column.	For the FOOD trial, data could only be extracted for the number of percutaneous endoscopic gastrostomy tube insertions because nasogastric tube insertions were not recorded
REFLUX	K21 - Gastro-oesophageal reflux disease	G24.3 – Anti-reflux fundoplication using abdominal approach	Q2 2001-Q1 2020	**Count**<br>All admissions with ICD-10 diagnosis code and a procedure code detailed in prior columns	For the REFLUX trial, only surgical treatments could be captured and not conservative medical management;
EVAR	I71.4 - Abdominal aortic aneurysm, without mention of rupture<br>I71.9 - Aortic aneurysm of unspecified site, without mention of rupture<br><br>(Restricted to elective admissions)	(Excluded procedure codes labelled emergency)<br>Open heart surgery<br>L19.x - Other replacement of aneurysmal segment of aorta<br>L21.x - Other bypass of segment of aorta<br>L22.x - Attention to prosthesis of aorta<br>L49.x - Other replacement of aneurysmal iliac artery	Q2 2001-Q1 2020	**Proportion**<br>Denominator All first elective admission for an abdominal aortic aneurysm with a repair<br><br>Numerator: Admissions meeting the deominator criteria and where there was an endovascular repai	Restricted to elective admissions based on admimeth and restricted to first admission per patient

		L51.x - Other bypass of iliac artery<br>L52.x - Reconstruction of iliac artery<br>L23.1 - Plastic repair of aorta and end to end anastomosis of aorta<br>L23.2 - Plastic repair of aorta using subclavian flap<br>L23.3 - Plastic repair of aorta using patch graft<br>L23.8 - Other specified plastic repair of aorta<br>L23.9 - Unspecified plastic repair of aorta<br>L25.x - Other open operations on aorta (except L25.3 Open embolectomy of bifurcation of aorta)<br>L53.x - Other open operations on iliac artery (except L53.2 - Open embolectomy of iliac artery)<br>L65.1 - Revision of reconstruction involving aorta<br>L65.2 - Revision of reconstruction involving iliac artery<br>Endovascular repair<br>L27.x – Transluminal insertion of stent graft for aneurysmal segment of aorta<br>L28.x - Transluminal operations on aneurysmal segment of aorta<br>L26.6 - Transluminal aortic stent graft with fenestration NEC<br>L26.7 - Transluminal aortic branched stent graft NEC			

		Any of codes listed in open with the additional code of Y02.2 (Insertion of prosthesis into organ NOC)			
KAT		Denominator<br>W40.1 - Primary total prosthetic replacement of knee joint using cement<br>W40.2 - Conversion to total prosthetic replacement of knee joint using cement<br>W41.1 - Primary total prosthetic replacement of knee joint not using cement<br>W41.2 - Conversion to total prosthetic replacement of knee joint not using cement<br>W42.1 - Primary total prosthetic replacement of knee joint NEC<br>W42.2 - Conversion to total prosthetic replacement of knee joint NEC<br>O18.1 - Primary hybrid prosthetic replacement of knee joint using cement<br>O18.2 - Conversion to hybrid prosthetic replacement of knee joint using cement<br>Numerator<br>Any of the above codes **AND**<br>W58.1 - Primary resurfacing arthroplasty of joint<br>(If more than one primary procedure coded (per side) take first)	Q2 2001-Q1 2020	**Proportion**<br>Denominator: Number of total knee replacements, restricted to the first per knee per person<br><br>Numerator: Admissions meeting the denominator criteria and who also have a code relating to the resurfacing of the patella also recorded	For the KAT trial data were presented as the percentages of total knee replacements performed with resurfacing the patella, restricted to the first procedure per knee.

REACTIV & CLaSS	Not used as diagnoses not routinely recorded in outpatient setting.	Surgery<br>L84 – combined operation on vein of leg<br>L85 – ligation of vein of leg<br>L87 – other operations of vein of leg<br>EVLA<br>L88.1 - Percutaneous transluminal laser ablation of long saphenous vein<br>L88.3 - Percutaneous transluminal laser ablation of varicose vein of leg NEC<br>RFA<br>L88.2 - radiofrequency ablation of varicose vein of leg<br>Foam Sclerotherapy<br>L86.2 - Ultrasound guided foam sclerotherapy for varicose vein of leg	Q2 2006-Q12020 Non-surgical treatments of varicose veins are undertaken in outpatient setting – the earliest data available is 2006	**REACTIV Count**<br>First admission with diagnosis code of I83 (varicose veins) and the patients underwent a surgery ; excluding previous admissions with a diagnosis of varicose veins with ulceration (I83.0, I83.2)<br><br>**CLaSS Proportion**.<br>Denominator: All outpatient appointments or inpatient admissions where the patient underwent a treatment for varicose veins, with one of the OPCS codes listed. Restricted to the first treatment per patient<br>Numerator: Number of attendances with the respective OPCS4 code.	For the trials about varicose veins, CLaSS and REACTIV, we plotted the proportions of three treatments investigated in the CLaSS trial (traditional surgery, endovenous laser ablation, and foam sclerotherapy), along with a fourth called radiofrequency ablation as per the coding advice received. For REACTIV this was the first surgery performed, regardless of whether a patient had any prior non-surgical treatment.<br><br>For CLaSS if multiple types of procedure were recorded at the same time, we attributed them using the following order: Foam Sclerotherapy, EVLA, RFA, Surgery

Supplemental Materials 3. Interview Guide

OPENING QUESTIONS
Researcher: Wonderful, now to help characterise you in my report, could you describe your job title and how many years you have worked in this area?

Participant: [says job title and years in practice]

Researcher: Thank you, my records indicate that you can talk to us about one of the main recommendations made in the [HTA trial(s)], specifically that [describe recommendation]. Yes or No: Do you think that this recommendation has been taken up in NHS practice?
Participant: [yes/no]

Researcher: To assess whether this recommendation has been taken up in NHS practice, our research team has used the Hospital Episode Statistics database to trace the number of times the recommended procedure was used from 2003 through 2020. Generally, our results seem to indicate that [indicate what the HES finding seem to be saying, and show graph created].

MAIN QUESTION – ABOUT POTENTIALLY INFLUENTIAL FACTORS
Researcher: Now the main purpose of this interview is to gather your insights as to why this recommendation [has or has not] been taken up. For example, there might be something about the intervention that influenced whether it was taken up, or maybe there is something about the individuals or macro-organizations they work in. And now is when the interview should become much more flexible and guided by you. I will try to ask follow-up questions as you explain why you believe the intervention [has or has not] been taken up.

Participant: [given time to describe factors]

Researcher Probe questions to ensure all CFIR Domains are addressed, these should be used flexibly acknowledging that many of the domains may be naturally addressed by participants without prompts:

- Process: Please, describe any initiatives created to disseminate this recommendation. Who were they lead by, were there any local champions, was there any outside monitoring or feedback?
- Intervention Characteristics: Tell me about any factors related to the intervention or the study that influenced implementation. For example, maybe another treatment was developed as or after the study was conducted that was more effective than the study's recommended treatment?
- Characteristics of Individuals: Do you think that the surgeons themselves had an influence? For example, are surgeons aware of the recommendation? Do they agree with the recommendation? Why/why not?
- Inner setting: Please describe factors within the NHS's working structures that may have influenced use of this intervention? For example, in your opinion does the NHS support changes in practice like this? Who would lead this change or make it a priority for surgeons, and did they do so for this study? Why/Why not?
- Outer setting: Do you think that factors outside the NHS's working structure may have influenced whether this intervention was implemented? For example, can you describe how patients' preferences influenced its use, or any external policies or incentives that influenced its use?

CLOSING
Researcher: It has been great to speak with you today. Do you have any other questions, comments or concerns you would like to express before we end this interview?

Participant: [either offers questions to which the researcher responds or does not have any questions]

Supplemental Materials 4. Qualitative Analyses

Temporal: Events confirmed in time from participant interviews or documents reviewed.

To confirm events, the lead researcher KAS reviewed documents participants recommended and conducted internet searches. Only confirmed events are reported.

FOOD

Jan 1996 – Previous paper published

- Norton B, Homer-Ward M, Donnelly MT, Long RG, Holmes GK. A randomised prospective comparison of percutaneous endoscopic gastrostomy and nasogastric tube feeding after acute dysphagic stroke. *BMJ*. 1996 312(7022):13-6. doi: 10.1136/bmj.312.7022.13
- "*[The FOOD Trial came] following the result of the Norton, et al. paper in the BMJ, I think it was, which randomized 32 patients and found a 70% reduction in death related to use of PEG. So, that had caused in the years before we did the FOOD trial an upsurge, we felt, in enthusiasm for PEG, which really wasn't justified by the evidence, because the trial was not done well and didn't report important function outcomes. So, that particular FOOOD trial, FOOD three, was dominant in an era where there was an enthusiasm for PEG caused for a very small and not very robust studies and quite a lot of observational work.*" (-F1.Author)

March 2005 – FOOD trial first published in The Lancet

- Dennis MS, Lewis SC, Warlow C, FOOD Trial Collaboration. Routine oral nutritional supplementation for stroke patients in hospital (FOOD): a multicentre randomised controlled trial. *The Lancet*. 2005. 365(9461):755-63. doi: 10.1016/S0140-6736(05)17982-3

National Stoke Guidelines have been produced.

- 2004 – Does **not** recommend NG tubes: "Too few studies have been performed, and these have involved too few patients. PEG feeding may improve outcome and nutrition as compared with NGT feeding" https://www.google.com/url?sa=t&rct=j&q=&esrc=s&source=web&cd=&ved=2ahUKEwjFjbqR1N3zAhUUgFwKHea1D-gQFnoECAMQAQ&url=https%3A%2F%2Felearning.medistra.ac.id%2Fpluginfile.php%2F592%2Fmod_folder%2Fcontent%2F0%2FStroke%2520Guidelines%25202ed.pdf%3Fforcedownload%3D1&usg=AOvVaw1bdEwLgQzqBvajFEz5sj2o
- 2008 July – Recommends NG tube considered before PEG http://www.wales.nhs.uk/documents/RCP%20Guidelines%203rd%20Edition%2Epdf
- 2012 September – Recommends NG tube considered before PEG https://www.strokeaudit.org/Guideline/Historical-Guideline/National-Clinical-Guidelines-for-Stroke-fourth-edi.aspx
- 2016 July – recommends NG tube considered before PEG https://www.strokeaudit.org/SupportFiles/Documents/Guidelines/2016-National-Clinical-Guideline-for-Stroke-5t-(1).aspx

- "*It might have been NICE or it might have been the Intercollegiate Guidelines Network… I think the guidelines have generally gone along with the FOOD trial results, which suggest that , yep, start tube feeding early, persist as long as you can, and switch to a PEG if you need to but not in the early period.*" (-F1.Author)

February 2006 - NICE guidelines for "Nutrition support in adults Oral nutrition support, enteral tube feeding and parenteral nutrition"

- Recommends NG for short term use https://www.nice.org.uk/guidance/cg32/evidence/full-guideline-194889853. Current NICE 2017 guidelines continue to support https://www.nice.org.uk/guidance/cg32
- "*We're much more content now than we wouldn't been than to talk about palliative care for someone with stroke, with severe disability. And, also a phenomenon that's only recently, sort of actually formulated into any kind of professional description, and that's the notion of feeding at risk.*" (-F2.Surgeon)
-

August 2007 – Study supporting use of bridles published

- Beavan JR, Conroy S, Leonardi-Bee J, Bowling T, Gaynor C, Gladman J, Good D, Gorman P, Harwood R, Riley J, Sach T, Sunman W. Is looped nasogastric tube feeding more effective than conventional nasogastric tube feeding for dysphagia in acute stroke? Trials. 2007. 8:19. https://doi.org/10.1186/1745-6215-8-19
- "*The other thing that probably has influenced practice as well is a relatively small – well, the introduction of restraint to keep NG tubes in. First with things like mittens, which haven't been properly evaluated, but then there was a small trial performed in Nottingham/Derby, from memory, which looked at the effectiveness of putting a nasal loop, and that increased/improved the duration and the effectiveness of NG feeding.*" (-F1.Author)
- "*There's a lot of nursing literature, which was very much pushing against any form of restraint, seen as unethical. And I think, hopefully, we now have a more balanced view. That you've got to take a holistic view of what you're trying to achieve. And it isn't always unethical to restrain in some way to achieve better adherence to a given intervention.*" (-F1.Author)
- "*One of the cultural changes in practice that must've occurred probably about 10 years ago is the increase in the use of nasal bridles and mittens for patients as well. And both of those, I think, have increased the survivability of nasogastric tubes when being used for feeding.*" (-F2.Surgeon)

2007 – Growing recognition of stroke as a subdiscipline.

- Stroke Medicine only recognized as a subdiscipline in 2007 https://www.healthcareers.nhs.uk/explore-roles/doctors/roles-doctors/medicine/stroke-medicine
- [researcher asks why it took so long for a change in practice to start] "*At that point [when the trial was published], stroke, per se, was certainly not in this trust seen as a real speciality, it was not as high profile as maybe it is now. So certainly, here, stroke patients were cared for more for within acute medicine or within elderly care rather than as a stroke speciality. And it was only sort of after this time that stroke was a speciality, certainly in this area became more recognized.*" (-F3.Other-stroke dietician)
-

February 2009 – ACT FAST stroke campaign launched by Public Health England

- Flynn D, Ford GA, Rodgers H, Price C, Steen N, Thomson RG. A time series evaluation of the FAST National Stroke Awareness Campaign in England. *PLoS One.* 2014. 9(8):e104289. doi: 10.1371/journal.pone.0104289.

June 2010 – Stroke in adults Quality standard [QS2]

- Prompt admission to specialist acute stroke units https://www.nice.org.uk/guidance/qs2/chapter/Quality-statement-1-Prompt-admission-to-specialist-acute-stroke-units

July 2010 – General Medical Council's Treatment and care towards the end of life: good practice in decision making

- https://www.gmc-uk.org/ethical-guidance/ethical-guidance-for-doctors/treatment-and-care-towards-the-end-of-life
- *"Culturally, there's been a big change in attitudes toward managing nutrition and hydration. So, since the GMC brought out their guidelines about meeting nutritional needs and avoiding prolonging intolerable life (is that the phrase that they use), the dieticians and myself at least we use that quite a lot to try to get doctors really think about the appropriacy of non-oral feeding*." (F4.Other-Speech and Language Therapist)

January 2013 – Stroke Sentinel National Audit started

- https://www.strokeaudit.org/About-SSNAP/SSNAP-Clinical-Audit/Data-Collection.aspx
- "*The RCP Stroke Guidelines are what we follow… There's other things where we meet the stroke sentinel National audit Programme, so we try to fit in with that as well*." (-F4.Other-Speech and Language Therapist)

October 2014 – Five Year Forward View

- Recommended promoting "specialised" services using the example of stroke care https://www.england.nhs.uk/wp-content/uploads/2014/10/5yfv-web.pdf

BMJ Publishing Group Limited (BMJ) disclaims all liability and responsibility arising from any reliance placed on this supplemental material which has been supplied by the author(s)

REFLUX

Date factor occurred confirmed by independent search
**May 2011 – LOTUS trial published with similar conclusions**<br>• Galmiche JP, Hatlebakk J, Attwood S, Ell C, Fiocca R, Eklund S, Långström G, Lind T, Lundell L, LOTUS Trial Collaborators. Laparoscopic antireflux surgery vs esomeprazole treatment for chronic GERD: the LOTUS randomized clinical trial. *JAMA*. 2011. 18;305(19):1969-77. doi: 10.1001/jama.2011.626<br>• "*Certainly, as surgeons we're aware of the results [of the REFLUX trial], this alongside the LOTUS study which happened as similar sort of time…the findings were similar in terms of long-term management of reflux surgeries.*" (-Ref1.Surgeon)
**September 2012 – LINX® Reflux management system approved by NICE**<br>• https://www.nice.org.uk/guidance/ipg431<br>• "*I can't remember when the anti-reflux stuff came out about stuff like LINX®, it was a couple of years ago now.*" (-Ref2.Other-gastroentologist)
**April 2013 – REFLUX 5-year follow-up published in BMJ**<br>• Grant A M, Cotton S C, Boachie C, Ramsay C R, Krukowski Z H, Heading R C et al. Minimal access surgery compared with medical management for gastro-oesophageal reflux disease: five year follow-up of a randomised controlled trial (REFLUX). *BMJ.* 2013. 346:f1908. doi:10.1136/bmj.f1908
**2014 – NICE recommendations updated**<br>• https://www.nice.org.uk/guidance/CG184/chapter/1-Recommendations#laparoscopic-fundoplication<br>"1.10 Laparoscopic fundoplication<br>1.10.1 Consider laparoscopic fundoplication for people who have:<br>• a confirmed diagnosis of acid reflux and adequate symptom control with acid suppression therapy, but who do not wish to continue with this therapy long term<br>• a confirmed diagnosis of acid reflux and symptoms that are responding to a PPI, but who cannot tolerate acid suppression therapy. **[new 2014]**

EVAR

EVAR trial result findings - only some publications are here, meant to capture the key publications and the controversy rising after the initial trials finding.

September 2004 – EVAR 30-day mortality results published – Favour EVAR

- Greenhalgh RM, Brown LC, Kwong GPS, Powell JT, Thompson SG. Comparison of endovascular aneurysm repair with open repair in patients with abdominal aortic aneurysm (EVAR trial 1), 30-day operative mortality results: randomised controlled trial. *The Lancet,* 2004. 364(9437): 843-8. doi: 10.1016/S0140-6736(05)66627-5

  "In patients with large AAAs, treatment by EVAR reduced the 30-day operative mortality by two-thirds compared with open repair. Any change in clinical practice should await durability and longer-term results." (Quote from paper)

June 2005 – EVAR1 four year follow up published – No advantage of EVAR over open surgery.

- EVAR trial participants. Endovascular aneurysm repair versus open repair in patients with abdominal aortic aneurysm (EVAR trial 1): randomised controlled trial. *The Lancet*. 2005. 365(9478):2179-86. doi: 10.1016/S0140-6736(05)66627-5

  "…with respect to all-cause mortality and HRQL, is more expensive, and leads to a greater number of complications and reinterventions." Quote from paper)

June 2005 – EVAR2 four year follow up published – No advantage of EVAR over no surgery

- EVAR trial participants. Endovascular aneurysm repair and outcome in patients unfit for open repair of abdominal aortic aneurysm (EVAR trial 2): randomised controlled trial. *The Lancet*. 2005. 365(9478):2187-92. doi: 10.1016/S0140-6736(05)66628-7

August 2007 -– Evidence pointing to AAA not being the major cause of death for trial participants

- Greenhalgh RM, Brown LC, Powell JT. High risk and unfit for open repair are not the same. *Eur J Vasc Endovasc Surg.* 2007. 34(2):154-5.

June 2008 – EVAR authors discuss common misconceptions in the interpretation of their trial findings

- Greenhalgh RM, Brown LC. The most important misinterpretations of the UK randomised trials on abdominal aortic aneurysm repair, *Scandinavian Journal of Surgery.* 2008. 97:116-20.

10-year follow up no difference between EVAR and Open

- **April 2010 – 10-year follow-up** - United Kingdom EVAR Trial Investigators, Greenhalgh RM, Brown LC, Powell JT, Thompson SG, Epstein D, Sculpher MJ. Endovascular versus open repair of abdominal aortic aneurysm. *N Engl J Med.* 2010. 362(20):1863-71. doi: 10.1056/NEJMoa0909305
- **March 2012 - publication in HTA** - Brown L, Powell J, Thompson S, Epstein D, Sculpher M. The UK EndoVascular Aneurysm Repair (EVAR) trials: randomised trials of EVAR versus standard therapy. *Health Technology Assess.* 2012;16(9):1-128. doi: 10.3310/hta16090

15-year follow up, favours Open surgery

- **October - 2016 – The Lancet publication of 15-year follow-up** - Patel R, Sweeting MJ, Powell JT, Greenhalgh RM for the EVAR trial investigators. Endovascular versus open repair of abdominal aortic aneurysm in 15-years' follow-up of the UK endovascular aneurysm repair trial 1 (EVAR trial 1): a randomised controlled trial. *The Lancet.* 2016. 388: 2366-74. doi: 10.1016/S0140-6736(16)31135-7
- **January - 2018 publication in HTA** - Patel R, Powell JT, Sweeting MJ, Epstein DM, Barrett JK & Greenhalgh RM. The UK EndoVascular Aneurysm Repair (EVAR) randomised controlled trials: long-term follow-up and cost-effectiveness analysis. *Health Technology Assessment.* 2018;22(5): 1-132. doi: 10.3310/hta22050

OVER trial findings – similar to EVAR trial in methodology but conducted in the USA Veterans Administration

- "*An American EVAR trial kind of stated and finished a bit later and did not show the same trend, so there aren't many people who are completely convinced.*" (-E2.Surgeon)

October 2009 –OVER trials 2 year follow up favours EVAR

- Lederle FA, Freischlag JA, Kyriakides TC, et al. Outcomes Following Endovascular vs Open Repair of Abdominal Aortic Aneurysm: A Randomized Trial. *JAMA*. 2009. 302(14):1535-1542. doi:10.1001/jama.2009.1426

November 2012 – OVER trial 9 year follow up no difference between EVAR and Open surgery – EVAR has higher reintervention rates

- "Endovascular repair led to increased long-term survival among younger patients but not among older patients, for whom a greater benefit from the endovascular approach had been expected."
- Lederle FA, Freischlag JA, Kyriakides TC, Matsumura JS, Padberg FT Jr, Kohler TR, Kougias P, Jean-Claude JM, Cikrit DF, Swanson KM; OVER Veterans Affairs Cooperative Study Group. Long-term comparison of endovascular and open repair of abdominal aortic aneurysm. *N Engl J Med.* 2012. 367(21):1988-97. doi: 10.1056/NEJMoa1207481

May 2019 – OVER trial 14 year follow up no difference between EVAR and Open surgery – EVAR has higher reintervention rates

- Lederle, et al. (2019). Open versus Endovascular Repair of Abdominal Aortic Aneurysm. *N Engl J Med*. 2019. 380: 2126-2135 doi: 10.1056/NEJMoa1715955.

July 2008 - Vascunet report

- https://www.vascularsociety.org.uk/_userfiles/pages/files/Document%20Library/ESVS_VASCUNET_REPORT_2008_BW.pdf
- "*At the very early years of your graph there were Europe wide audits that showed UK to be the worst performer in terms of 30-day mortality after elective aneurysm repair.*" (-E2.Surgeon)

July 2009 – National AAA screening program phased roll out starting in July 2009 and full roll out by April 2013

• Davis M, Harris M, Earnshaw JJ. Implementation of the National Health Service Abdominal Aortic Aneurysm Screening Program in England. *Journal of Vascular Sugery.* 2013. 57(5): 1440-5. doi: 10.1016/j.jvs.2012.10.114<br>• "*The screening programme is starting to, you know, because it screens patients at 65, now we're not talking about the age group you're mentioning [from about 74 in the EVAR trials]… I've definitely seen a change in practice for these younger patients, and there's a greater preparedness to proceed with open surgery for young patients if they consent.*" (-E3.Other.radiologist)
**June 2013 – Publishing Individual-level surgeon 30-day mortality rates commences**<br>• NHS News: Major breakthrough in NHS transparency as consultant mortality data goes online for first time https://www.england.nhs.uk/2013/06/mjr-brkthgh-nhs-transp-cons/<br>• "*Very importantly, UK has also adopted individual surgeon levelled result reporting*" (-E2.Surgeon)<br>• "I know from operating surgeons, they don't like, and it's nice that they don't like it, but they don't like people to die on them. And so , if 5% of your patients or 3% are going to die from an open repair in hospital while they're under your care, even though there's a long-term price to pay, I think there's a psychological bias towards the procedure which might store up fairly in the future but doesn't happen on my short-term watch." (-E3.Other-radiologist)<br>• "*I mean, just from a purely human point of view, people don't want patients whom they know, and the families then they don't, you know, understand. And they don't want [the patient] to die under their care. And I think that influences behaviour because you know the vast, vast, vast majority of patients will come in off the EVAR and go along, and their long-term complications 10 or 15 years might well be under another hospital, another country, another physician, and so it's kind of hidden, the penalties are hidden.*" (-E3.Other.radiologist)

KAT

April 2003 – National Joint Registry started to collect data

- https://www.hqip.org.uk/national-programmes/joint-replacement-surgery-the-national-joint-registry/#.YX0LsC1Q3yU
- *"We've always been rather cautious about using HES data. And I would say the best source of data, really, would be the National Joint Registry because they record the details of every implant."* (-K1.Author)
- "*I think the only way you're going to find out if patella resurfacing is done or not and fitting it into timescale as, I would probably not use HES data, I would use NJR data*." (-K2.Other-coding expert)

April 2004 – Cochrane protocol for review of "Patella resurfacing in total knee arthroplasty" – not converted into review

- Khan RJK, Khoo P, Fick DP, Gupta RR, Jacobs W, Wood DJ. Patella resurfacing in total knee arthroplasty. *Cochrane Database of Systematic Reviews.* 2004. 2:CD004799. DOI: 10.1002/14651858.CD004799.

January 2009 – 1st paper published. KAT Trial Group,

- Johnston L, MacLennan G, McCormack K, Ramsay C, Walker A. The Knee Arthroplasty Trial (KAT) design features, baseline characteristics, and two-year functional outcomes after alternative approaches to knee replacement. *J Bone Joint Surg Am.* 2009. 91(1): 134-41. doi: 10.2106/JBJS.G.01074
- *"The first paper would have been published at about five years, from what I remember, and presumable the HTA report published at 10 years."* (-K1.Author)

April 2009 – Patient Reported Outcome Measures (PROMs) commence.

- https://digital.nhs.uk/data-and-information/data-tools-and-services/data-services/patient-reported-outcome-measures-proms
- "*I think most of the evidence would also suggest that there is no significant impact, particularly at the level of clinical importance, from patients, in terms of Patient Reported Outcome Measures and satisfaction rates*." (-K3.Surgeon)

April 2011 – National Joint Registry mandatory reporting for knee surgery commences.

- https://www.hqip.org.uk/national-programmes/joint-replacement-surgery-the-national-joint-registry/#.YX0LsC1Q3yU
- "[compared to the HES database…] *There's still some issues with the National Joint Registry data, though initially when it started in 2003 recording data onto the NJR was not mandatory, and rates of compliance where quite poor in the first few years of the registry. . Mandatory reporting only came online for the NJR I think about ten years ago*." (-K3.Surgeon)

September 2012 – First Getting it Right the First-Time report

- Briggs T. *Getting it Right First Time: improving the quality of orthopaedic care within the National Health Service in England.* London: British Orthopaedic Association; 2012.

December 2012 – NHS England (then NHS Commissioning Board) announced that through the 'Everyone Counts' initiative

- the activity and outcomes of surgery at individual consultant-level would be published by 30 June 2013 for ten clinical areas. https://www.njrcentre.org.uk/njrcentre/News-and-Events/Outcomes-publication-for-joint-replacement

April 2013 – Payment uplift

- increased payment if the knee was resurfaced by £2642. http://www.fundingrequests.cscsu.nhs.uk/wp-content/uploads/2016/11/015-patella-resurfacing-v0.1.pdf
- *"I think the HES coding bit of it is plain straight down to how the codes were applied. And for this particular one there was a change in the logic, and I've emailed you the dates because I can't remember them straight off hand, where several things happened. So, you switch to creating HRGs [Healthcare Resource Groups] out of the HES codes, which were based on the basis for the payment by results. In amongst that then came the payment system that they bolted on to that. At some point I think I've sent you the dates, they switched the logic which meant that if you coded the patella resurfacing at the time of a knee replacement you got paid an uplift of a couple thousand pounds."* (-K2.Other.coding expert)

No Month 2014 – The Orthopaedic Data Evaluation Panel starts including knees in their database

- https://www.matortho.com/orthopaedic-data-evaluation-panel-odep-2/
- "*ODEP is an independent group that I'm actually part of the sort of part that independently reviews combinations of implants.*." (-K4.Surgeon)

March 2015 – Second First Getting it Right the First-Time report

- Briggs T. *Getting it Right First Time: a national review of adult elective orthopaedic services in England.* London: British Orthopaedic Association. 2015.
- "*So there has been work with, you know, GIRFT as well. I guess I haven't mentioned the GIRFT because they've sort of been championing doing the patella. But again, mainly in a defensive way in that it reduces the risk of further operations, etc cetera. GIRFT have also championed other things such as trying to get the cost of implants down and lots of useful things that have tried to standardize care a bit more*." (-K4.Surgeon)

April 2017

- The second change, which occurred 2017/2018 which got rid of the extra payment for resurfacing.

**January 2018 – Systematic review of meta-analyses showing no functional improvements for patella resurfacing**<br>• Grassi, A., Compagnoni, R., Ferrua, P. *et al.* Patellar resurfacing versus patellar retention in primary total knee arthroplasty: a systematic review of overlapping meta-analyses. Knee Surg Sports Traumatol Arthrosc. 2018. 26**:** 3206-18. doi: 10.1007/s00167-018-4831-8<br>• "*There's lots of meta-analyses and individual RCTs in this area, and essentially, when you boil it down, and most of them will show that there is a secondary reoperation rate that is higher in patients that don't have the patella resurfaced…. So essentially what you're doing is, you're balancing this secondary reoperation rate, which is where some of the recommendations from KAT come in because of the cost associated with secondary operations.*" (K3.Surgeon)
**April 2019 – Cochrane protocol for review of "Patella resurfacing in total knee arthroplasty" – has been withdrawn**<br>• Khan RJK, Khoo P, Fick DP, Gupta RR, Jacobs WCH, Wood DJ. Patella resurfacing in total knee arthroplasty. *Cochrane Database of Systematic Reviews* 2019. 4: CD004799. doi: 10.1002/14651858.CD004799.pub2
**June 2020 – NICE Guidelines recommend resurfacing**<br>• Patella resurfacing 1.7.2 Offer resurfacing of the patella to people having primary elective total knee replacement.<br>• Guidelines show that few trials find significant functional improvements. https://www.nice.org.uk/guidance/ng157/evidence/l-patella-resurfacing-pdf-315756469335 "The NICE panel were unable to draw conclusions on whether or not to recommend selective resurfacing" (page 134) and "Given the financial impact of the findings of the KAT trial (equivalent of up to £30M a year savings) the financial impact is likely to be large" (page 135)
**No Date – American Academy of Orthopaedic Surgeons supports resurfacing patella**<br>• https://www.orthoguidelines.org/go/cpg/detail.cfm?id=1309<br>• Recommendation from Academy says that: "Practitioners should generally follow a Moderate recommendation but remain alert to new information and be sensitive to patient preferences." https://www.orthoguidelines.org/go/cpg/strength.cfm?id=1015<br>• *"Probably the most influential organisation is the American Academy of Orthopaedic Surgeons, and they've made their own recommendations partially based on our work."* (-K1.Author)

REACTIV and CLASS

**July 2000 – National waitlist targets introduced.**<br>• Push for waitlist started with "The Plan" https://webarchive.nationalarchives.gov.uk/ukgwa/+/http:/www.dh.gov.uk/en/Publicationsandstatistics/Publications/PublicationsPolicyAndGuidance/DH_4002960<br>• "*Many years ago, varicose veins, there used to be two or three-year waiting lists often for varicose veins because it was seen as non-urgent…And then what happened was that they set criteria on waiting list targets, which hospitals had to get their waiting list down. What that caused was a whole load of waiting list incentives…The reality of that was that it brought the waiting lists down, but it put lots of financial pressure on commissioners. And so, the commissioners started demand management and they started creating all these referral hurdles and referral guidelines saying , "You shouldn't be treating cosmetic varicose veins. You should only be treating them if there are leg ulcers or skin changes" and so on"… So, you'll find that around the early 2000s, PCTs at that time started producing referral guidelines for varicose veins that said, "don't refer patients unless they've got skin changes or leg ulcers.""* (-R.VV1.Author*)*
**September 2003 – Interventional procedure guidance released for Radio Frequency Ablation**<br>• NICE. *Radiofrequency ablation of varicose veins*. https://www.nice.org.uk/guidance/ipg8/chapter/1-Guidance
**March 2004 – Interventional procedure guidance released for Endovenous Laser Ablation**<br>• NICE. Endovenous laser treatment of the long saphenous vein. https://www.nice.org.uk/guidance/ipg52/chapter/1-Guidance
**2006 – Payment by Results rolled out to all trusts.**<br>• "18. PbR [Payment by Results] began in a limited way, with national tariffs for 15 HRGs in 2003 - 04 and 48 HRGs in 2004-05. The first NHS foundation trust (FT) applicants moved to the full PbR system in 2005-06 and other trusts in 2006-07." (Page 9 https://assets.publishing.service.gov.uk/government/uploads/system/uploads/attachment_data/file/213150/PbR-Simple-Guide-FINAL.pdf)
**November 2006 – bid submitted to commissioned call for CLASS trial**<br>• "The original application for this study was submitted in 2006 in response to a Health Technology Assessment (HTA) programme-commissioned call (06/45) for studies involving foam sclerotherapy" quote from Chapter 1 in HTA report https://doi.org/10.3310/hta19270
**March 2007-collection of reliable wait time data commences**<br>• https://www.england.nhs.uk/statistics/statistical-work-areas/rtt-waiting-times/
**May 2007 – Interventional procedure guidance released for sclerotherapy**<br>• NICE. Ultrasound-guided foam sclerotherapy for varicose veins. https://www.nice.org.uk/guidance/IPG217

March 2009 — McKinsey report released about procedures of low clinical value

- McKinsey for Department of Health. Achieving World Class Productivity in the NHS 2009/10 – 2013/14: Detailing the Size of the Opportunity. London. March 2009. See www.nhshistory.net/mckinsey report.pdf
- "*One of the things that's hacked vascular surgeons off is that varicose veins had been listed in NHS documents as a low-priority treatment, and that hasn't helped.*" (C.VV2-Author)

April 2009 – Patient Reported Outcome Measures (PROMs) commenced

- Routine collection of health gains for patients undergoing hip replacement, knee replacement, varicose vein and groin hernia surgery in England, based on responses to questionnaires before and after surgery. https://digital.nhs.uk/data-and-information/data-tools-and-services/data-services/patient-reported-outcome-measures-proms
- "*The issue is that Patient Reported Outcome Measures (PROMs) are not great for varicose veins surgery. Patients always get asked early doors about it, and they remember the bruising and the battering and the fact their legs were swollen and painful and they get phlebitis and things, so they tend not to like it much.*" (-R.CVV4.Surgeon)

2010. – Best practice tariff introduced

- "The introduction of best practice tariffs in 2010-11, and a commitment to expand them in future years, has seen tariffs increasingly determined by best clinical practice rather than average costs" (Page 15 https://assets.publishing.service.gov.uk/government/uploads/system/uploads/attachment_data/file/213150/PbR-Simple-Guide-FINAL.pdf)

August 2011 – Large RCT favouring Radio Frequency Ablation over laser ablation

- Rasmussen LH, Lawaetz M, Bjoern L, Vennits B, Blemings A, Eklof B. Randomized clinical trial comparing endovenous laser ablation, radiofrequency ablation, foam sclerotherapy and surgical stripping for great saphenous varicose veins. *Br J Surg.* 2011. 98(8):1079-87. doi: 10.1002/bjs.7555
-

July 2013 – NICE Guidelines published about interventional treatments for varicose veins.

- Interventional treatment 1.3.2 For people with confirmed varicose veins and truncal reflux: Offer endothermal ablation (see radiofrequency ablation of varicose veins [NICE interventional procedures guidance 8] and endovenous laser treatment of the long saphenous vein [NICE interventional procedures guidance 52]). https://www.nice.org.uk/guidance/cg168/evidence
- "*The NICE guidance by 2013 had become a hugely influential input into NHS practice, you know, very much more than many individual trials. So, you shouldn't underestimate the power of the NICE guidelines.*" (-C.VV2.Author)

September 2014 – Shorter-term results published for CLASS trial

- Brittenden, et al. A Randomized Trial Comparing Treatments for Varicose Veins *N Engl J Med.* 2014. 371:1218-27 doi: 10.1056/NEJMoa1400781

Supplemental material BMJ Publishing Group Limited (BMJ) disclaims all liability and responsibility arising from any reliance placed on this supplemental material which has been supplied by the author(s) *BMJ Qual Saf*

October 2017 – Patient Reported Outcome Measures (PROMs) ceased for veins and hernia

- https://www.england.nhs.uk/ourwork/insight/promsconsultation/

CFIR: Factors influencing implementation per trial.

BMJ Publishing Group Limited (BMJ) disclaims all liability and responsibility arising from any reliance placed on this supplemental material which has been supplied by the author(s)

indicated in red.	[illegible] finds that PEG use decreased, nearly 7 years after the trial was published			[illegible] that surgery for GORD decreased, after the trial was published			[illegible] and 2020) [illegible] evidence base.			[illegible] with the evidence base, but practice is still roughly evenly split.			[illegible] indicate quotes specifically about REACTIV. Purple quotes indicate quotes specific[illegible] about CLaSS<br>The audit of the Hospital Episode Statistics database (between 2004 and 2020) fin[illegible] of surgery for varicose veins decreased, after the trial was published.
1. **Innovation Characteristics**													
**1.A. Innovation Source** - *Perception of key stakeholders about whether the intervention is externally or internally developed.*													
**1.B. Evidence Strength** - *Stakeholders' perceptions of the quality and validity of evidence supporting the belief that the intervention will have desired outcomes.*	"The result of the third FOOD trial was somewhat unexpected, given that we did the trial, that particular arm of the trial, backup following the result of the Norton, et al. paper in the BMJ, I think it was, which randomised 32 patients and found a 70% reduction in death related to use of PEG. So that had caused in the years before we did the FOOD trial an upsurge, we felt, in enthusiasm for PEG, which really wasn't justified by the evidence because the trial was not done well and didn't report important function outcomes. So that particular FOOD trial, FOOD three, was dominant in an era where there was an enthusiasm for PEG caused by very small and not very robust studies and quite a lot of observational work." (F1.Author)<br><br>"The context of the evidence when the FOOD trial was originally drawn up was small-scale studies suggesting benefits to patients from the early placement of a PEG tube. And so that was the context in which FOOD trial occurred, but the FOOD trial convinced clinicians that that wasn't necessary, and they should wait and see if people are still chronically dysphagic or unable to swallow after a major stroke." (F2.Surgeon)<br><br>"There isn't really comparable data to FOOD, you know, from any other source, so it would be the definitive or decisive trial in influencing guideline recommendations in those - well, ever since, really, because there hasn't really been anything since. (F2.Surgeon)<br><br>"And then we've come to the trial that you've referred to, which was the PEG trial, which was, by far, the smallest [sample size]- only aye - what, 300 and something patients, randomised. And demonstrating that patients who were randomised to PEG had a worse outcome, a worse chance of being - a great chance of being dead or disabled than those who were treated initially with an NG. That was a statistically significant result. But, of course, on a relatively small sample size. So you'd always have a slight concern that that might have been a chance finding still." (F1.Author)		√	"LOTUS and REFLUX were the two big ones [trials] that we're all aware of. And I think that's probably what we used to support our practice." (Ref1.Surgeon)<br><br>"I think [the REFLUX trial] is helpful for us in that it gives us additional data to present to the patients, so basically when they're weighing up their decision making, I think it's very useful at that stage, so it might well influence more patients as to whether they want to go ahead with surgery or not." (Ref1.Surgeon)<br><br>"It [a trial] helps sort of the evidence base, helps explaining it to patients in giving them the sort of rationale behind anti reflux surgery in that if you want to have good control long term without the need to take a pill every day, surgery is the best option." (Ref1. Surgeon)<br><br>"LOTUS and REFLUX were the two big ones [trials] that we're all aware of. And I think that's probably what we used to support our practice. I don't know whether actually they- I would say they're more in a position to support our practice rather than necessarily change practice is the thing, so I would say this, this [the reflux trial] is more a study that supports what we were doing already in that the majority of patients that were referred for to us who want anti reflux surgery would have it." (Ref1.Surgeon)	X	√	"UK EVAR trials always took great pride in their appropriate power, but I don't believe that they were powered for up to 10 years. They were powered for much earlier, so that is questionable." (E2.Surgeon)<br><br>"The 30-day results which certainly, as you've alluded to, allowed EVAR to continue. We always knew that if the early results were poorer then EVAR was sort of dead in the water because we knew there were long-term penalties to pay for endovascular repair." (E3.Other-Radiologist)<br><br>"I think this study [about EVAR and Open surgery] is most closely linked to the value based clinical commissioning policy work that I do, it really fits with that, and the reason for that is that the evidence base changes over time, and that the NHS has a responsibility to commission procedures, interventions, any treatment actually that could be pharmaceutical to medications, it could be cognitive therapies. But anyway, they are meant to have an evidence base." (General1.General Practitioner)	X	√	"It is, I must say, it's fair to say that there have been lots of studies related to the patellar resurfacing, many randomised studies. But none as big as KAT or as long." (K1.Author)<br><br>"Despite the KAT study, there's a lot of other evidence, there's a lot of other randomised control trials been performed in this. So I think taking one study in isolation is, you know, is slightly unusual. So, there are a number of other RCTs from around the world that basically would demonstrate that potentially there is no significant difference whether you resurface the patella or not. So, I think most surgeons would look at the whole weight of evidence rather than just that from one study." (K3.Surgeon)<br><br>"There's lots of meta-analyses and individual RCTs in this area, and essentially, when you boil it down and most of them will show that there is a secondary reoperation rate that is higher in patients that don't have the patella resurfaced, and some people who are slightly cynical about this would say, that's because there's an opportunity to secondarily resurface the patella in that group of patients. But, I think most of the evidence would also suggest that there is no significant impact, particularly at the level of clinical importance, from patients, in terms of patient reported outcome measures and satisfaction rates." (K3.Surgeon)<br><br>"No one really understands what the role of the patella is, and by why it is there and why, how it works and the forces, but the fact that you can have a whole group of people who don't have a patella resurfacing versus a group of people who do have it, and actually their outcomes are the same, that doesn't make any sense." (K4.Surgeon)<br><br>"However, there is also the argument that, like, you know, how does patellofemoral...now, so you can do uni-compartmental knee replacement where you just resurface the patella and do the trochlea a bit, and...and so then there's this argument, well, how does that work then? Because actually if you're saying the patellar doesn't make any difference, if you just replace the patellar and do the trochlea a bit, actually you can get outcomes equivalent to knee replacement. So it's really not that much the patella, it's probably more the trochlea and the factors affecting that." (K4.Surgeon)<br><br>"It's just a hugely, sort of, spiky topic when there's not really good evidence one way or the other. And really, the only evidence that you should do it is because if you don't do it, then there's a higher rate of doing it second time around. So, it's not actually that. we know that they do that much better if you do it. It's just a defensive type of situation." (K4.Surgeon)<br><br>"I've seen these knee, shiny knee replacement go in some people, they were brilliantly and some people would go in and they just don't function. And it doesn't always make sense. You can't always predict who's going to do well and who's not going to do well." (K4.Surgeon)	X	√	[considering the varicose vein graphs for the REACTIV and CLASS trials]<br>"I guess the important thing is, you know, to think about the population and what population was that [illegible] upon. And is that applicable to the wider population of people wanting their varicose veins operated [illegible] United Kingdom? And if the populations don't match, then I wouldn't expect it to be a significant imp[illegible] (General3.General practitioner)<br><br>"What we showed in the [REACTIV] study was that surgery for varicose veins was cost-effective. An[illegible] recommended it." (R.VV1.Author)
**1.C. Relative Advantage** – *Stakeholders' perception of the advantage of implementing the intervention versus an alternative solution.*	""It's [PEG] a foreign body in your body, so it has all those inherent risks and it's something open to the outside world that wouldn't normally be open to the outside world. So obviously there's the risks of infection and things like that, but as long as you stick to the cleaning recommendations and you can, they usually sort of, you have to turn it every so often and I don't know those sort of details myself, but there's some care involved, but it should be quite straightforward once you know how to do that, and in terms of setting up the feed and things like that, it's exactly the same as an NG tube. It's just a different connection. So there's no difference in that way." (F4.Other)	X	√	"The patients have to follow quite specific dietary measures for up to six weeks afterwards [after surgery], even though it's a keyhole operation it is still an invasive procedure and needs general anaesthetic. So, there are quite a lot of factors that influence it [the choice to have surgery], and some people would rather just take an antacid medication every day and not worry about." (Ref1. Surgeon)	X		"There's a big element of patient choice here and it's how the information is presented and, you know, keyhole versus horrendous incision, patients come with a position that keyhole is better and society is moving that way so whatever the result show, if clinician presents two options, patient seemed to always want the minimal invasive option." (E3.Other-Radiologist)<br><br>"I imagine patients who don't know the downside [of EVAR], they don't hear so much about the downside and they're given a choice between massive incision intensive care or great treatment on the day and lower 30-day mortality, and people are very short-sighted in their thinking. You know, it's jam today rather than jam in 10 years that they want. So, I think patient choice is a big part, and if patients are not informed and don't understand those long-term data, and maybe don't care about the long-term data but I think that's part of it." (E3.Other-Radiologist)	X		"Although the general conclusion is it was more cost-effective to replace the patella, we found that if you didn't replace the patella, there were more reoperations in the first five years to replace the patella. But in the second five years, there were more reoperations with problems with the patella replacement. And so, there were, sort of, some caveats within it, and perhaps that has affected why it's only gone up to, say, 40, 50%." (K1.Author)<br><br>"Essentially what you're doing is, you're balancing this secondary reoperation rate, which is where some of the recommendations from KAT come in because of the costs associated with secondary operations." (K3.Surgeon)<br><br>"There is this cost-effectiveness, definite cost-effectiveness advantage of resurfacing the patella, but the story is more complicated, and the KAT has given great insights into that." (K1.Author)<br><br>"It may be there are more problems with the patella resurfacing in the longer term. And if there is a problem with the resurfacing, they tend to be catastrophic, whereas just a late resurfacing is not catastrophic. So, I think there's still a lot more to go with this trial."(K1.Author)	X		"There are a few things that have happened. One is people were quite short of resources. And so, t[illegible] down things that they could cut down, and varicose vein is seen as non-urgent, non-essential surger[illegible] secondly, in the time that it took to prepare, carry out, and report the study, new techniques were co[illegible] EVLA and radiofrequency ablation, and those have sort of taken off to a greater extent. So, the ope[illegible] treatment is now not necessarily the treatment of choice for a lot of people." (R.VV1.Author)
**1.D. Adaptability** - *The degree to which an intervention can be adapted, tailored, refined, or reinvented to meet local needs.*													
**1.E. Trialability** - *The ability to test the intervention on a small scale in the organization, and to be able to reverse course (undo implementation) if warranted.*													
**1.F. Complexity** – *Perceived difficulty of the intervention, reflected by duration, scope, radicalness, disruptiveness, centrality, and intricacy and number of steps required to implement.*	"[researcher:] So, it is quite an elongated process to place a PEG. So, to place the tube through the nose, the nasogastric tube.<br>[participant:] Yeah.<br>[researcher:] Is that a fair bit easier?<br>[participant:] We do that within 24 hours of admission if they have swallowing problems, yes.<br>[researcher:] So, that's a fair bit less conversation, right?<br>[participant:] S2. Yes, very much that." (F3.Other.Dietician)<br><br>""Obviously, it's [a PEG is] not without its risk. But generally, from our personal experience, if somebody is going to need NG feeding for longer than about four weeks, then it is probably in their best interest to have a PEG placed because, obviously, an NG tube is not without its risk. If it becomes misplaced, patient are going to aspirate on the feed. Try from a nurse's point of view to check the NG is really in the correct place can take a lot of time. And so, that can all have an effect on the decision-making there, yes." (F3.Other.Dietician)<br><br>""The issue is once an NG tube is in place, you've got to make sure it's in the right place. So, to do that, you've got to get an aspirate using a syringe to ensure that it's in and test the pH to ensure that it's in the stomach, not gone into the lungs. And often, you can't get the aspirate up, so you've got nothing to test. So, often, we have to send these patients down for chest x-ray, to ensure that the NG tube is in the correct position. And once we know that we can start feeding but the nursing staff still need to check at least every 24 hours that that tube is still in the same place. And that can often delay feeding. Because if you can't get an aspirate, you don't know it's in the right place. And again, with patients, particularly patients who, say, are diabetic who are on insulin, it's very important that the feed and insulin are commenced at the same time to control blood sugar. So, NG tubes are not without their difficulty to manage sometimes, yes, yes." (F3.Other.Dietician)<br><br>"The National Patient Safety Agency said that the bedside systems were not accurate enough, and they recommended X-rays, and that made it, therefore, difficult to implement longer-term nasogastric tubes, say in the community where you haven't got easy access to x-rays and NG tubes, etc cetera. So, you know, there's quite a lot of complication around it. Other things coming in, which make NG tube feeding problematic." (F1.Author)	X	√	"It's a short procedure, I believe it's day case or it's an overnight at worse. And form my understanding it's pretty successful, low, complication rates and it can get people off medication pretty quick, so, great, if it fixes and I don't have to med review them every year or dish out omeprazole or from it." (General4.General practitioner)<br><br>"So essentially, the surgeons will do keyhole surgery, correct the hiatus hernia, and then essentially wrap or do a wrap of muscle of usually 180 to 270 degrees at the junction between the top of the stomach and the bottom of the gullet to essentially tighten that junction and prevent reflux. And it sounds easy. And I think in terms of technical operations, it is, you know, relatively straightforward to do." (Ref2.Other-Gastroentologist)<br><br>"It is an invasive procedure, it's relatively low risk as surgical procedures go, and we often certainly here will often do them as a day case procedure." (Ref1.Surgeon)		√	"It's very, very big surgery, the problems of the patients presenting with aneurism is that often they have a lot of other comorbidities, particularly cardiac morbidities and others, which means that when you clamp their aorta you know, they can- their heart can fail, they can have heart attacks and the like. Because you're doing such major surgery on them, there is a risk of death, there's a risk of cardiac failure, chest problem, renal failure, all kinds of things quite apart from the risks of the lower limbs, so these are very big operations with a high risk." (E1.Surgeon)	X		"There is one fundamental underlying issue that the shape of a resurfaced patella is generally slightly different from the normal patella. So, some total knee replacement femoral components are designed more to work with the normal patella, whereas others are designed to work more with a replaced patella. But the designers accept that that may not always happen. So, the one designed to be replaced, to work with a normal patella is called a, sort of, patella-friendly design. It has a slightly different shape to trochlea. And we find from KAT, actually, there was no difference in outcome between those. There was no difference between patellar resurfacing and not resurfacing in either design. In other words, one design is not preferable to the other. So that is an underlying complexity, but it doesn't actually seem to make a lot of difference." (K1.Author)<br><br>""You look at joint registry and, then increasingly especially the last joint registry, the interesting findings about resurfacing seems to be a good thing, although, it is implant dependent which goes beyond the KAT trial. So, people tend to stick with what they've stuck with, and I don't think many people have changed." (K2.Other-Coding expert/surgeon)<br><br>""So ODEP is an independent group, that I'm actually part of the, sort of part of, that independently reviews combinations of implants. Now that only for knees actually started in about 2016, but basically, what it does do is it evaluates the outcomes and gives knee systems ratings for how good their survivorship is, so three years, five years, seven years, ten years, and up to fifteen years now. So, a very well-performing implant system, you know, whether certain knee, in a certain patella, in a certain insert, may get a 15A star rating, which basically means that, that 95% of them are still in 15 years down the line. And so that's a way that people can, sort of, independently check combinations of implants to see whether or not the patella actually makes a significant impact on it. But then they only really came out since 2016 and looking at your chart that looks when it starts levelling off rather than accelerating. (K4.Surgeon)<br><br>""There are lots of little things and there's lots of talk about whether you should or shouldn't [resurface]. What we do know is that certain implants influence the outcomes if you do use a patella or not and so it's better to use a patella in certain implants particularly if you're using posterior stabilized implants, it's probably slightly better." (K4.Surgeon)<br><br>""Not only is there the huge differentiation within a brand which I said can get into the 10s of 1000s. But also, then you think, well actually, the way you put it in may well influence the patella tracking as well. So, the numbers become, and the biomechanics becomes, mind boggling more than the human brain can actually, you know, really consider." (K4.Surgeon)<br><br>""The problems [with resurfacing] do seem to be implant dependent, so some implants actually you're better off replacing the patella. I think there's a couple of implants are better off you're not replacing the patella, and then there's a number in the middle where it doesn't matter too much one way or the other." (K2.Other-Coding expert/surgeon)<br><br>"" [commenting on the number of replacement parts] "The difficulty with this is, so, for a number of trials where you've looked at replacements for tibia femoral disease or total knee replacement let's say and looked at the options for that, mobile bearing / fixed bearing, patella replacement / not patella replacement, and so on, is in that group along together all those with tibia femoral disease and all those with patellofemoral disease. And the outcomes as I say will be different between the two groups, you can't guarantee with any randomisation that actually you've got those groups separated down and accounted for unless you specifically record it disease in each compartment." (K2.Other-Coding expert/surgeon)	X	√	"Surgical treatment involves general anaesthetic, and it is the other two treatments are what we say [illegible] minimally invasive." (C.VV1.Author)<br><br>"[foam sclero, endothermal ablation] could be done in a clinic setting under local anaesthetic, wherea[illegible] speaking, surgery option is done in a theatre setting under general anaesthetic, obviously involves w[illegible] cuts and involves stripping the vein whereas the other two treatments leave the vein in place and de[illegible] shrivels up. So, the surgical option has a greater recovery time, which was shown in the CLaSS trial[illegible] to the other two options, and more bruising." (C.VV1.Author)<br><br>"The other thing is that surgery usually required a general anaesthetic and all the associated complic[illegible] that, whereas endovenous treatment in 99.9% of the cases is local anaesthetic so it removes all the [illegible] complications of a general anaesthetic." (R.andC VV3.Surgeon)
**1.G.Design Quality and Packaging** - *Perceived excellence in how the intervention is bundled, presented, and assembled.*													
**1.H. Cost** - *Costs of the intervention and costs associated with implementing the intervention including investment, supply, and opportunity costs.*				"The CCG has got to be cost effective and efficient, and try and adhere as best it can to NICE guidelines, CKS, trial evidence, what have you not, does it always 100% happen all the time? Probably not, no. I don't think the CCG- bear in mind it's not a profit organisation, it's not trying to make a profit at the end of the year, it's not trying to pay off its shareholders what have you not, it's one and only remit is to pay for medical care for its constituent, and with an ever-shrinking budget, it just gets harder and harder." (General4.General practitioner)<br><br>"They're [the surgeries we commission are] meant to be value for money which is very important, so, even I remember back in medical school they were banging on about the cost per QUALY, there's a measurement of bang for your buck and it's still used today. So when I look at this, I think they are pointing to the right thing to do, each study [the trials: EVAR, KAT, REFLUX, CLaSS and REACTIV], but from a commissioner's point of view, it's not just about it's better for patients but that it's also value for money for the commissioning pot which is finite and it's not a bottomless pit, because if we spent say £1 million on one person for something that would be nice to have say following a large media campaign for them, we wouldn't do that by the way, but unfortunately that would remove funds for other people who could have money spent on them for all procedures that are evidenced based and shown to be good value for money," (General1.General practitioner)<br><br>"Bottom line is if the medical conservative therapy, omeprazole, lansoprazole, whatever, it's relatively cheap as chips, and I wouldn't say we quite dish it out like smarties but it's a nice easy, fix." (General4.General practitioner)<br><br>"I must admit I've got no idea of the costings or the like which is the end of the day is what certainly at CCG level is what they are bothered about. Ultimately, I think it's going to be a very simple mathematics. Does the cost of the procedure, is it more or less than a lifetime of conservative management, and I must admit I'm not the right person probably to answer that but I suspect that is what it is ultimately going to mostly boils down to." (General4.General practitioner)<br><br>"A lot of surgeons feel that they're being stopped from doing stuff by funding restrictions imposed by GPs, which we do really not have anything - any influence over. I suppose a handful of GPs do, but even then, you're operating within very strict guidelines from the finance departments and the Department of Health. And, ultimately, once something isn't funded, it's very unlikely it's going to be funded. You know, it's more about taking things that are funded and funding them, rather than things that aren't funded and then funding them, so." (General2.General practitioner)	X		"The CCG has got to be cost effective and efficient, and try and adhere as best it can to NICE guidelines, CKS, trial evidence, what have you not, does it always 100% happen all the time? Probably not, no. I don't think the CCG- bear in mind it's not a profit organisation, it's not trying to make a profit at the end of the year, it's not trying to pay off its shareholders what have you not, it's one and only remit is to pay for medical care for its constituent, and with an ever-shrinking budget, it just gets harder and harder." (General4.General practitioner)<br><br>"They're [the surgeries we commission are] meant to be value for money which is very important, so, even I remember back in medical school they were banging on about the cost per QUALY, there's a measurement of bang for your buck and it's still used today. So when I look at this, I think they are pointing to the right thing to do, each study [the trials: EVAR, KAT, REFLUX, CLaSS and REACTIV], but from a commissioner's point of view, it's not just about it's better for patients but that it's also value for money for the commissioning pot which is finite and it's not a bottomless pit, because if we spent say £1 million on one person for something that would be nice to have say following a large media campaign for them, we wouldn't do that by the way, but unfortunately that would remove funds for other people who could have money spent on them for all procedures that are evidenced based and shown to be good value for money," (General1.General practitioner)<br><br>"[talking about EVAR trials] I just think we live in a time where costings is everything and I go back to the CCG argument, at the end of the day the CCG has a finance amount of money, it has an increasing health burden, an older population. Ill people can be expensive, and we are treating people with statins so they do live longer. We do keep on top of blood pressures. We are not terrible, despite what everyone thinks, I think at picking up cancers early, and people are living longer and longer. The trouble is then they go back to the nursing home that I look after. It's effectively a nursing and dementia home. And we've got well 90-year-olds will be with dementia and all of a sudden, they have to sell houses and what have you not for their care and it's, well, it's very topical today, obviously with all the announcements being made. But I think funding and cost will become an increasing, increasing topic of conversation to the point where how much longer seriously can a nationalised health service really continue. And as much as it breaks mine and a lot of NHS workers hearts to say, I think sooner or later one government is going to have to have a serious conversation with the electorate to say "we simply cannot afford this anymore, we're going to have to ask you to start paying." We pay for prescriptions, we pay for dental care, we pay for opticians, the next thing that has to go that's [illegible]	X	√	"The CCG has got to be cost effective and efficient, and try and adhere as best it can to NICE guidelines, CKS, trial evidence, what have you not, does it always 100% happen all the time? Probably not, no. I don't think the CCG- bear in mind it's not a profit organisation, it's not trying to make a profit at the end of the year, it's not trying to pay off its shareholders what have you not, it's one and only remit is to pay for medical care for its constituent, and with an ever-shrinking budget, it just gets harder and harder." (General4.General practitioner)<br><br>"They're [the surgeries we commission are] meant to be value for money which is very important, so, even I remember back in medical school they were banging on about the cost per QUALY, there's a measurement of bang for your buck and it's still used today. So when I look at this, I think they are pointing to the right thing to do, each study [the trials: EVAR, KAT, REFLUX, CLaSS and REACTIV], but from a commissioner's point of view, it's not just about it's better for patients but that it's also value for money for the commissioning pot which is finite and it's not a bottomless pit, because if we spent say £1 million on one person for something that would be nice to have say following a large media campaign for them, we wouldn't do that by the way, but unfortunately that would remove funds for other people who could have money spent on them for all procedures that are evidenced based and shown to be good value for money," (General1.General practitioner)<br><br>"Taken that there is this cost-effectiveness, a definite cost-effectiveness advantage of resurfacing the patella, but the story is more complicated, and the KAT has given great insights into that." (K1.Author)<br><br>"Essentially what you're doing is, you're balancing this secondary reoperation rate, which is where some of the recommendations from KAT come in because of the costs associated with secondary operations." (K3.Surgeon)<br><br>"[Participant:] When you look at rates of resurfacing across the world, there is quite wide variation in what goes on, and actually if you look at the I think the Swedish Knee Arthroplasty Register would be worth looking at. I'm fairly certain that their rates of resurfacing were higher and have gone down over the same period of time.<br>[Researcher:] Uh-huh.<br>[Participant:] So, when I last looked, and this was quite a few years ago, their rate of resurfacing was about less than 10 per cent.<br>[Researcher:] Oh, wow.<br>[Participant:] Whereas in the US you know, the rate of resurfacing is three quarters, 80 per cent, and again some of it comes down to coding, reimbursement, uplifts and how people are paid. So, all of these factors influence the rate of resurfacing, possibly more so than evidence from one RCT." (K3.Surgeon)		√	"The CCG has got to be cost effective and efficient, and try and adhere as best it can to NICE guidel[illegible] trial evidence, what have you not, does it always 100% happen all the time? Probably not, no. I don'[illegible] CCG- bear in mind it's not a profit organisation, it's not trying to make a profit at the end of the year, i[illegible] trying to pay off its shareholders what have you not, it's one and only remit is to pay for medical care [illegible] constituent, and with an ever-shrinking budget, it just gets harder and harder." (General4.General pr[illegible]<br><br>"They're [the surgeries we commission are] meant to be value for money which is very important, so, [illegible] remember back in medical school they were banging on about the cost per QUALY, there's a measu[illegible] bang for your buck and it's still used today. So when I look at this, I think they are pointing to the righ[illegible] do, each study [the trials: EVAR, KAT, REFLUX, CLaSS and REACTIV], but from a commissioner's [illegible] view, it's not just about it's better for patients but that it's also value for money for the commissioning [illegible] is finite and it's not a bottomless pit, because if we spent say £1 million on one person for something [illegible] be nice to have say following a large media campaign for them, we wouldn't do that by the way, but [illegible] unfortunately that would remove funds for other people who could have money spent on them for all [illegible] that are evidenced based and shown to be good value for money," (General1.General practitioner)<br><br>"I'm happy to talk about the funding issues around varicose veins. I suspect the plummet in the num[illegible] procedures done is because of the restriction of funding in the NHS over the last, kind of, 18 years." (General2.General practitioner)<br><br>"Over time, you know, as funding constrictions get tighter and tighter, the threshold for doing procedu[illegible] higher and higher." (General2.General practitioner)<br><br>"There are a few things that have happened. One is people were quite short of resources. And so, th[illegible] down things that they could cut down, and varicose vein is seen as non-urgent, non-essential surger[illegible] secondly, in the time that it took to prepare, carry out, and report the study, new techniques were co[illegible] EVLA and radiofrequency ablation, and those have sort of taken off to a greater extent. So, the oper[illegible] treatment is now not necessarily the treatment of choice for a lot of people.(R.VV1.Author)<br><br>"Fundamentally, decisions in the NHS are often not made on cost-effectiveness grounds. You know, [illegible] makes decisions about what it recommends, it often will make cost-effectiveness decisions. But whe[illegible] purchasers decide what they're going to buy, and they're short of money, then they cut the things the[illegible] rather than cut the things that are not cost-effective." (R.VV1.Author)<br><br>"What happened about 2009, 2010, people were very short of money, and throughout a lot of the co[illegible] commissioners suddenly started restricting varicose vein treatments." (R&CVV2.Author)<br><br>"NICE also bases some of its decisions on cost-effectiveness, and clinicians aren't in general interes[illegible]

BMJ Publishing Group Limited (BMJ) disclaims all liability and responsibility arising from any reliance placed on this supplemental material which has been supplied by the author(s)

							investment. And I can see, from a political point of view, that, for the NHS to be seen to be in the forefront of new treatments, you know, looks good for the politicians, but only if those new treatments don't come a cropper. And of course, if there are situations where, you know, various endovascular model, graphs and techniques don't work and are found to be not to provide the benefit that they're sought to provide, you know, that only causes of political impact if it gets into the press, and there, and there's a big storm about it. And unfortunately, these issues are probably not big enough, on the radar of, of the ordinary man to make it a big issue. And so the government continues to sort of, you know, turn a blind eye to, to the sort of very rapid development of things that not aren't necessarily, of value." (RandCVV5.Surgeon)			that actually the cost becomes an issue whereas was actually the individual cost per patient is actually relatively minimal." (K4.Surgeon)<br><br>"Knee replacement and the hip replacement are famously known as some of the best cost for quality, cost per QUALY procedures around and they have been for decades, so we understand that. And I guess the resurfacing of the patella just adds a bit of a detail to that and just makes a knee replacement even better, it's already a good operation if it's done on the right patients." (General1.General practitioner)<br><br>""I think the main thing is that with patella resurfacing, although it is more cost effective, I don't think the numbers are that massively different which is, you know, if you look at it on a global population level yeah, it's pretty crazy you know, you talk about, you know, millions and millions. But that's only because we do 100,000 new placements every year. So, see, right because the scale is higher that actually the cost becomes an issue whereas was actually the individual cost per patient is actually relatively minimal." (K4.Surgeon)			
**2. Outer Setting**													
2.A Needs and resources of those served - *The extent to which patient needs, as well as barriers and facilitators to meet those needs, are accurately known and prioritized by the organization.*	"The majority of our patients are still elderly patients, so eighty, ninety, maybe seventy to ninety, but over the twenty-four years that I've been a speech therapist it's definitely shifted, so we do have younger patients. So, I remember when I was in my first job. I had a 28-year-old, and that was so unusual and everybody wanted to know about it because they'd never met somebody that young and it was just oh, it's because of the lifestyle. And now that's, I wouldn't say a common occurrence, but it's definitely more frequent. We probably have at least one younger person on our stroke wards all the time." (F4.Other.Speech and Language Therapist)<br><br>""Occasionally, we have living wills, advanced decision-making may appear. Sometimes, families have previous experience of PEG tubes, and that can influence decision-making. Also, I think the biggest thing that affects our decision-making is other comorbidities that these stroke patients may come in with. So, I think one of the biggest ones would necessarily be patients with dementia." (F3.Other.Dietician)<br><br>""Families are against these kind of surgical interventions, purely for the purpose of prolonging life. Maybe that reflects - I mean, it's hard to tell when you're a medic because you have a, sort of, biased sample. But maybe that reflects popular discourse about the rights and wrongs of living with severe disability, you know. Nowadays, talk about people with motor neurone disease going to Switzerland to end their lives, etcetera, etcetera. The issue of what quality of life you might be like living with severe disability is much more out in the open, I think. And I think that means that patients and their families have more of a view about what's in their best interest of their loved one. And, of course, in a good way, medicine has become much more aware of its responsibilities to establish the needs and wants of patients in relation to severe disability. That might've been the case in the, sort of, paternalistic era in the past. And I think that opening up of debate about whether or not this is really something that your mother would've wanted. Does she want us to put her through a surgical procedure in order that she can have a permanent plastic feeding tube into her stomach so that she can be kept artificially alive? Or should we actually be letting nature take its course? And I think that sort of conversation occurs more frequently now than it used to in the past. (F2.Surgeon)	X	√	"Bottom line is if the medical conservative therapy, omeprazole, lansoprazole, whatever, it's relatively cheap as chips, and I wouldn't say we quite dish it out like smarties but it's a nice easy, fix, and for 90-odd% of the patients it seems to work." (General4.General practitioner)<br><br>"At the end of the day what my patients want to be able to do and I should probably say that I work in, which is quiet an affluent area, people enjoy their big meals out and their bowls of rich red wine and this, that and the other. They want to be able to go out on Friday night, have a nice big meal, have a bottle of rich Bordeaux and not feel horrendous the next day, so they're not coming to me but I don't think I've ever been asked direct, "Can I not have surgery for it?" I don't know, people come all the time and say, "I'd like a new knee" or, "I'd like a new heart valve" or what have you not. Reflux isn't that kind of, it's not a want as such. (General4.General practitioner)<br><br>"Quite often, you know, I've seen patients and follow up clinic where the juniors have, you know, switch to PPI. And you're kind of sitting there thinking, what's the point of doing all this? You know, they've clearly got awful reflux, you know, they've probably been there and done all this already in primary care; we've just wasted, you know, six months of their time by not having referred them to a surgeon." (Ref2.Other-Gastroentologist)<br><br>"REFLUX is interesting because very, very few patients have surgery for reflux, considering the vast quantities of patients who take anti-reflux medication." (General2.General practitioner)<br><br>"With reflux, lifestyle factors are a massive influence to it. I don't know what their lifestyle - I don't know if they'll only operate on slim people - slim non-smokers who don't drink caffeine, or is it - I don't know what their criteria for surgery are, but there is that thing that, you know, 40 years ago no had reflux, whereas now, everyone seems to have reflux, so I'm not entirely sure what's happening. (General2.General practitioner)<br><br>"Reflux is a different kettle of fish [compared to varicose veins] altogether because you're talking about a different cohort of patients. Because you're talking about, you know, people probably who are overweight, who probably do have other lifestyle problems. And do they feel underserved? I don't know. I don't know. They probably acknowledge that it's a lot of their own, you know, things that are - I haven't really had anyone complaining about their lack of surgery to sort their reflux." (General2.General practitioner)<br><br>"Reflux symptoms are one of the most common things GPs see. There is no way we could offer anti reflux surgery for every patient in the country who has reflux and it wouldn't necessarily be the right thing to do for them." (Ref1.Surgeon)<br><br>"The patients have to follow quite specific dietary measures for up to six weeks afterwards [after surgery], even though it's a keyhole operation it is still an invasive procedure and needs general anaesthetic. So, there are quite a lot of factors that influence it [the choice to have surgery], and some people would rather just take an antacid medication every day and not worry about." (Ref1. Surgeon)	X		"The age group [of people affected by AAAs] is elderly and they are at risk of death which is the only thing completely inevitable but it's the inevitable on point for anyone who ever lived, and so actually it is questionable whether all-cause mortality is actually an accurate indicator of efficacy of a particular disease specific treatment modality. However, it is important to distinguish that from the importance of measuring all-cause mortality because without that we cannot actually establish the value of the treatment in actually achieving one of the aims and also very important for United Kingdom, less important for United States et cetera which is the health economic analysis and the willingness to pay in the publically funded healthcare systems. So if you are- if you are measuring the efficacy of endovascular repair which is a disease specific treatment in terms of its ability to stop the patient from coming to harm from that particular disease, all-cause mortality has really no value because it is only specific mortality you should look for. However, to our view, that I am not going to look at all-cause mortality is untenable that is because the only reason why you repair an aneurysm is to stop a premature death and death from however method it may occur has a relevance to the utility of your operation in the overall aim." (E2.Surgeon)<br><br>"The fact that there is no real sustained mortality benefit that three years to be interpreted as no benefit is wrong because the area and the repair is greater from that group of patients. If somebody comes and puts a gun to my head and says that he determined that my three-year survival is going to be zero, I can choose whether to die now or in three years I know what I will choose, to argue that because there is no overall survival benefits sustained beyond three years to mean that there is no survival benefit at all is a wrong interpretation of the data which went on for a long period of time." (E2.Surgeon)<br><br>"The screening programme starting to, you know, because it screens patients at 65, now we're not talking about the age group you're mentioning. And I've certainly seen in my, you know, exposure to EVAR over the years, I've definitely seen a change in practise for these younger patients coming in and there's a greater preparedness to proceed with open surgery for young patients if they consent." (E3.Radiologist)<br><br>"It's very, very big surgery, the problems of the patients presenting with aneurism is that often they have a lot of other comorbidities, particularly cardiac morbidities and others, which means that when you clamp their aorta you know, they can- their heart can fail, they can have heart attacks and the like. Because you're doing such major surgery on them, there is a risk of death, there's a risk of cardiac failure, chest problem, renal failure, all kinds of things quite apart from the risks of the lower limbs, so these are very big operations with a high risk." (E1.Surgeon)<br><br>"The EVAR trials are very interesting because that's an ethical conundrum, isn't it, for the health service because it has to, look this is why in the NHS it comes down to good public health analysis, so our public health colleagues are the people who really sit behind the value based clinical commissioning policy document and check it's evidenced based nationally and internationally, and make recommendations based on not just evidence in terms of it's better for people but also cost effectiveness, so we have to make choices." (General1.General practitioner)	X	√				"There has been a sort of impression among many of those running the NHS and payers that varicos sort of subtly unimportant that ladies have done for cosmetic reasons, which is completely incorrect. remains a persistent sort of impression among many people who aren't in the specialty of making str decisions." (R.and.C.VV2.Author)<br><br>"For the great majority of people, they [varicose veins] cause no trouble. Some people just don't like cosmetic. Then some people get symptoms, which can be quite troublesome. And most important t people then go on with a high pressure in the veins for reasons we don't quite understand to develop their skin and the tissues beneath the skin in the lower leg. And that starts off with eczema. It may s eczema. It can just start with discoloration of the skin, which worsens. Then the tissues beneath the subcutaneous tissues become indurated and hard. And once you got this discoloured skin and hard tissue beneath it, that is called lipodermatosclerosis. And if you allow that to worsen, then one day, i And you can get an ulcer, which is both chronic, painful, awful, and very expensive for the health ser (R.and.C.VV2.Author)<br><br>[considering the varicose vein graphs for the REACTIV and CLASS trials]<br>"I guess the important thing is, you know, to think about the population and what population was that upon. And is that applicable to the wider population of people wanting their varicose veins operated United Kingdom? And if the populations don't match, then I wouldn't expect it to be a significant imp (General3.General practitioner)<br><br>"It's the type of patient sort of you're not seeing, so you know, you would have seen if you looked at t beginning of that graph, you'd see people with fairly uncomplicated varicose veins which is straightfo being referred and then overtime, not only do the numbers go down but the types of patients who are referred varies. So, you're going to get more and more people with the complicated varicose veins." (C.VV1.Author)<br><br>"We [commissioners] have to throughout our value-based commissioning policy have an exclusion o health throughout that. Which means that in order to apply for exceptionality to this, exceptionality c based on how a patient feels about the condition. Otherwise, everyone would feel very upset about varicose veins and the cosmetic procedure would, you know, be a nil excuse really." (R.and.CC6.Ge practitioner)<br><br>"With [EVLA and RFT] you're destroying that vein rather than stripping it surgically. And that means of a surgical incision. It means [that with endothermal ablation] you generally don't need a general ar And all of that is advantageous to the patient." (R.and.C.VV2.Author)<br><br>"One of the advantages of lasers, it is that patients perceive an advantage in terms of a quicker retur And that's of significant importance to them. I think the previous treatment with open surgery, genera kept them off work for two weeks. And that's now been replaced by a return to work within a few day endovenous laser So I think there are some significant advantages to patients in terms of their reco which they like, by avoiding a high time." (R.and.CVV5.Surgeon)
**2.B. Cosmopolitanism** *-The degree to which an organization is networked with other external organizations.*													
**2.C. Peer Pressure** - *Mimetic or competitive pressure to implement an intervention; typically because most or other key peer or competing organizations have already implemented or are in a bid for a competitive edge.*										""[Participant:] When you look at rates of resurfacing across the world, there is quite wide variation in what goes on, and actually if you look at the I think the Swedish Knee Arthroplasty Register would be worth looking at. I'm fairly certain that their rates of resurfacing were higher and have gone down over the same period of time.<br>[Researcher:] Uh-huh.<br>[Participant:] So, when I last looked, and this was quite a few years ago, their rate of resurfacing was about less than 10 per cent.<br>[Researcher:] Oh, wow.<br>[Participant:] whereas in the US, you know, the rate of resurfacing is three quarters, 80 per cent, and again some of it comes down to coding, reimbursement, uplifts and how people are paid. So, all of these factors influence the rate of resurfacing, possibly more so than evidence from one RCT." (K3.Surgeon)<br><br>""I think that surgeons by their nature are a creature of habit in their training. So you may find which you could probably dig down to in some way, shape, or form within the HES data is there may well be quite significant geographic variations within the UK with rates of resurfacing. Certainly, when I was training in the [location redacted], I would say, I worked for a number of different consultants, and I saw many of them resurfacing the patella. And consequently, when I started as a consultant, my rate of patella resurfacing was probably about 10%, 5%, very little. And it's gone up in recent years because I've seen some problems related to not resurfacing the patella, so I was selective in resurfacing in a group of patients. But I'm probably still only at about 30%. I think there's very few people that I know that resurface the kneecap in everybody. But you might find that's different in other regions of the country. So, I think it's a product of what you see, what you do, and how you've been trained." (K3.Surgeon)<br><br>""Patella resurfacing is a really, sort of, difficult topic and the reason is because there's such widespread variation and often it's more geographical variation rather than, you know, anything in particular." (K4.Surgeon)<br><br>""The striking thing is that there are certain Scandinavian countries that have zero percent patella resurfacing rate and certain Scandinavian countries that have a 100% resurfacing rates." (K4.Surgeon)<br><br>""I work in in Exeter, which I started in 2015, we have a pretty much 98-99% resurfacing rate, but our colleagues up in Edinburgh have about a, you know, between two and five percent resurfacing rates, and we use the same implant and do the same things essentially, it's just a striking difference." (K4.Surgeon)	X	√	
**2.D. External Policies & Incentives** – A broad construct that includes external strategies to spread interventions, including policy and regulations (governmental or other central entity), external mandates, recommendations and guidelines, pay-for-performance, collaboratives, and public or benchmark reporting.	"It's typical that it takes quite a long time for the results of trials to affect practice. Partly because - and you don't have this on the graph. You don't have when the trial - when guidelines changed in response. And I can't remember quite - certainly the 2016 guidelines acknowledged the FOOD trials." (F1.Author)<br><br>"I think the guidelines have generally gone along with the FOOD trial results, which suggests that, yep, start tube feeding early, persist as long as you can, and switch to a PEG if you need to, but not in the early period. (F1.Author)<br><br>"There was a national clinical guideline for the UK published in 2008, and again in 2012, and again in 2016. And all of those would've used the FOOD trial as evidence underpinning the recommendations for the management of dysphasia post-stroke." (F2.Surgeon)<br><br>"For as long as I can remember and we follow the RCP guidelines, so we wouldn't put a PEG in until at least four weeks post stroke, and sometimes longer if they're showing signs of recovery." (F4.Other.Speech and Language Therapist)<br><br>"Culturally there's been a big change in attitudes towards managing nutrition and hydration. So, since the GMC brought out their guidelines about meeting nutritional needs and avoiding prolonging intolerable life, is that phrase that they used, the dieticians and myself at least, we use that quite a lot and try and get doctors to really think about the appropriacy of non-oral feeding. (F4.Other.Speech and Language Therapist)<br><br>"The Royal College of Physicians and also the Royal College of Speech and Language Therapists have produced probably, again, over the last, sort of, six or eight years, more practice guidance about this notion of feeding at risk, which isn't something that we would've been doing in the time that the food trial was being performed." (F2.Surgeon)<br><br>"The National Patient Safety Agency said that the bedside systems were not accurate enough, and they recommended X-rays, and that made it, therefore, difficult to implement longer-term nasogastric tubes, say in the community where you haven't got easy access to x-rays and NG tubes, etc cetera. So, you know, there's quite a lot of complication around it. Other things coming in, which make NG tube feeding problematic." (F1.Author)	X	√	"The CCG has got to be cost effective and efficient, and try and adhere as best it can to NICE guidelines, CKS, trial evidence, what have you not, does it always 100% happen all the time? Probably not, no. I don't think the CCG- bear in mind it's not a profit organisation, it's not trying to make a profit at the end of the year, it's not trying to pay off its shareholders what have you not, it's one and only remit is to pay for medical care for it's constituent, and with an ever shrinking budget, it just gets harder and harder. (General4.General practitioner)<br><br>"You've missed out on probably the most influential layer and that's the CCG layer. Bottom line is if the medical conservative therapy, omeprazole, lansoprazole, whatever, it's relatively cheap as chips, and I wouldn't say we quite dish it out like smarties but it's a nice easy, fix, and for 90-odd% of the patients it seems to work." (General4.General practitioner)<br><br>"I can't off at the top of my head that we've got a GORD pathway, and in many ways it's kind of try medical management to the patients effectively keeling over in front of you with reflux then refer them in. I mean, you've got to be careful of all your red flags and you don't want to miss a cancer or the like but assuming that's all been ruled out, it's not within the CCG's radar and broadcast message to say you should be referring them up as such." (General4.General practitioner)<br><br>"I must admit I've got no idea of the costings or the like which is the end of the day is what certainly at CCG level is what they are bothered about. Ultimately, I think it's going to be a very simple mathematics. Does the cost of the procedure, is it more or less than a lifetime of conservative management, and I must admit I'm not the right person probably to answer that but I suspect that is what it is ultimately going to mostly boils down to." (General4.General practitioner)<br><br>"Probably the most influential body on my day-to-day practice is the CCG. They put their agreed guidelines, agreed pathways down, and on the whole, they seem to work and they seem to be fair. (General4.General practitioner)<br><br>"A lot of surgeons feel that they're being stopped from doing stuff by funding restrictions imposed by GPs, which we do really not have anything - any influence over. I suppose a handful of GPs do, but even then, you're operating within very strict guidelines from the finance departments and the Department of Health. And, ultimately, once something isn't funded, it's very unlikely it's going to be funded. You know, it's more about taking things that are funded and funding them, rather than things that aren't funded and then funding them, so." (General2.General practitioner)	X		"The CCG has got to be cost effective and efficient, and try and adhere as best it can to NICE guidelines, CKS, trial evidence, what have you not, does it always 100% happen all the time? Probably not, no. I don't think the CCG- bear in mind it's not a profit organisation, it's not trying to make a profit at the end of the year, it's not trying to pay off its shareholders what have you not, it's one and only remit is to pay for medical care for its constituent, and with an ever-shrinking budget, it just gets harder and harder." (General4.General practitioner)<br><br>"They're [the surgeries we commission are] meant to be value for money, which is very important, so, even I remember back in medical school they were banging on about the cost per quali, there's a measurement of bang for your buck and it's still used today. So when I look at this, I think they are pointing to the right thing to do, each study [the trials: EVAR, KAT, REFLUX, CLaSS and REACTIV], but from a commissioner's point of view, it's not just about it's better for patients but that it's also value for money for the commissioning pot which is finite and it's not a bottomless pit, because if we spent say £1 million on one person for something that would be nice to have say following a large media campaign for them, we wouldn't do that by the way, but unfortunately that would remove funds for other people who could have money spent on them for all procedures that are evidenced based and shown to be good value for money," (General1.General practitioner)<br><br>"I just think we live in a time where costings is everything and I go back to the CCG argument, at the end of the day the CCG has a finance amount of money, it has an increasing health burden, an older population. Ill people can be expensive, and we are treating people with statins so they do live longer. We do keep on top of blood pressures. We are not terrible, despite what everyone thinks, I think at picking up cancers early, and people are living longer and longer. The trouble is then they go back to the nursing home that I look after. It's effectively a nursing and dementia home. And we've got well 90-year-olds will be with dementia and all of a sudden, they have to sell houses and what have you not for their care and it's, well, it's very topical today, obviously with all the announcements being made. But I think funding and cost will become an increasing, increasing topic of conversation to the point where how much longer seriously can a nationalised health service really continue. And as much as it breaks mine and a lot of NHS workers hearts to say, I think sooner or later one government is going to have to have a serious conversation with the electorate to say "we simply cannot afford this anymore, we're going to have to ask you to start paying." We pay for prescriptions, we pay for dental care, we pay for opticians, the next thing that has to go that's going to be medical care. I'm sure of it. (General4.General practitioner)<br><br>""Soon after that [the EVAR 15-year outcomes were published], several, a few months after, that came a consultation document of NICE guidelines would try to enforce that [the results of the 15-year follow up] into or put that, incorporate that, into clinical guidelines which created quite a lot of controversy and physicians got extremely excided about it. And that resulted in a lot of debate and representations to the NICE saying that that recommendation is actually it is not possible to implement it for various different reasons to the extent that one of the rare things to happen was the NICE have actually amended the guidelines to the extent that practically they lost their teeth." (E2.Surgeon)<br><br>"At the very early years of your graph there, there are Europe wide audits that showed UK to be the worst performer in terms of 30-day mortality after elective aneurysm repair because 10 to 12% mortality they're worse in the United Kingdom than Europe." (E2.Surgeon)<br><br>""I think, in our own practise, it's [15-year follow up] led to a recalibration of decision making to feel that patients who look likely to live for 10 or 15 years are better served with an open repair approach, but I think NICE Guideline said they should be offered both, but they've [patients have] got to be aware of the implications of one choice versus another." (E3.Radiologist)<br><br>""To my understanding, the NICE Guideline say that patients have to be offered both choices and, you know, so it'll be common that a patient might be of an age for some who would say you know what, we think you're better served with an open repair but if the patient chooses endovascular repair then, you know, they're not dining entirely ala carte from the NHS. But, you know, if they express a preference for one thing over the other and it's a strong one despite information, then that will proceed." (E3.Radiologist)<br><br>"It is very easy to confuse that with the EVAR trial results because in a sense, what the NICE guidelines did was to take the EVAR trial results as it's text rather than being greatly influenced by other peripheral evidence such as it was on quality of life and other issues. So I just think you need to be aware that that has really polarised people and authored their thinking since all the controversy of the draft NICE guidelines, which occurred quite soon after the 15 year report." (E1.Surgeon)<br><br>"For any vascular specialist at the health service, I mean they don't give a monkey's *** actually about cost, they do what they think is right for each patient unless	X	√	"The CCG has got to be cost effective and efficient, and try and adhere as best it can to NICE guidelines, CKS, trial evidence, what have you not, does it always 100% happen all the time? Probably not, no. I don't think the CCG- bear in mind it's not a profit organisation, it's not trying to make a profit at the end of the year, it's not trying to pay off its shareholders what have you not, it's one and only remit is to pay for medical care for its constituent, and with an ever-shrinking budget, it just gets harder and harder." (General4.General practitioner)<br><br>"They're [the surgeries we commission are] meant to be value for money, which is very important, so, even I remember back in medical school they were banging on about the cost per quali, there's a measurement of bang for your buck and it's still used today. So when I look at this, I think they are pointing to the right thing to do, each study [the trials: EVAR, KAT, REFLUX, CLaSS and REACTIV], but from a commissioner's point of view, it's not just about it's better for patients but that it's also value for money for the commissioning pot which is finite and it's not a bottomless pit, because if we spent say £1 million on one person for something that would be nice to have say following a large media campaign for them, we wouldn't do that by the way, but unfortunately that would remove funds for other people who could have money spent on them for all procedures that are evidenced based and shown to be good value for money," (General1.General practitioner)<br><br>""They're [American surgeons are] slightly more defensive medical sort of fraternity probably from a joint replacement point of view. So, they feel as though, well, actually, you know, we've got to do it, because if you don't do it, you know, they might sue us for not doing it. And there's also a situation in America, which is slightly different from the UK, where you might have a unit down the road who are actually doing second operations on your cases because everyone's paid to do cases. You know, if you're paid to do cases, don't be surprised if you go see a surgeon and end up with an operation, whereas, the National Health Service is slightly different in that we're not paid to do cases so we try, and you could probably say a little bit more, there's a bit more, sort of, you know, objectivity to it, rather than just you know, doing cases because something's come and you're not being paid for it, so it's multifactorial. You know, we defend our results." (K4.Surgeon)<br><br>"[Participant:] When you look at rates of resurfacing across the world, there is quite wide variation in what goes on, and actually if you look at the I think the Swedish Knee Arthroplasty Register would be worth looking at. I'm fairly certain that their rates of resurfacing were higher and have gone down over the same period of time.<br>[Researcher:] Uh-huh.<br>[Participant:] So, when I last looked, and this was quite a few years ago, their rate of resurfacing was about less than 10 per cent.<br>[Researcher:] Oh, wow.<br>[Participant:] whereas in the US, you know, the rate of resurfacing is three quarters, 80 per cent, and again some of it comes down to coding, reimbursement, uplifts and how people are paid. So, all of these factors influence the rate of resurfacing, possibly more so than evidence from one RCT." (K3.Surgeon)<br><br>"While [the KAT trial] made a recommendation that patella resurfacing should be done. And to be fair, NICE did the same when we read the guideline recently." (K3.Surgeon)<br><br>"Probably the biggest stimulation [for surgeons] to do that [change from not resurfacing to resurfacing] and whether this plays out or not is the last year's NJR report." (K2.Coding expert/surgeon)<br><br>"Historically as I said, you know, the NJR didn't count for secondary resurfacing as a revision procedure. So, surgeons would do it and, you know, and they would have a complication and they would say, well, why would I bother doing it? If I need to do it I'll just do it later on anyways, it's not a problem. Whereas now they're starting to look at it and say well, if I do it later on it is a problem because it's counting in my revision numbers. So, I think more than the trial, that is what's going to drive practice currently. It would be interesting to look at this in another five, 10 years' time with HES data and see, has the mandation of patella resurfacing as a revision procedure. Has that driven up the rate of resurfacing, because I strongly suspect it will." (K3.Surgeon)<br><br>"The National Joint Registry they have changed the way that they look at patella resurfacing now in terms of coding it as a second revision procedure, whereas that wasn't always the case. So, when you went back in and did a secondary research thing it didn't count against your numbers as a revision operation, whereas now it does. So, people, hopefully you'll see another shift towards secondary resurfacing 'cos people think, if I do it now, nobody can do it later on, and, therefore, it won't reflect poorly on me as a surgeon that I've got a higher revision rate." (K3.Surgeon)<br><br>""So, the National Joint Registry review last year published outcomes with or without a patella which had never been done before, and so that that may influence some people's practice." (K4.Surgeon)<br><br>"I think the commissioning policies are one driver. You're probably interested in how research influences the commissioning policies writing process, and its policy writing process basically tends to be driven by guideline production rather than individual studies. So, we'll use NICE Guidelines. We have sort of a non-treatment advisory group that looks at some of the research, the primary research, and the guidelines, and those come together to form the commissioning policy. So, hopefully, our suite of commissioning policies are evidence-based and research-based rather than purely rationing-based. (General3.General practitioner)<br><br>"NICE has very recently commented on this. And they do quote our work. And, you know, they've made a recommendation based upon it. But probably the most influential organisation is the		√	"The CCG has got to be cost effective and efficient, and try and adhere as best it can to NICE guidel trial evidence, what have you not, does it always 100% happen all the time? Probably not, no. I don' CCG- bear in mind it's not a profit organisation, it's not trying to make a profit at the end of the year, i trying to pay off its shareholders what have you not, it's one and only remit is to pay for medical care constituent, and with an ever-shrinking budget, it just gets harder and harder." (General4.General pra<br><br>"They're [the surgeries we commission are] meant to be value for money, which is very important, so remember back in medical school they were banging on about the cost per QUALY, there's a measu bang for your buck and it's still used today. So when I look at this, I think they are pointing to the righ do, each study [the trials: EVAR, KAT, REFLUX, CLaSS and REACTIV], but from a commissioner's view, it's not just about it's better for patients but that it's also value for money for the commissioning is finite and it's not a bottomless pit, because if we spent say £1 million on one person for something be nice to have say following a large media campaign for them, we wouldn't do that by the way, but unfortunately that would remove funds for other people who could have money spent on them for all that are evidenced based and shown to be good value for money," (General1.General practitioner)<br><br>"When CCG took over the budget, they realised there was a lot of spend that was happening, that wa inappropriate spend on cosmetic processors that weren't adding value to the patients. And what it w rather than individual CCG's trying to come with policies in an inconsistent way, if all the CCG's grou together and came up with an evidence framework that they could all use, then...and all support and then that's what each of the governing bodies and the CCG's would have signed up to, then we coul standardisation into our referrals. Because all CCG's referring to common interest and providers. S want, you know, a lottery for the providers of one not willing to pay for the same type of treatment co through. So, we've got a level of consistency which allows the providers to know when the referrals through that there isn't a risk for them not getting a tariff or payment for that case or that consult. So certainly was very early on in the CCG's. And the basis of the [clinical commissioning] document is based looking at current practice, best outcomes, best treatments for the patients to make sure that providing appropriate care, NICE-level care to our patients."(R.and.CVV6-Other.General practitioner<br><br>"There was a list of low-priority treatments that you ought to look outright and find somewhere, which produce, oh, there was a lot of argy-bargy about it, about three or four years ago, and varicose veins And that also, you see, will have been influential." (RandCVV2.Author)<br><br>"We've also seen the changes in the way the health economics is run to be payment by result, paym episode, and a realisation that, actually, from GPs of course, that many of these people who were ge veins done actually were not having great outcomes in the long run. Because, actually, the early par the patient selection for the procedure." (General3.General practitioner)<br><br>""There has been policy development within commissioning policy for the veins as well. So, we've de commissioning policy that, first of all, said, "We don't do varicose veins for cosmetic reasons, all righ don't do varicose vein surgery at all." Then said that, over the evolution of the time that I've been inv said, "We do the varicose vein surgery, but it has to be one of these newer techniques rather than lig stripping. And it has to be for specific indications like pain, ulceration, bleeding rather than for cosme reasons." So, that's where the policy now is on veins in the [REDACT LOCATION]. So, the Commis Policy now after several iterations is that we do varicose vein, endovascular laser, foam scleротherap it is for varicose veins with significant medical complications." (General3.General practitioner)<br><br>"General practice or something like, yeah, we'll refer some of these people. So, [the commissioners a influencing them at that point, and also potentially influencing the surgeons who are seeing the peop selection and saying, "Well, actually, no, I'm not going to do a ligation stripping here because my hos managers will come down on me like a ton of bricks, and because I'm not going to be the reason the cost weren't all paid. And we also put in place sort of an audit trail, a tick box to make sure that we d or not pay in certain circumstances." (General3.General practitioner)<br><br>""I think the commissioning policies are one driver. You're probably interested in how research influe commissioning policies writing process, and its policy writing process basically tends to be driven by production rather than individual studies. So, we'll use NICE Guidelines. We have sort of a non-treat advisory group that looks at some of the research, the primary research, and the guidelines, and tho together to form the commissioning policy. So, hopefully, our suite of commissioning policies are evi based and research-based rather than purely rationing-based. (General3.General practitioner)<br><br>"I'm happy to talk about the funding issues around varicose veins. I suspect the plummet in the num procedures done is because of the restriction of funding in the NHS over the last, kind of, 18 years." (General2.General practitioner)<br><br>"Fundamentally, decisions in the NHS are often not made on cost-effectiveness grounds. You know, makes decisions about what it recommends, it often will make cost-effectiveness decisions. But whe purchasers decide what they're going to buy, and they're short of money, then they cut the things the rather than cut the things that are not cost-effective." (R.VV1.Author)<br><br>"Varicose veins is something called a threshold-dependent procedure. And so you have to reach a c threshold to justify treatment. And that threshold is actually pretty high. It's, kind of, severe symptom time you're getting venous ulcers, you're talking quite extensive varicose veins. … Over time, you kn funding constrictions get tighter and tighter, the threshold for doing procedures gets higher and highe (General2.General practitioner)<br><br>"A lot of surgeons feel that they're being stopped from doing stuff by funding restrictions imposed by we do really not have anything - any influence over. I suppose a handful of GPs do, but even then, y

BMJ Publishing Group Limited (BMJ) disclaims all liability and responsibility arising from any reliance placed on this supplemental material which has been supplied by the author(s)

							group that looks at some of the research, the primary research, and the guidelines, and those come together to form the commissioning policy. So, hopefully, our suite of commissioning policies are evidence-based and research-based rather than purely rationing-based. (General3.General practitioner)<br><br>"I think this study [about EVAR and Open surgery] is most closely linked to the value based clinical commissioning policy work that I do, it really fits with that, and the reason for that is that the evidence base changes over time, and that the NHS has a responsibility to commission procedures, interventions, any treatment actually that could be pharmaceutical to medications, it could be cognitive therapies. But anyway, they are meant to have an evidence base." (General1.General Practitioner)						our policy in the latest literation in April 2020 has a set criteria that must be achieved for referral. An will only receive tariff payments if those criteria were met." (R.and.CVV6-Other.General practitioner)<br><br>[Practitioner explaining their clinical commissioned pathway]<br>""In the [location redacted] region asked for endothermal ablation and endovenous laser treatment a be first lined. And if those aren't suitable, ultra-sound guided foam sclerotherapy. And if that's not su surgery is third line. And non-intervention treatments are not recommended unless intervention treat unsuitable for clinical reasons or for patient choice."(R.and.CVV6-Other.General practitioner)<br><br>"We're [commissioners are] not denying any treatments that patients should have access to. But eq not...the NHS has prior to these types of policies, has provided above and beyond what it set out to patients. There was an awful lot of relative cosmetic treatments that were going ahead. And that wa the major changes that had to happen when CCG's were left with historical debt effectively from PCT (R.and.CC6.General practitioner)<br><br>"NICE also bases some of its decisions on cost-effectiveness, and clinicians aren't in general interes effectiveness. They're interested in clinical effectiveness. So, if you're faced with a patient, you wan most effective treatment on them, not the most cost-effective treatment. So, unless NICE actually pu teeth into something as it does with appraisals and says you can't use this because it's too expensiv clinicians think that's something better, they will use it even if it is more expensive." (R.VV1.Author)<br><br>"The commissioners started demand management, and they started creating all these referral hurdle referral guidelines saying, "You shouldn't be treating cosmetic varicose veins. You should only be tr if there are leg ulcers or skin changes," and so on. So, you'll find that around the early 2000s, a lot o was it PCT or CCGs? And I think it was PCTs at that time, started producing referral guidelines for v veins that said, "Don't refer patients unless they've got skin changes or leg ulcers." So, the cosmetic veins were not being referred. And that significantly reduced the number of patients with relatively m varicose veins that were being referred." (R.VV1.Author)<br><br>"The commissioning restrictions that were introduced more or less following the financial crash in 20 restrictions were introduced 2009/10, et cetera by huge parts of the country in varicose vein treatme you aware of that? (RandCVV2.Author)<br><br>"What happened about 2009, 2010, people were very short of money. And throughout a lot of the co commissioners suddenly started restricting varicose vein treatments." (RandCVV2.Author)<br><br>"We, in this locality, produced guidelines which still exist, which say that you are not allowed to be re NHS treatment if you simply have veins that don't look very nice. If you only got symptoms, which I t completely wrong and unfair, but you could only be referred if you have eczema, skin damage, bleed phlebitis, which we haven't dealt with. And so, those are restrictive guidance."(RandCVV2.Author)<br><br>"I would influence NICE and suggested they produce a guideline, which they did in 2013, suggesting actually, the recommendation was that anyone with troublesome symptoms should be referred, and obviously with skin damage and bleeding and things certainly should be referred urgently. And so, th most, to me, the most powerful influence for the NHS about what they ought to be doing. And that N guideline was produced in 2013 largely in response to what had happened in terms of the increasing in treatment during the prior three or four years." (RandCVV2.Author)<br><br>"The NICE guidance by 2013 had become a hugely influential input into NHS practice, you know, ver more so than many individual trials. So, I think you shouldn't underestimate the expected impact of th guidance." (RandCVV2.Author)<br><br>"[NICE technology Appraisal guidance] comes with a funding mandate. Whereas NICE clinical guide not, they have no mandate to them. They are simply recommendations. And, you know, the fact to th was that the NICE guidelines, clinical guidelines on varicose veins in 2013, came into a system wher commissioners had made up their minds that they wanted to reduce funding, and they couldn't see a reason to reinstate it [varicose vein surgery]." (RandCVV2.Author)<br><br>"[participant:] There are two main things about the [2013] NICE guideline. One was the ranking of di treatments that it recommended, which as you know were, number one, endothermal ablation; numb foam; number three, surgery, in that order very explicitly. But number two was the fact that and that the most important thing from your point of view.<br>[researcher:] Yeah.<br>[participant:] But the subtext is that the NICE guidelines said anyone with troublesome symptoms sh referred and treated. And anyone this, that, and the other should be referred and treated. But actua commissioners had already made up their minds. That actually, people with some symptoms due to varicose veins simply were lower priority than a lot of other things that were going on. And in fact, o things that's hacked vascular surgeons off is that varicose veins had been listed in NHS documents priority treatment." (RandCVV2.Author)<br><br>"Most surgeons, if they get referred a patient will offer what they consider to be the best treatments. And that may present depending on presenting features of the patient and also what the patient's wi and precede preconceptions. But if you don't get referred the patients to start off with, you're not able them what is a cost-effective therapy which improves their quality of life. And that's because not all p referred by their primary care doctors into secondary care, there were certain restrictions either put o primary care trusts and have their set up or within secondary care as to what referrals they will acce (CVV1.Author)<br><br>"You will notice that not many people can be referred these days on the NHS, certainly nothing cosm patients might say] "I don't like the look of my varicose vein, look at the state of them in my shorts". I matter, we're not operating for that, you have to get that done privately sir." (General1.General pract<br><br>"But this reflects the state of the NHS. Even though there's evidence that patients with C2 varicose v from surgery [as seen in the REATIV trial], you know, there's clearly clinical effectiveness and cost e in treating C2 varicose veins, you know, there have been lots of guidelines published by the NHS ar The guidelines from the government have restricted who we can offer varicose veins treatments to a restricted it to the higher end of the C classifications. So C6 and 5, so active ulcers or healed ulcers recently C4, so complicated varicose veins. At this time, before the HTA report was published, I wou of interventions on varicose veins were for C2 varicose veins. And if you'd take away the funding to those procedures, those procedures won't get done. So this reflects a change in government policy, effectively stopped us operating on C2 varicose veins. (RandCVV3.Surgeon)<br><br>"The main struggle for the REACTIV trial was against funding, varicose veins are perceived by comm as an unimportant condition although the reactive trial providing good evidence of cost-effective bene the commissioners have ignored that and also ignored the NICE recommendations." (R.and.CVV5.S<br><br>"Some of the decline [in surgeries] from the in the surgical is replacement by other treatments which increased in number. But I think some of the declines is also an increase in pressure on funding, ba in terms of the REACTIV trial, I think the REACTIV trial has largely been ignored by the commissione it's believed by the clinicians but not by the commissioners." (RandVV5.Surgeon)
**3. Inner Setting**													
**3.A. Structural Characteristics** – The social architecture, age, maturity, and size of an organization.	"I think a lot of that may be looking at pathways, care pathways within the stroke area. I think also at that point [points to near 2005], stroke per se certainly in this trust as a real speciality was not as high profile as maybe as it does now. So, certainly here, stroke patients were cared more for within acute medicine or within elderly care rather than as a stroke speciality. And it was only sort of after this time that stroke as a speciality, certainly in this area, became more recognised. So, whether getting those care pathways in place took some time before that was taken up is possibly what I can say on that, yes." (F3.Other-Dietician)<br><br>"[participant:] I think that probably would be the only other thing I'd like to add there is the pathways. Yes.<br>[researcher:] Yeah, I suppose not all hospitals have a clear stroke pathway yet.<br>[participant:] No, no. Maybe pathways not the right word there, but facility available for it." (F3.Other-Dietician)		√	"To me, I think if you were to say to people how would you do this or would you know about other and certainly the younger GPs that are coming through, my trainees as such, I don't think they know about it as much either, but again that's probably just because they haven't had the experience of patients going through the system." (General4.General practitioner)	X		"Now part of the reason for mentioning all that developmental warfare, et cetera, is it took a long time for this to really settle down, and you know, by say 10 years ago, everywhere had settled down and they got very good systems for doing EVAR." (E1.Surgeon)<br><br>"One of the things is that UK EVAR trial 1 was one of the very early ones that started recruitment in 1998, 1999, when the expertise was less planned, it was not good, understanding of the importance of instructions for us and how to get the most out of endovascular repair is not that understood. The technology was also in fashion. So due to both improvements in technology and improvements in understanding the physicians as to how to get better results out of technology meant that there were some question mark if that those results would be applicable to abide to today's practice." (E2.Surgeon)<br><br>"The graphs are 15-year-old historically, their historical graphs, and everything so much better and we're all so much better now that the results no longer apply. And that's the problem with any long-term data for many studies." (E3.Radiologist)<br><br>"Initially it was regarded as you know, pushing the boundaries to be doing these by EVAR. Now according to NICE guidelines and everyone's experience, if you're all set up, you're much better to have an EVAR for a rupture than you are an open repair, and so the point that I'm making to you is it's taken a very long time and lots of evidence for everywhere to get set up so the way of doing EVAR is all worked out systematically, all the facilities are there, you get patients out of hospital within 48 hours compared with the old fashioned if you like, approach of an open aneurism repair which mean a major operation, an operating theatre, going to intensive care depending how ill you are for 24 hours or more, and being in hospital for a number of days, so you got a situation which is developed by the 2000-and-teens, where EVAR had become sort of the dominant thing to do." (E1.Surgeon)<br><br>"I would not wish to face the threat of the kind of complications that an open procedure would give me, and so what- with that and the whole system being set up to do EVAR and looking around the world, that's what everybody else does, the idea suddenly moving away from that was both practically, philosophically, in terms of counselling patients, difficult, and added to that you know, if we'd suddenly said let's start doing a lot more open operations, suddenly you've got the whole issues with your Trust, hang on, you know, we need more intensive care beds which are very difficult, they're dealing with COVID, and so you know, the whole business of doing an open repair has become a lot more unusual and a lot more involved, and so with all of that, I hope I painted a picture that to start to move back from this business of having organised to do EVARs for your ruptures if you can, you've got the whole system ready for doing EVAR and you get patients out of hospital quicker and its less invasive, which patients like because there the whole thing sounds better, why would you move back to open because of this a bit of evidence that a few patients are going to do less for long term." (E1.Surgeon)	X		"I was lucky enough to get my revision scores and they're two standard deviations above the mean, so I do the patella. Is that because I do the patellar, or is it because I use the implant? It has probably nothing to do with me, and it's just the fact that we've got a good system, and that we know we got, you know, good physios, good rehab people, and...and people who seem to do well." (K4.Surgeon)		√	
**3.B. Networks & Communications** – *The nature and quality of webs of social networks and the nature and quality of formal and informal communications within an organization.*	"Another secular trend that's been going on in the background since then [when the FOOD trial was published] is the introduction of hospital nutrition teams." (F2.Surgeon)		√	""I guess what I see my role in is being that halfway house between the definitive surgical and the procedure on the surgeon and nothing, and I can advise what best to do." (General4.General practitioner)<br><br>"We would refer probably half a percent of everybody we treat for reflux, okay. Our job is to be gatekeepers. If we referred everybody, what would be the point of general practice, there would be no point at all." (General1.General practitioner)<br><br>"If I referred everybody with bad reflux at the first consultation, the hospital would fall over." (General4.General practitioner)<br><br>"I suspect it's primarily because, you know, primary care aren't referring them, essentially, or GPs aren't referring them in." (Ref2.Other-Gastroentologist)<br><br>"I suspect the threshold for GP's referral is probably something along the lines of, you know, if you have a normal weight, you don't smoke, you don't drink caffeine, you don't drink alcohol, you have a reasonable diet, and you're still getting reflux. Yes, I'll refer you.' But up to that point, I suspect they are probably likely to say, you know, there are other things you can do before we do this. And I suspect the reason for that." (General2.General practitioner)<br><br>"I think those numbers are very small. I think primarily because we're just not seeing them and the house data, you know, looking at admissions to hospital, you know, for GORD there's, you know, a few and far between, aren't they?" (Ref2.Other-Gastroentologist)<br><br>"A lot of surgeons feel that they're being stopped from doing stuff by funding restrictions imposed by GPs, which we do really not have anything - any influence over. I suppose a handful of GPs do, but even then, you're operating within very strict guidelines from the finance departments and the Department of Health. And, ultimately, once something isn't funded, it's very unlikely it's going to be funded. You know, it's more about taking things that are funded and funding them, rather than things that aren't funded and then funding them, so." (General2.General practitioner)	X	√				"[participant:] Once you get to that five-to-10-year point [treating a patient with knee pain], you've spoken to the patients and said, "Look, I as your GP have nothing more to offer you. I've injected your knee two or three times; I've sent you to the physio. I've given you some pretty strong pain relief. We're at a point now where I'm happy to offer you a chance to have a conversation with a knee surgeon." Now I know the knee surgeons were doing these uni-compartmental or arthroscopic washouts or this, that and the other. And I know that's becoming massively out of favour in recent years as such, so. And the only surgery I believe they should be doing as for the trial evidence is the knee replacements or not but once I've written my referral letter, I'm sorry to say, that's the conversation to be had between the surgeon and the patient. Yeah, I don't know.<br>[researcher:] But there's a clear pathway, it sounds like, for you to follow.<br>[participant:] Yeah." (General4.General practitioner)<br><br>""I guess what I see my role in is being that halfway house between the definitive surgical and the procedure on the surgeon and nothing, and I can advise what best to do." (General4.General practitioner)		√	"When it comes to getting advice from the hospitals, we [the general practitioners] currently have ac either direct contact so we can ring consultants or registrars about conditions. We have been able to some time into hospital settings through, and that more recently has been E-referral." (RandCVV6.O General practitioner)<br><br>"Most surgeons, if they get referred a patient will offer what they consider to be the best treatments. And that may present depending on presenting features of the patient and also what the patient's wi and precede preconceptions. But if you don't get referred the patients to start off with, you're not able them what is a cost-effective therapy which improves their quality of life. And that's because not all p referred by their primary care doctors into secondary care, there were certain restrictions either put o primary care trusts and have their set up or within secondary care as to what referrals they will acce (CVV1.Author)
				"I think we're [the surgeons are] sort of dependent on our referral pathways which often will come either through gastroenterology or direct from GPs. And then once they are referred to us, normally that's people that are already a bit or at least partially aware of what anti reflux surgery involves. And a lot of the patients we see if they're diagnosed with pathological reflux, we'll proceed with surgery in general." (Ref1.Surgeon)									

BMJ Publishing Group Limited (BMJ) disclaims all liability and responsibility arising from any reliance placed on this supplemental material which has been supplied by the author(s)

				"I think it all depends on centres to be honest. And I would assume all of these are very variable between the different hospitals depending on how many surgeons you've got, how interested they are in anti-reflux surgery, and how much collaboration there is between the gastroenterologists and the surgeons. In our centre, all of those are quite good. So, I would assume in our hospital here, we tend to get more patients on surgery maybe. That is something that I would assume because our surgeons are fairly interested in this kind of surgery is my understanding." (Ref3.Other.Gastroentologist)								
**3.C. Culture** – *Norms, values, and basic assumptions of a given organization.*	"The result of the third FOOD trial was somewhat unexpected, given that we did the trial, that particular arm of the trial, backup following the result of the Norton, et al. paper in the BMJ. I think it was, which randomised 32 patients and found a 70% reduction in death related to use of PEG. So that had caused in the years before we did the FOOD trial an upsurge, we felt, in enthusiasm for PEG, which really wasn't justified by the evidence because the trial was not done well and didn't report important function outcomes. So that particular FOOD trial, FOOD three, was dominant in an era where there was an enthusiasm for PEG caused by very small and not very robust studies and quite a lot of observational work." (F1.Author)<br><br>"Culturally there's been a big change in attitudes towards managing nutrition and hydration. So, since the GMC brought out their guidelines about meeting nutritional needs and avoiding prolonging intolerable life, is that phrase that they used, the dieticians and myself at least, we use that quite a lot and try and get doctors to really think about the appropriacy of non-oral feeding." (F4.Other.Speech and Language Therapist)<br><br>"Historically, you're right. The process for fitting a PEG, fitting a stomach tube, would've been just the relationship between the stroke physician and their referring gastroenterologist who would be responsible just for the procedure themselves. And so, then they were just a proceduralist. You know, just do the tube, don't ask any questions. What happens now with hospital nutrition teams is usually their multidisciplinary teams, including a nutrition nurse, a dietician, and the gastroenterologist responsible for the procedure itself. And the involvement of a nutrition team means that issues around the best interests and the ethics of fitting a PEG tube are addressed much more rigorously nowadays." (F2.Surgeon)<br><br>"The other major secular trend that's been going on in the background, behind your graph, is the development, firstly, of hospital nutrition teams, who take a much greater interest in the rights and wrongs of a PEG being fitted." (F2.Surgeon)<br><br>""Families are against these kind of surgical interventions, purely for the purpose of prolonging life. Maybe that reflects - I mean, it's hard to tell when you're a medic because you have a, sort of, biased sample. But maybe that reflects popular discourse about the rights and wrongs of living with severe disability, you know. Nowadays, talk about people with motor neurone disease going to Switzerland to end their lives, etcetera, etcetera. The issue of what quality of life you might be like living with severe disability is much more out in the open, I think. And I think that means that patients and their families have more of a view about what's in their best interest of their loved one. And, of course, in a good way, medicine has become much more aware of its responsibilities to establish the needs and wants of patients in relation to severe disability. That might've been the case in the, sort of, paternalistic era in the past. And I think that opening up of debate about whether or not this is really something that your mother would've wanted. Does she want us to put her through a surgical procedure in order that she can have a permanent plastic feeding tube into her stomach so that she can be kept artificially alive? Or should we actually be letting nature take its course? And I think that sort of conversation occurs more frequently now than it used to in the past." (-F2.Surgeon)<br><br>"It's probably been at least a couple of years since I've actually referred one of my stroke patients for a PEG. And I was thinking why on earth is that? Is it something that I should or shouldn't be doing? And I came to the conclusion that it is because we are much more explicit now with families about the value of surviving with severe disability and ensuring that we've established the patient's wishes to a much greater extent than we did in the past. So, it's interesting, isn't it? Because that wasn't one of the original hypotheses that the FOOD trial was testing. But it's proved to be part of a landscape which has prompted us to think in more detail about what it means to survive with a severe disability." (F2.Surgeon)<br><br>"The Royal College of Physicians and also the Royal College of Speech and Language Therapists have produced probably, again, over the last, sort of, six or eight years, more practice guidance about this notion of feeding at risk, which isn't something that we would've been doing in the time that the food trial was being performed. Which is the idea of saying to a patient or their family, "Well, actually, we're not going to place a feeding tube. Instead, even though you've got swallowing difficulties after your stroke, doesn't look like they're going to be permanent. We're going to let you have food and drink that you want as part of our whole package of palliative care for someone with severe disability after stroke." And that scenario of practice that's sort of come in tandem with the demise of the PEG, and I expect those two are related. We're more willing now to palliate people after stroke with feeding at risk when, before, the only measure that we had, rightly or wrongly, was to stick in a PEG." (F2.Surgeon)<br><br>"The context of the evidence when the FOOD trial was originally drawn up was small-scale studies suggesting benefits to patients from the early placement of a PEG tube. And so that was the context in which FOOD trial occurred, but the FOOD trial convinced clinicians that that wasn't necessary, and they should wait and see if people are still chronically dysphagic or unable to swallow after a major stroke." (F2.Surgeon)<br><br>"There's a lot of nursing literature, which was very much pushing against any form of restraint [being] seen as unethical. And I think, hopefully, we now have a more balanced view, that you've got to take a holistic view of what you're trying to achieve. And it isn't always unethical to restrain in some way to achieve the better adherence to a given intervention." (F1.Author)		√	"I don't know if it's evidence-based that the patients usually get tried on a PPI for some time, at least a few months before we even consider surgery, speaking as a non-surgeon obviously."(Ref3.Other-Gastroentologist)			"Initially it was regarded as you know, pushing the boundaries to be doing these by EVAR. Now according to NICE guidelines and everyone's experience, if you're all set up, you're much better to have an EVAR for a rupture than you are an open repair, and so the point that I'm making to you is it's taken a very long time and lots of evidence for everywhere to get set up so the way of doing EVAR is all worked out systematically, all the facilities are there, you get patients out of hospital within 48 hours compared with the old fashioned if you like, approach of an open aneurism repair which mean a major operation, an operating theatre, going to intensive care depending how ill you are for 24 hours or more, and being in hospital for a number of days, so you got a situation which is developed by the 2000-and-teens, where EVAR had become sort of the dominant thing to do." (E1.Surgeon)<br><br>"It is important to distinguish that from the importance of measuring all-cause mortality because without that we cannot actually establish the value of the treatment in actually achieving one of the aims and also very important for United Kingdom, less important for United States et cetera which is the health economic analysis and the willingness to pay in the publically funded healthcare systems. So if you are- if you are measuring the efficacy of endovascular repair which is a disease specific treatment in terms of its ability to stop the patient from coming to harm from that particular disease, all-cause mortality has really no value because it is only specific mortality you should look for. However, to our view, that I am not going to look at all-cause mortality is untenable that is because the only reason why you repair an aneurysm is to stop a premature death and death from however method it may occur has a relevance to the utility of your operation in the overall aim." (E2.Surgeon)	X	√	""I think that surgeons by their nature are a creature of habit in their training. So you may find which you could probably dig down to in some way, shape, or form within the HES data is there may well be quite significant geographic variations within the UK with rates of resurfacing. Certainly, when I was training in the [location redacted], I would say, I worked for a number of different consultants, and I saw many of them resurfacing the patella. And consequently, when I started as a consultant, my rate of patella resurfacing was probably about 10%, 5%, very little. And it's gone up in recent years because I've seen some problems related to not resurfacing the patella, so I was selective in resurfacing in a group of patients. But I'm probably still only at about 30%. I think there's very few people that I know that resurface the kneecap in everybody. But you might find that's different in other regions of the country. So, I think it's a product of what you see, what you do, and how you've been trained." (K3.Surgeon)<br><br>"You'll get some surgeons who don't resurface but you got a patient with rheumatoid arthritis will always resurface it because that's what you do." (K2.Coding expert/surgeon)	X	√	"There has been a sort of impression among many of those running the NHS and payers that varicos [illegible] sort of subtly unimportant that ladies have done for cosmetic reasons, which is completely incorrect. [illegible] remains a persistent sort of impression among many people who aren't in the specialty of making str [illegible] decisions." (R.and.C.VV2.Author)
**3.D. Implementation Climate** – *The absorptive capacity for change, shared receptivity of involved individuals to an intervention, and the extent to which use of that intervention will be rewarded, supported, and expected within their organization.*							"The 30-day results which certainly, as you've alluded to, allowed EVAR to continue. We always knew that if the early results were poorer then EVAR was sort of dead in the water because we knew there were long-term penalties to pay for endovascular repair. So, this 30-day results allowed EVAR to continue, the 10 to 15-year results occurred at a time when there were additional reasons for people to dispute the findings if they didn't want to hear them." (E3.Other-Radiologist)<br><br>"I never heard a trial more picked apart than EVAR II and its results. And I think the reason for that, you know, is people wanted to carry as they were doing." (E3.Other-Radiologist)	X					
**3.D1. Tension for Change** – *The degree to which stakeholders perceive the current situation as intolerable or needing change.*							"The other thing is you know, having- to introduce EVAR all those years ago and to get systems for doing it you know, they've [health service managers] been through a whole lot of financial wrangling with their trusts anyway. To retrench on all that just, you know, I can see no incentive for the average UK surgeon. (E1.Surgeon)	X					
**3.D2. Compatibility** – *The degree of tangible fit between meaning and values attached to the intervention by involved individuals, how those align with individuals' own norms, values, and perceived risks and needs, and how the intervention fits with existing workflows and systems.*	"There's also a lot of external influencing factors, so back in the day I can remember that obviously people would go home with an NG if they were in nursing homes maybe sometimes in their own home as well, but they could go to a nursing home with an NG, and they can't do that now. So, unless the patient has a PEG they have to stay in hospital, and so for some people we don't have any choice but to put a PEG, place a PEG." (F4.Other.Speech and Language Therapist)	X		"Within a surgical sphere, with the two big studies, there are smaller ones available where LOTUS and REFLUX were the two big ones that we're all aware of. And I think that's probably what we used to support our practice. I don't know whether actually they- I would say they're more in a position to support our practice rather than necessarily change practice is the thing, so I would say this, this [the reflux trial] is more a study that supports what we were doing already in that the majority of patients that were referred for to us who want anti reflux surgery would have it." (Ref1.Surgeon)	X		""Soon after that [the EVAR 15-year outcomes were published], several, a few months after, that came a consultation document of NICE guidelines would try to enforce that [the results of the 15-year follow up] into or put that, incorporate that, into clinical guidelines which created quite a lot of controversy and physicians got extremely excided about it. And that resulted in a lot of debate and representations to the NICE saying that that recommendation is actually it is not possible to implement it for various different reasons to the extent that one of the rare things to happen was the NICE have actually amended the guidelines to the extent that practically they lost their teeth." (E2.Surgeon)	X	√	"Knee replacement and the hip replacement are famously known as some of the best cost for quality, cost per QUALY procedures around and they have been for decades, so we understand that. And I guess the resurfacing of the patella just adds a bit of a detail to that and just makes a knee replacement even better, it's already a good operation if it's done on the right patients." (General1.General practitioner)		√	
**3.D3. Relative Priority** – *Individuals' shared perception of the importance of the implementation within the organization.*	"The other major secular trend that's been going on in the background, behind your graph, is the development, firstly, of hospital nutrition teams, who take a much greater interest in the rights and wrongs of a PEG being fitted." (F2.Surgeon)<br><br>"The Royal College of Physicians and also the Royal College of Speech and Language Therapists have produced probably, again, over the last, sort of, six or eight years, more practice guidance about this notion of feeding at risk, which isn't something that we would've been doing in the time that the food trial was being performed. Which is the idea of saying to a patient or their family, "Well, actually, we're not going to place a feeding tube. Instead, even though you've got swallowing difficulties after your stroke, doesn't look like they're going to be permanent. We're going to let you have food and drink that you want as part of our whole package of palliative care for someone with severe disability after stroke." And that scenario of practice that's sort of come in tandem with the demise of the PEG, and I expect those two are related. We're more willing now to palliate people after stroke with feeding at risk when, before, the only measure that we had, rightly or wrongly, was to stick in a PEG." (F2.Surgeon)		√	"The CCG for years has been tightening and tightening and tightening their belt buckets. … I can't off at the top of my head that we've got a GORD pathway, and in many ways it's kind of try medical management to the patients effectively keeling over in front of you with reflux then refer them in. I mean, you've got to be careful of all your red flags and you don't want to miss a cancer or the like but assuming that's all been ruled out, it's not within the CCG's radar and broadcast message to say you should be referring them up as such." (General4.General practitioner)	X								
**3.D4. Organizational Incentives & Rewards** – *Extrinsic incentives such as goal-sharing awards, performance reviews, promotions, and raises in salary, and less tangible incentives such as increased stature or respect.*							"Endovascular repair took off greater than anything else for a combination of reasons. Patients get quicker better, they like it. Hospital beds are becoming fewer in number, and critical care beds are becoming fewer in number and difficult to get, these are quite...operations from which compared to open surgery you can send them quicker. But very importantly UK has also adopted individual surgeon levelled result reporting." (E2.Surgeon)<br><br>"At the very early years of your graph there, there are Europe wide audits that showed UK to be the worst performer in terms of 30-day mortality after elective aneurysm repair because 10 to 12% mortality they're worse in the United Kingdom than Europe. So there is not only a national quality improvement programme who's aim primarily is to reduce 30-day mortality rate, there is also a heightened impetus among surgeons to do whatever they can to reduce the 30-day mortality by whatever technique. Sometimes actually change in the technique from endovascular to, sorry, from open to endovascular is a very good thing." (E2.Surgeon)<br><br>"Also the attitude now changes on the bias towards what is modern and all the combinations including the fact that individual surgeon's mortality rate will inevitably look better if you substitute open surgery for endovascular repair. And the UK EVAR trial 1 did not show anything that purely ran counter to that choice, arguably it supported that choice if you take away certain aspects of if, and that perfectly explains the steep trajectory upwards." (E2.Surgeon)	X					
**3.D5. Goals & Feedback** - *The degree to which goals are clearly communicated, acted upon, and fed back to staff, and alignment of that feedback with goals.*													
**3.D6. Learning Climate** – *A climate in which: a) leaders express their own fallibility and need for team members' assistance and input; b) team members feel that they are essential, valued, and knowledgeable partners in the change process; c) individuals feel psychologically safe to try new methods; and d) there is sufficient time and space for reflective thinking and evaluation.*													
**3.E. Readiness of Implementation** - *Tangible and immediate indicators of organizational commitment to its decision to implement an*												"I remember my chief influence in this area were colleagues who were keen to undertake it, rather th [illegible] organizations per se. I think that it was, you know, it was primarily my former colleagues who adopte [illegible] participated in both trials I mean, we participated in both trials. I mean we participated in both REAC [illegible] [illegible] trials with a view to, you know, with a view to developing this and understanding where it's at [illegible] practice. So I think, you know, that's my chief influence rather than outside organizations." (RandCVV5.Surgeon)

BMJ Publishing Group Limited (BMJ) disclaims all liability and responsibility arising from any reliance placed on this supplemental material which has been supplied by the author(s)

	"We can use bridles. We don't use them very much. There are certain criteria, things like, people shouldn't be actively pulling them out. So, if they are actively pulling at them, obviously they're gonna do themselves quite a lot of damage. So, it's more for when they are misplaced and knocked out. But I think maybe when the bridling first came in we probably used it a lot more than we do now. It's with experience that we're more selective about the patients we request it for. We also use mittens. So, we have a whole ream of things we have to go through obviously because it's classed as a form of restraint." (F4.Other.Speech and Language Therapist)<br><br>"I think what we tend to do more now than we certainly did back in 2005, 2006, is we will use bridles to hold NG tubes in place." (F4.Other.Speech and Language Therapist)<br><br>"We put mittens on patients' hands for those who are obviously lacking capacity following assessment. And we fill in a deprivation of liberty. Obviously, paperwork. But we actually put mittens on them to stop them pulling out the NG tubes." (F4.Other.Speech and Language Therapist)<br><br>"The National Patient Safety Agency said that the bedside systems were not accurate enough, and they recommended X-rays, and that made it, therefore, difficult to implement longer-term nasogastric tubes, say in the community where you haven't got easy access to x-rays and NG tubes, etc cetera. So, you know, there's quite a lot of complication around it. Other things coming in, which make NG tube feeding problematic." (F1.Author)						physicians as to how to get better results out of technology meant that there were some question mark if that those results would be applicable to abide to today's practice." (E1.Surgeon)<br><br>"Initially it was regarded as you know, pushing the boundaries to be doing these by EVAR. Now according to NICE guidelines and everyone's experience, if you're all set up, you're much better to have an EVAR for a rupture than you are an open repair, and so the point that I'm making to you is it's taken a very long time and lots of evidence for everywhere to get set up so the way of doing EVAR is all worked out systematically, all the facilities are there, you get patients out of hospital within 48 hours compared with the old fashioned if you like, approach of an open aneurism repair which mean a major operation, an operating theatre, going to intensive care depending how ill you are for 24 hours or more, and being in hospital for a number of days, so you got a situation which is developed by the 2000-and-teens, where EVAR had become sort of the dominant thing to do." (E1.Surgeon)<br><br>"I would not wish to face the threat of the kind of complications that an open procedure would give me, and so what- with that and the whole system being set up to do EVAR and looking around the world, that's what everybody else does, the idea suddenly moving away from that was both practically, philosophically, in terms of counselling patients, difficult, and added to that you know, if we'd suddenly said let's start doing a lot more open operations, suddenly you've got the whole issues with your Trust, hang on, you know, we need more intensive care beds which are very difficult, they're dealing with COVID, and so you know, the whole business of doing an open repair has become a lot more unusual and a lot more involved, and so with all of that, I hope I painted a picture that to start to move back from this business of having organised to do EVARs for your ruptures if you can, you've got the whole system ready for doing EVAR and you get patients out of hospital quicker and its less invasive, which patients like because there the whole thing sounds better, why would you move back to open because of this a bit of evidence that a few patients are going to do less for long term." (E1.Surgeon)<br><br>"It's the argument about endovascular aneurysm and a lot of other things where the trials are carried out. But by the time the trials are finished, the technologies moved on, and people are saying, "well, those trial results aren't really relevant because we've got different technology now." So, I think that's often a problem with the sort of relatively technology-based treatment." (RVV1.Author)<br><br>"In the 90s, when people started to introduce these endovascular stent graft which were some the manufacturers were making, some people, for example, the [location redacted] group were actually making their own out of existing bits of graft material, so these all gradually, gradually evolved, and as you would've seen in the EVAR papers, it's continued to evolved with a continuous evolution in the type and low profile and quality of the stent graft." (E1.Surgeon)<br><br>"As I said at the outset, the whole stent graft technology has moved on quite a lot from the time when the EVAR patients were recruited in the late 80s and early 2000s and you know, we're 20 years and more on with the evolution of different stent grafts, and so probably, and I can only say probably or perhaps that your ability and the need for further interventions now would be less." (E1.Surgeon)			but we know that surgeons just operate anyways. (chuckles) There you go. It's better if you do it before them [the surgeons]. (General1.General practitioner)			"A problem with all this research, when you're talking about research that's dealing with an area of te[illegible] that's moving fairly rapidly. The REACTIV trial took I think it was about 10 years ago no, must be eig[illegible] something like that from when we first envisaged the trial and applied for funding to when we got the[illegible] completed the trial, and published the results. So that time span is enough for there to be quite a lo[illegible] development in technology."(RVV1.Author)
**3.E3. Access to Knowledge & Information** – *Ease of access to digestible information and knowledge about the intervention and how to incorporate it into work tasks.*	"Doctors don't like to be seen to be starving patients. The new GMC guidelines about feeding at risk gave us that other option that people didn't just have to be nil by mouth, or non-orally fed. They could also be fed and just accept the risk." (F4.Other.Speech and Language Therapist)<br><br>"The process now for getting a PEG, you know, involves engaging the nutrition team. And one question that they're bound to ask is, you know, have you established that this is in line with the patient's wishes or those of their family?" (F2.Surgeon)<br><br>"Historically, you're right. The process for fitting a PEG, fitting a stomach tube, would've been just the relationship between the stroke physician and their referring gastroenterologist who would be responsible just for the procedure themselves. And so, then they were just a proceduralist. You know, just do the tube, don't ask any questions. What happens now with hospital nutrition teams is usually their multidisciplinary teams, including a nutrition nurse, a dietician, and the gastroenterologist responsible for the procedure itself. And the involvement of a nutrition team means that issues around the best interests and the ethics of fitting a PEG tube are addressed much more rigorously nowadays." (F2.Surgeon)<br><br>"The other major secular trend that's been going on in the background, behind your graph, is the development, firstly, of hospital nutrition teams, who take a much greater interest in the rights and wrongs of a PEG being fitted." (F2.Surgeon)		√	"One of the obstacles we have is trying to get timely PH and manometry done as a big factor for us. So, you know, even pre COVID, our waiting times were probably six months, and again that's a significant length of time." (Ref2.Other-Gastroentologist)<br><br>"To me, I think if you were to say to people how would you do this or would you know about other and certainly the younger GPs that are coming through, my trainees as such, I don't think they know about it as much either, but again that's probably just because they haven't had the experience of patients going through the system." (General4.General practitioner)	X					""So ODEP is an independent group, that I'm actually part of the...sort of part of, that independently reviews combinations of implants. Now that only for knees actually started in about 2016, but basically, what it does do is it evaluates the outcomes and gives knee systems ratings for how good their survivorship is, so three years, five years, seven years, ten years, and up to fifteen years now. So, a very well-performing implant system, you know, whether certain knee, in a certain patella, in a certain insert, may get a 15A star rating, which basically means that, that 95% of them are still in 15 years down the line. And so that's a way that people can, sort of, independently check combinations of implants to see whether or not the patella actually makes a significant impact on it. But then they only really came out since 2016 and looking at your chart that looks when it starts levelling off rather than accelerating. (K4.Surgeon)<br><br>""So the National Joint Registry review last year published outcomes with or without a patella which had never been done before, and so that that may influence some people's practice." (K4.Surgeon)	X	√	
**4. Characteristics of Individual**													
**4.A. Knowledge & Beliefs** – *Individuals' attitudes toward and value placed on the intervention as well as familiarity with facts, truths, and principles related to the intervention.*	"I would anecdotally record that the use of PEGs in stroke has fallen significantly as well. Although it would be a bold researcher who laid claim to it being just as a direct consequence of their single study. And I think, you know, in interpreting what we see on that graph, we'd have to also consider what other, sort of, secular trends were going on in stroke practice over that time as well."(F2.Surgeon)<br><br>"Doctors don't like to be seen to be starving patients. The new GMC guidelines about feeding at risk gave us that other option that people didn't just have to be nil by mouth, or non-orally fed. They could also be fed and just accept the risk." (F4.Other.Speech and Language Therapist)<br><br>"It's probably been at least a couple of years since I've actually referred one of my stroke patients for a PEG. And I was thinking why on earth is that? Is it something that I should or shouldn't be doing? And I came to the conclusion that it is because we are much more explicit now with families about the value of surviving with severe disability and ensuring that we've established the patient's wishes to a much greater extent than we did in the past. So, it's interesting, isn't it? Because that wasn't one of the original hypotheses that the FOOD trial was testing. But it's proved to be part of a landscape which has prompted us to think in more detail about what it means to survive a severe disability." (F2.Surgeon)		√	"I reckon, nine times out of 10, even a combination of Gaviscon and Omeprazole and tell them to behave themselves during the week. If you're going to have a big meal at weekends, fine. And the ones that we struggle with I would say assuming the more experienced GP say, "Well, look, we've tried you on this, we've tried you on that, we're not really winning. Would you consider surgery?" So, I guess if anyone's prompting that conversation, we as GPs are but that's really due to our own experience of having patients go through that process rather than the patient come and say, "Oh, I read in the paper that I can have surgery which will make me secured" if that makes any sense." (General4.General practitioner)<br><br>""I think there is definitely the influence of the centre where you work. In our hospital here, we have some surgeons who are quite interested in anti-reflux surgery. So, they do tend to take on those patients much earlier or much more proactively. I don't know about the other centres. But here, if I refer them, then they get seen under the surgeons at some point, yeah." (Ref3. Other-Gerontologist)<br><br>"The surgeons don't keep seeing these people who have their GORD surgery for years and years and years [and so do not immediately witness longer-term effects]. So, the vascular surgeons don't keep following up people with varicose vein surgery ever, I don't think. No. (Laughter) They deal with them and they discharge them. That's it. Bye-bye. Off you go." (General3.General practitioner)<br><br>"I refer a patient to an upper GI surgeon for anti-reflux surgery, and they're, you know, a super-specialist to that area, patients probably going to get anti-reflux surgery. You know, whereas, if I refer a patient to an upper GI surgeon who, you know, his primary interest is cancer surgery, you know, they might be less enthusiastic, particularly, you know, if it's somebody in their 20s or 30s, where, you know, we're kind of sitting there thinking, well, actually, there is a failure rate, there is a complication rate, you know, do we really want to do it at this point, or not." (Ref2.Other-Gastroentologist)<br><br>"Quite often, you know, I've seen patients and follow up clinic where the juniors have, you know, switch to PPI. And you're kind of sitting there thinking, what's the point of doing all this? You know, they've clearly got awful reflux, you know, they've probably been there and done all this already in primary care; we've just wasted, you know, six months of their time by not having referred them to a surgeon." (Ref2.Gastroentologist)<br><br>"I've spoken to gastric surgeons about, the upper GI lot, … I did ask her about this, and she said that you need to select your patients very, very carefully for anti-reflux surgery because complication rates are high. So your patient needs to be in real bother in terms of how it affects their day to day life. It's all based on how much it's destroying their life to put it mildly because it has to be in order to justify procedure where you do it and then all of a sudden they're complaining they can't swallow properly or they've got dysphagia and all the rest of it, so she's very picky, and she says a wise surgeon selects their patients very carefully for reflux surgery." (General1.General practitioner)<br><br>"Knowing how GPs think, it's big surgery. You know, you're taking it in. You're grabbing a bit of the stomach. Your wrapping it around the stomach. And the complications of that surgery - my perception - is that could be pretty bad. So why would you put yourself through that when you could - I know it isn't where it isn't easier - isn't easy. But, you know, it's safe (General2.General practitioner)<br><br>"It's probably difficult to say [if the REFLUX trial has influenced practice] as a surgeon because it's probably the findings of the study essentially fitted with what we do surgically and by the time people are referred to us whether they've come through gastroenterology or their GP will normally be working them up towards surgery." (Ref1.Surgeon)<br><br>"I've never had a patient come and say, "I've read the reflux trial, I want anti reflux surgery." I've certainly not seen a referral from a GP that says, "Based on the results of the reflux study, this person would like anti reflux surgery." So, I think certainly as surgeons we're all aware of the results this alongside the LOTUS study which happened a similar sort of time but it's how that influences things at the step before. I think gastroenterologists would be aware of it as well." (Ref1.Surgeon)<br><br>"LOTUS and REFLUX were the two big ones [trials] that we're all aware of. And I think that's probably what we used to support our practice. I don't know whether actually they- I would say they're more in a position to support our practice rather than necessarily change practice is the thing, so I would say this, this [the reflux trial] is more a study that supports what we were doing already in that the majority of patients that were referred for to us who want anti reflux surgery would have it." (Ref1.Surgeon)<br><br>"If we didn't think the operation was effective, we probably wouldn't be doing it if that makes any sense at all. But there are patients- but there are patients that you know will do better than others and there's specific things we look for, but generally if we think the patient would potentially benefit then we would usually offer it if they are, yeah, happy to proceed." (Ref1.Surgeon)	X	√	"I never heard a trial more picked apart than EVAR II and it's results. And I think the reason for that, you know, is people wanted to carry as they were doing. They believe what they were doing was right and they didn't want to hear otherwise, and trials that confirm people's biases are never analyzed in-depth and trials that go against people's prejudices are pulled apart in meetings, in like hyenas pulling apart a wildebeest, it's fast and furious and unpleasant to watch, and EVAR II is dissected in such a manner because people didn't want to hear it." (E3.Radiologist)<br><br>""Attitudes have changed, but more so as a consequence of NICE guidelines which repeated more or less what the 10-year study said. If the NICE guidelines and the controversy never spread, what would have happened, it can only be a speculation. I suspect that probably it will all gone completely unnoticed." (E2.Surgeon)<br><br>""2005 June was when the mid-term follow-up results that were published several months after the 30-day mortality results were published. At the time, the EVAR trials showed, the EVAR trial 1 and EVAR trial 2, people interpreted them, physicians interpreted the way they would like and there was no one unanimous and uncontroversial interpretation of the EVAR trials." (E2.Surgeon)<br><br>""EVAR trial 1 showed that your aneurysm related mortality is about 3% less compared to open repair up to five years, and you have obviously few that are fully operating that. That was seen by in endovascular enthusiast as a clear indication of endovascular technology which should be used. The reason for that is poorly understood by surgeons at large. The study also showed that by about 36 months or so there is no statistically significant difference in all-cause mortality between the two techniques. And EVAR costed more money, and that was interpreted by enthusiasts of conventional surgeons as though there is actually no benefit at all in doing endovascular surgery plus it is actually expensive." (E2.Surgeon)<br><br>"The surgeon's main preoccupation is reducing the absolute risk in the perioperative period because perioperative death is the direct reflection of their care that they're providing and it is a very painful event both from the family and from the surgeon, whoever is repairing. And the absolute reduction in mortality is the highest in high risk patients. So endovascular repair is generally intuitively is seen to be a more preferable option for the higher risk patients and even the unfit patients." (E2.Surgeon)<br><br>"You become a surgeon because you want to do something, you want do to some good to your patient, and you want to help them, you want to give them something positive and help. And some patients are quite sanguine about them being unfit and accept the outcome, but a good number of patients and their relatives are very keen that anything can be done. And at the end of the day standard endovascular repair is actually a relatively straightforward set piece operation which can be done fairly safely and patients tend to improve relatively quickly, so there is a tendency to do it." (E2.Surgeon)<br><br>"I know from operating surgeons, they don't like, and it's nice that they don't like it, but they don't like people to die on them. And so, if 5% of your patients or 3% are going to die from open repair in hospital while they're under your care, even though there's a long-term price to pay. I think there's a psychological bias towards the procedure which might store up fairly in the future but doesn't happen on my short-term watch." (E3.Radiologist)<br><br>"I mean, just from a purely human point of view, people don't want patients whom they know and the families then, they don't, you know, understand and they don't want to die under their care. And I think that influences behaviour because you know the vast, vast majority of patients will come in off the EVAR and go along, and their long-term complications 10 or 15 years might well be under another hospital, another country, another physician, and so it's kind of hidden, the penalties are hidden." (E3.Radiologist)<br><br>""It is very easy to confuse that (NICE guideline drafts) with the EVAR trial results because in a sense, what the NICE guidelines did was to take the EVAR trial results as its text rather than being greatly influenced by other peripheral evidence such as it was on quality of life and other issues. So, I just think you need to be aware that that has really polarised people and authored their thinking since all the controversy of the draft NICE guidelines, which occurred quite soon after the 15-year report."(E1.Surgeon)<br><br>""I think that that transition from open surgery to endovascular treatment has been progress, and I'm comfortable with it. I don't think it's been driven excessively by industry. I think it's been driven by clinicians. And I think that, although industry, I'm sure has had a ride on it. I think, as a whole, it's been a good development. And I think that it's the way forward in terms of the treatment of endovascular surgery. (RandCVV5.Surgeon)	X	√	""Surgeons have different views on the subject. I think one view is the surgeon doesn't want to leave an option for any further surgery to the knee, so most surgeons now tend to do it. There are a group that don't. And if you decide not to resurface it at the beginning, you then have, really, to decide not to resurface it later on if the patient has pain. And another thing the KAT seemed to suggest is that, actually, these late resurfacings don't make any difference." (K1.Author)<br><br>""Surgeons care hugely, but the data relating to, as I've said, whether you replace the patella or not is a relatively small part of doing a knee replacement." (K1.Author)<br><br>"Despite the KAT study, there's a lot of other evidence, there's a lot of other randomised control trials been performed in this. So I think taking one study in isolation is, you know, is slightly unusual. So, there are a number of other RCTs from around the world that basically would demonstrate that potentially there is no significant difference whether you resurface the patella or not. So, I think most surgeons would look at the whole weight of evidence rather than just that from one study" (K3.Surgeon)<br><br>"I had my worst complications from resurfacing patellas in my patients. So as an example, and is one, I've had two patella fractures after resurfacing patellas, one of which required fixation, subsequently got infected and it ended up then having to have multiple revision surgeries to clear her infection. She's been left with a defunction of extensor mechanism and a knee that doesn't really work as it potentially should have done after a primary knee replacement. So, there are issues around patella resurfacing and it's not a completely benign procedure. And I think people would look at it and they would say, as there's no significant evidence of a functional benefit, potentially you're doing an unnecessary additional procedure to prevent yourself having to do an extra procedure in a very small subset." (K3.Surgeon)<br><br>"I look at it as a kind of like a number needed to treat effect. If I did resurfacing on everybody or I did it on nobody, they'd end up with the same functional level probably at the end." (K3.Surgeon)<br><br>"The National Joint Registry they have changed the way that they look at patella resurfacing now in terms of coding it as a second revision procedure, whereas that wasn't always the case. So, when you went back in and did a secondary research thing it didn't count against your numbers as a revision operation, whereas now it does. So, people, hopefully you'll see another shift towards secondary resurfacing 'cos people think, if I do it now, nobody can do it later on, and, therefore, it won't reflect poorly on me as a surgeon that I've got a higher revision rate." (K3.Surgeon)<br><br>""I think that surgeons by their nature are a creature of habit in their training. So you may find which you could probably dig down to in some way, shape, or form within the HES data is there may well be quite significant geographic variations within the UK with rates of resurfacing. Certainly, when I was training in the [location redacted], I would say, I worked for a number of different consultants, and I saw many of them resurfacing the patella. And consequently, when I started as a consultant, my rate of patella resurfacing was probably about 10%, 5%, very little. And it's gone up in recent years because I've seen some problems related to not resurfacing the patella, so I was selective in resurfacing in a group of patients. But I'm probably still only at about 30%. I think there's very few people that I know that resurface the kneecap in everybody. But you might find that's different in other regions of the country. So, I think it's a product of what you see, what you do, and how you've been trained." (K3.Surgeon)<br><br>"While it [the KAT trial] made a recommendation that patella resurfacing should be done. And to be fair, NICE did the same when we read the guideline recently, [anon] and I were involved. You know, the evidence is really around the cost. The recommendation stems from the cost-effective analysis and the cost of secondary surgery. So, I think surgeons put different weight on that information than they do on satisfaction, functional outcomes, and other metrics. And maybe one of the reasons it hasn't been an overwhelming uptake of patella resurfacing is because surgeons on the ground do have these anecdotal experiences where they've had a complication from doing it and it's, you know, it's and they're a little bit reticent, particularly when there's no strong evidence of, you know, clinically significant functional difference." (K3.Surgeon)<br><br>""You look at joint registry and, then increasingly especially the last joint registry, the interesting findings about resurfacing seems to be a good thing, although, it is implant dependent which goes beyond the KAT trial. So, people tend to stick with what they've stuck with, and I don't think many people have changed." (K2.Other-Coding expert/surgeon)<br><br>"There's two arguments they'll [surgeons who don't resurface] probably use, one is that if isn't done properly you get a fracture or whatever then actually those basis won't do as well. The other argument they will use is that actually usually if it's a patellofemoral problems, resurfacing the patella won't sort it because it's usually something else that's the problem like femoral rotation is the most common thing. So if you've not got that right, actually you shouldn't just be resurfacing the patella, you should be changing the whole knee." (K2.Coding expert/surgeon)<br><br>""Some people [surgeons] will do it [resurface] selectively, there's a small handful who do it selectively, there's good work going way back saying that was probably the worst of all the options is looking at it and deciding whether to do it or not but there are a handful of people doing that." (K2.Coding expert/surgeon)<br><br>""Whether people [surgeons] change or not, I suspect once people become a consultant, they won't change unless there's a really good reason to change." (K2.Coding expert/surgeon)<br><br>"You have to balance the reason why some people don't want to use the patella resurfacing. And the reason for that is because number one, it's like an extra step in the operation which makes the operation longer. Number two, you can mess it up. And if you mess it up, it's an absolute disaster. So, if you fracture the patella or they have some problem with it, yeah, actually they will do worse off than if you'd left it on their own." (K3.Surgeon)<br><br>""Some surgeons have come to the conclusion that actually it doesn't seem to make any difference if you leave it, whereas other surgeons such as our unit, just go, well, actually let's just do it, and if we can do it, let's just do it every time, do it well, and if you do it well and do it every time, then you're unlikely to have complications or problems." (K3.Surgeon)<br><br>""I think the that, you know, it's just a hugely, sort of, spiky topic when there's not really good evidence one way or the other. And really, the only evidence that you should do it is because if you don't do it, then there's a higher rate of doing it second time around. So, it's not actually that we know that they do that much better if you do it. It's just a defensive type of situation." (K3.Surgeon)<br><br>""They're [American surgeons are] slightly more defensive medical sort of fraternity probably from a joint replacement point of view. So, they feel as though, well, actually, you know, we've got to do it, because if you don't do it, you know, they might sue us for not doing it. And there's also a situation in America, which is slightly different from the UK, where you might have a unit down the road who are actually doing second operations on your cases because everyone's paid to do cases. You know, if you're paid to do cases, don't be surprised if you go see a surgeon and end up with an operation, whereas, the National Health Service is slightly different in that we're not paid to do cases so we try, and you could probably say a little bit more, there's a bit more, sort of, you know, objectivity to it, rather than just you know, doing cases because something's come and you're not being paid for it, so it's multifactorial. You know, we defend our results." (K4.Surgeon)<br><br>""I think, you know, surgeons and doctors have been left to their own devices to come up with a conclusion." (K3.Surgeon)<br><br>"Part of the problem is surgeons like using new shiny things, they must first use the new shiny thing which actually hasn't got any evidence for it, compared to the, you know, the, you know, the rusty old one but we actually know works well. So, because surgeons think they're cleverer than that, but they're not." (K3.Surgeon)	X	√	"[Participant:] You start as a GP, you might start to see people with later complications from the surg[illegible] when you see surgery that's happened many years ago and you start, if you've been the person's ge[illegible] practitioner for a long time, you'll see them coming back. And you'll see that, actually, a lot of these [illegible] varicose veins might be recurring. So, you actually as a GP will pick up the long complications that t[illegible] studies won't pick up at all. So.<br>[Researcher:] Yeah, 20 years would be a hard longitudinal study.<br>[Participant:] Yeah, it's really, it would be a really hard thing to research that. It would be a really big [illegible] over years and years and years and years. Very difficult to get funding for and get a study done. So [illegible] will be informed by anecdote and by personal experience in that area." (General3.General practitione[illegible]<br><br>"So, the vascular surgeons don't keep following up people with varicose vein surgery ever, I don't thi[illegible] (Laughter) They deal with them and they discharge them. That's it. Bye-bye. Off you go." (Genera[illegible] practitioner)<br><br>"There will be individual surgeon preferences; there'll be antidotal experiences from certain treatmen[illegible] there's always a push back we find in secondary care of specific treatments, that an individual favou[illegible] (RandCVV6.General practitioner)<br><br>"I don't think many clinicians take much notice of guidelines from NICE because that's not where the[illegible] education, really. You know, the studies that NICE has looked at are all things that the clinicians will [illegible] known about because they would have seen them presented at scientific meetings and so on anywa[illegible] (RVV1.Author)<br><br>"Clinicians aren't in general interested in cost-effectiveness. They're interested in clinical effectivenes[illegible] you're faced with a patient, you want to use the most effective treatment on them, not the most cost-[illegible] treatment." (RVV1.Author)<br><br>"There are all sorts of things that govern surgeons' decisions. They like new technologies. Most of [illegible] playing with new toys. So, if some new technology comes out that will do something more efficiently [illegible] good." (RVV1.Author)
**4.B. Self-efficacy** - *Individual belief in their own capabilities to* [illegible]	"The timing in which an NG tube or a PEG tube is made I think will also very much depend on whether that patient is being cared for on a stroke specialist ward or on a general medical ward. And I think generally if they're under the stroke team, then an NG tube tends to be placed quicker [illegible]		√	""I guess what I see my role in is being that halfway house between the definitive surgical and the procedure on the surgeon and nothing, and I can advise what best to do." (General4.General practitioner)	X	√	"One of the things is that UK EVAR trial 1 was one of the very early ones that started recruitment in 1998, 1999, when the expertise was less planned, it was not good, understanding of the importance of instructions for us and how to get the most out of [illegible]	X	√	"You might as well do it [resurface], because actually doing it second time around it doesn't work and it's more expensive. So, surgeons don't like that type of pressure." (K4.Surgeon)	X	√	"What we've seen is that it's also a societal and a change with both societal attitude and medical [illegible] think in that, actually, from going to varicose veins, one of the staples of general surgeons of [illegible]

BMJ Publishing Group Limited (BMJ) disclaims all liability and responsibility arising from any reliance placed on this supplemental material which has been supplied by the author(s)

				"I think we're [the surgeons are] sort of dependent on our referral pathways which often will come either through gastroenterology or direct from GPs. And then once they are referred to us, normally that's people that are already a bit or at least partially aware of what anti reflux surgery involves. And a lot of the patients we see if they're diagnosed with pathological reflux we'll proceed with surgery in general." (Ref1.Surgeon)									
**4.C. Individual Stage of Change** – *Characterization of the phase an individual is in, as he or she progresses toward skilled, enthusiastic, and sustained use of the intervention.*										""I suspect once people become a consultant, they won't change unless there's a really good reason to change." (K2.Coding expert/surgeon)	X	√	
**4.D. Individual Identification with Organization** – *A broad construct related to how individuals perceive the organization, and their relationship and degree of commitment with that organization.*	"[compared to NICE guidelines] there's more ownership by the clinicians of the RCP guidelines, I guess, it's more clear. You know, they come out at regular intervals - not very regular intervals. 2016 was the last, but they're working on a new set soon, I think. NICE comes out in dribs and drabs." (F1.Author)		√										
**4.E. Other Personal Attributes** – *A broad construct to include other personal traits such as tolerance of ambiguity, intellectual ability, motivation, values, competence, capacity, and learning style.*													
**5. Process – These constructs have been interpreted to include facilitators and barriers to implementation**													
**5.A. Planning** – *The degree to which a scheme or method of behavior and tasks for implementing an intervention are developed in advance, and the quality of those schemes or methods.*										"One of the hardest things to do when it comes to any type of departments tend to find is standardisation. So, we had an experience, you know, within orthopaedic departments within our area where they'd want to use a different type of hip replacement treatment. And we've got everybody together. And you know, they've had to come up with one hip replacement so it's gonna suit everybody and that's difficult. But keeping everyone in the room and keeping focused on that, we have been able to achieve that type of result. So, it's the same type of thing here. There will be individual surgeon preferences; there'll be antidotal experiences from certain treatments. And there's always a push back we find in secondary care of specific treatments, that...an individual favour. And what we found is that these types of processes have enabled directorates to standardise within their consult colleagues as well. And have a way of, you know, holding their colleagues, the directorates to account as well, for the choice of the procedure. And I think that helps within standardisation. Without this type of framework, it could be, you know, an unpoliced free for all, you know, whims and preferences." (RandCVV6.Other-General practitioner)		√	
**5.B. Engaging** - *Attracting and involving appropriate individuals in the implementation and use of the intervention through a combined strategy of social marketing, education, role modeling, training, and other similar activities.*													
**5.B1. Opinion Leaders** – *Individuals in an organization who have formal or informal influence on the attitudes and beliefs of their colleagues with respect to implementing the intervention.*							""Soon after that [the EVAR 15-year outcomes were published], several, a few months after, that came a consultation document of NICE guidelines would try to enforce that [the results of the 15-year follow up] into or put that, incorporate that, into clinical guidelines which created quite a lot of controversy and physicians got extremely excided about it. And that resulted in a lot of debate and representations to the NICE saying that that recommendation is actually it is not possible to implement it for various different reasons to the extent that one of the rare things to happen was the NICE have actually amended the guidelines to the extent that practically they lost their teeth." (E2.Surgeon)<br><br>"Over the years, I've gone to many, many, many vascular surgical meetings and it was always about the EVAR and always about how you could improve EVAR, and I never once heard anyone talk about open surgery and how I've learnt to do something differently that improves my outcomes. And it's almost like you were a dinosaur if you were talking about open surgery rather than the latest gizmo, so I think there's a huge amount of psychology and finance that is driven these manufacturers want to sell and physicians don't want to look like dinosaurs." (E3.Other-Radiologist)<br><br>"When EVAR first came in, I promoted it, I was- I went into vascular surgery to do open aortic surgery, I loved it, the idea of EVAR means taking away the bit of my practice I really most valued but I saw it as a really good thing and I was pushing it but a lot of my younger colleagues were not, but around all of it I knew jolly well if I had an aneurism, I want an EVAR, I had absolutely no doubt that I would- I can go to the complications I didn't want but I had no doubt at all." (E1.Surgeon)	X	√				"What happened about 2009, 2010, people were very short of money. And throughout a lot of the co[illegible] commissioners suddenly started restricting varicose vein treatments. For example, the patients who[illegible] actually developed an ulcer or actually had a bleed from their varicose veins. And bleeding is rare b[illegible] And, I mean, this was horrific because, obviously, what you want is if someone started to develop sk[illegible] you don't want them to get worse and worse and get an ulcer. And so, for example in my own area, [illegible] redated], I was involved in a group where the commissioners said, ""Look, we want to side-line varic[illegible] treatments, and we want to not treat anyone unless they bled or had an ulcer." We, the surgeons, a[illegible] agent abetted by the GP said, "This is crazy. You have to treat anybody who has got any sign their s[illegible] damaged because they're immediately showing the sign that they could get much worse and cost th[illegible] service a lot of money." And so, we, in this locality, produced guidelines which still exist." (RandVC[illegible]<br><br>"I would influence NICE and suggested they produce a guideline, which they did in 2013, suggesting[illegible] actually, the recommendation was that anyone with troublesome symptoms should be referred, and [illegible] obviously with skin damage and bleeding and things certainly should be referred urgently. And so, t[illegible] most, to me, the most powerful influence for the NHS about what they ought to be doing. And that N[illegible] guideline was produced in 2013 largely in response to what had happened in terms of the increasing[illegible] in treatment during the prior three or four years." (RandCVV2.Author)
**5.B2. Appointed Internal Implementation Leaders** – *Individuals from within the organization who have been formally appointed with responsibility for implementing an intervention as coordinator, project manager, team leader, or other similar role.*													
**5.B3. Champions** – *"Individuals who dedicate themselves to supporting, marketing, and 'driving through' an [implementation]" [101] (p. 182), overcoming indifference or resistance that the intervention may provoke in an organization.*	"[researcher:] Were there any champions that specifically led you in your organization that we need to switch, and you need to use the nasogastric tubes sooner, faster, than we insert the PEGs.<br>[participant:] No, because as I said, I think I can't remember a time in this trust where we just put a PEG in without discussion very early<br>[researcher:] Where in contrast, you can remember antibiotic champions and hand washing champions, I'm sure.<br>[participant:] Yeah. And yeah, oral hygiene champions and all sorts of things." (F4.Speech and Language Therapy)	x											
**5.B4. External Change Agents** – *Individuals who are affiliated with an outside entity who formally influence or facilitate intervention decisions in a desirable direction. Including the influence of manufacturers here*							"I suppose, EVAR is associated with the device, devices associated with manufacturers, and manufacturers associated with large wallets, and in my experience in medicine over the years, when a procedure which is cheap doesn't have a funded champion then the expensive treatment always seems to win out, and I find that very sad." (E3.Radiologist)<br><br>"I think there's a huge cost, there's a huge profit motive there [for EVAR]. They [manufacturers/brands] make a lot of money out of the patients and I think that in supplying endovascular grafts and so forth. And I think that they have invested a huge amount of money in sponsoring meetings, sponsoring speakers, advertising, and I think that has influenced the adoption of endovascular techniques for treating aneurysms. It's a subtle process, but if you support the advocates of endovascular treatment, and you don't support the advocates of open treatment, then inevitably the advocates of endovascular treatment will be the ones standing at the speaker's making the message, it might be a message they believe in. So, I'm not suggesting they're bought, but I'm just suggesting that they're sponsored to promote that message. And I think that's had a big impact in terms of, of the response, for example, to the nice recommendations are on either, which were considerably watered down after a fairly sort of robust response from the pro endovascular lobby. So I think the industry has had a big impact in that respect. And I have concerns that it is in some organizations slanting, the aneurysm repair, more towards evolve than is justified, but I think it's a difficult process. We need industry, but at the same time, we need to be careful about the industry." (RandCVV5.Sgueon)<br><br>"I think that that transition from open surgery to endovascular treatment has been progress, and I'm comfortable with it. I don't think it's been driven excessively by industry, I think it's been driven by clinicians. And I think that, although industry, I'm sure has had a ride on it. I think, as a whole, it's been a good development. And I think that it's the way forward in terms of the treatment of endovascular surgery. (RandCVV5.Surgeon)	X		"From a from a monetary point of view, you have these studies that are often funded by implant companies, et cetera. So, there is sort of, there's an inherent bias towards trying to prove that one person's implant is better than another." (K4.Surgeon)		√	
**5.B5. Key Stakeholders** – *Individuals from within the organization that are directly impacted by the innovation, e.g., staff responsible for making referrals to a new program or using a new work process.*													

				to take a pill every day, surgery is the best option." (Ref1.Surgeon)			done. And at the end of the day standard endovascular repair is actually a relatively straightforward set piece operation which can be done fairly safely and patients tend to improve relatively quickly, so there is a tendency to do it." (E2.Surgeon)<br><br>"There's a big element of patient choice here and it's how the information is presented and, you know, keyhole versus horrendous incision, patients come with a position that keyhole is better and society is moving that way so whatever the result show, if clinician presents two options, patient seemed to always want the minimal invasive option." (E3.Other-Radiologist)<br><br>"I imagine patients who don't know the downside, they don't hear so much about the downside and they're given a choice between massive incision intensive care or great treatment on the day and lower 30-day mortality, and people are very short-sighted in their thinking. You know, it's jam today rather than jam in 10 years that they want. So, I think patient choice is a big part, and if patients are not informed and don't understand those long-term data, and maybe don't care about the long-term data but I think that's part of it." (E3.Other-Radiologist)<br><br>"To my understanding, the NICE Guideline say that patients have to be offered both choices and, you know, so it'll be common that a patient might be of an age for some who would say you know what, we think you're better served with an open repair but if the patient chooses endovascular repair then, you know, they're not dining entirely ala carte from the NHS. But, you know, if they express a preference for one thing over the other and it's a strong one despite information, then that will proceed." (E3.Radiologist)<br><br>""I was just wondering about, just get it back up, yeah, sort of the patient preference. And actually, you know, how you balance the short-term benefits and the long-term benefits. And I think that a lot of that is about how surgeons and surgical practice potentially has changed in terms of being more open and having more of a discussion with the patient, and more shared decision-making with patients as to what's going to be the right option for them." (General3.General practitioner)<br><br>""We've written into several of our policies that we will, yeah. So now there is a box that has to be ticked that the commissioner will pay for it is, that a shared decision, a high-quality shared decision-making conversations taken place about whether this is the right thing for this patient or not. So, we've now got several commissioning policies that says we will pay for this intervention if you've had that conversation to decide it's the right thing for a patient. But if you haven't, we won't." (General3.General practitioner)<br><br>""I think for the most part, patients preferred to be guided by the clinician actually. I think 95% of people usually say, "It's funny, you know, if you ever try and do informed consent by what's it called, shared decision making, that's what, that you can go through five minutes of your very best shared decision-making using charts, colourful diagrams and your best clear English and plain, you know, and then at the end of it you say, "So, what's your shared decision on this?" And they go, "I don't know doc, what do you think I should you do?" And then you think, what was the point of that? So, for the most part, I think patients prefer the clinician to tell them what's the best thing to have. We get the occasional example of emails to the CCG where patients say, "I want say radiotherapy" or whatever but something that's not commissioned, we have to say, well, I'll email back saying, "Sorry, that's not what the value-based commissioning policy says at the moment according to best evidence and value for money so you can't have it." Generally, folks want to do what their clinicians tells them is the best according to the evidence." (General1.General practitioner)			assume that they just go and get what they feel is the most appropriate thing for them. So, it's interesting. So, it's not even, I don't even know if it's really a part of the consent process for myself or any of my colleagues actually or anybody I know." (K4.Surgeon)<br><br>""So knee replacement surgery is generally a very successful operation. But you're right, there is this persistent 15 percent of people who are not satisfied with their surgery, which it seems to be universal for whether or not you have, you know, whatever implant you have, whatever way is done, you know, whoever does it, no one has proven to get rid of that service." (K4.Surgeon)<br><br>""I think most of the evidence would also suggest that there is no significant impact, particularly at the level of clinical importance, from patients, in terms of patient reported outcome measures and satisfaction rates." (K3.Surgeon)<br><br>""Patients are very, very different and some patients really don't need much rehab at all. And some people need horrendous amounts of rehab to get places and to get the same. And some people actually have no pain and some people have, you know, the worst pain ever and it's just it's a really difficult subjects and then patient satisfaction doesn't necessarily always equal with post op pain." (K4.Surgeon)<br><br>""The difference between resurfacing and not is relatively small between the difference between having a knee replacement and not. So, I would've thought they [the patients] don't get too concerned about that. I mean, to me, one of the biggest is that after knee replacement, a significant proportion [of patients], perhaps over 10%, are unhappy, 10 to 20% are dissatisfied. And most of that is with pain. And if people haven't had a patellar resurfacing, surgeons may feel the pain is from the kneecap and they do a resurfacing. Whereas if they'd had a resurfacing, they can't do any more surgery. So, one of the things behind this is the fact you haven't done something means you're more likely to be able to do something again." (K1.Author)			"From patient point of view, there's no difference in the amount of time you spend in hospital, becaus case procedure. And the advantage to the patient in the procedurally part of the event is they sell loc anaesthetic versus a general anaesthetic procedure for the vast majority of time, although I did, prob percent of my varicose vein, open surgeries under local anaesthetic, so it is possible to do that. But recovery time, therefore, is quicker, so you go home quicker. So that's the first bit they like. Second [patients] like is that you can return to work almost immediately. Certainly within a few days of the in as long as you walk a reasonable amount each day, and don't just sit in an office with your feet dang for 8 hours at a time. So, there was a quick pro quo for that, but the recovery time was quicker. (RandCVV4.Surgeon)<br><br>"Varicose vein surgery was quite happily done by people if they could actually get to do it and was re groups of people who'd had ulcers or healed ulcers in particular." (RandCVV4.Surgeon)<br><br>"I think patients very much supported the switch [away from surgery]. So, if you look at the data, pati recovery and quality of life and complications, serious complications are much lower in the endother and recovery is much quicker, sorry, in the endovenous not just endothermal, in the endovenous is n quicker, and patients vote with their feet. So, you know, patients were coming and requesting endov treatment above surgery." (RandCVV3.Surgeon)<br><br>"Patient-reported outcome measures are not great for varicose veins surgery. Patients always get as doors about it and they remember the bruising and the battering and the fact their legs were swollen and they get phlebitis and things, so they tend not to like it much. The more effective data on outcon recovery done through class to show that the patients recovered quicker and felt better quicker with endothermal endovenous stuff than they did with surgical intervention. And so the patients liked it be because it was quicker, return to work was quicker. And as a consequence, they reported less pain swelling and all those other things." (RandCVV4.Surgeon)
**5.C. Executing –** *Carrying out or accomplishing the implementation according to plan.*													
**5.D. Reflecting & Evaluating** – *Quantitative and qualitative feedback about the progress and quality of implementation accompanied with regular personal and team debriefing about progress and experience.*													
Other Codes													
Accuracy of HES codes							"I actually think the steep trajectory upwards is somewhat confounded by the introduction of codes, although codes were introduced I suspect coding tracks probably although they are very good might have had a learning curve as well. I think in reality it was less steeper than that but nevertheless the trend is exactly the same that it went up." (E2.Surgeon)			"You know there's still some issues with the National Joint Registry data, though initially when it started in 2003 recording data onto the NJR was not mandatory, and rates of compliance were quite poor in the first few years of the registry. Mandatory reporting only came online for the NJR I think about ten years ago, though some of the historic NJR data may not be fully representative of the clinical picture, but I think it's fair to say that, you know, I was very surprised looking at your initial graph that you had a rate of patella resurfacing of zero per cent to start with. That would strike me as a miscoding." (K3.Surgeon)<br><br>"I think the HES coding bit of it is plain straight down to how the codes were applied. And for this particular one, there was a change in the logic and I've emailed you the dates because, I can't remember them straight off hand, where several things happened. So you switch to creating HRGs out of the HES codes which were based on the basis for the payment by results. In amongst that then came the payment system that they bolted on to that. At some point and I think I've sent you the dates, they switched on the logic which meant that if you coded the patella resurfacing at the time of a knee replacement, you got paid an uplift of a couple of a thousand pounds I think it is." (K2.Coding expert/surgeon)<br><br>"I think the only way you're going to find out if patella resurfacing is done or not and fitting it into timescale as, I would probably not use that as data, I would use NJR data." (K2.Coding expert/surgeon)<br><br>"Normally it's [HES is] very good at looking at change over time except in instances like this where there is a huge confounding factor being chucked in around how coding guidance and payment was applied." (K2.Coding expert/surgeon)<br><br>"That chart you're showing there doesn't really, I think active...reflects probably proper practice and what's been happening because even back in the early 2000s patellas were being resurfaced to the much higher rate than zero percent, so I suspect there has been some inaccuracy with patella resurfacing." (K4.Surgeon)<br><br>"Coding changes are normally not driven by the surgeons anyway; they're driven by the managers who want the money." (General1.General practitioner)<br><br>"I suspect the reason when that jump happened, I bet you any money that they started to code on that they would get paid more money if they coded for patella resurfacing as well as total knee replacement." (General1.General practitioner)<br><br>"We've always been rather cautious about HES data. And I would say the best source of data, really, would be the National Joint Registry because they record details of every implant. And the patella resurfacing is an implant, so they would have that data. And, certainly, my perception was that, back in the early 2000s, more than nought percent of people were doing patellar resurfacings. So, I would certainly agree that the data isn't what one would've expected. And, you know, I would've thought it wouldn't have started it at nought, but maybe started, perhaps, at 30% and gone up to 60% over that time-period." (K1.Author)			

[1] Dennis M, Lewis S, Cranswick G, Forbes J. FOOD: a multicentre randomised trial evaluating feeding policies in patients admitted to hospital with a recent stroke. *Health Technol Assess* 2006;10(2):1–120. doi: 10.3310/hta10020

[2] Grant A, Wileman S, Ramsay C, Bojke L, Epstein D, Sculpher M, Macran S, Kilonzo M, Vale L, Francis J, Mowat A, Krukowski Z, Heading R, Thursz M, Russell I, Campbell M, REFLUX Trial Group. The effectiveness and cost-effectiveness of minimal access surgery amongst people with gastro-oesophageal reflux disease - a UK collaborative study. The REFLUX trial. *Health Technol Assess* 2008;12(31):1–181. doi: 10.3310/hta12310

[3] Grant A, Boachie C, Cotton S, Faria R, Bojke L. Clinical and economic evaluation of laparoscopic surgery compared with medical management for gastro-oesophageal reflux disease: 5-year follow-up of multicentre randomised trial (the REFLUX trial). *Health Technol Assess* 2013;17(22):1–167. doi: 10.3310/hta17220

[4] Brown L, Powell J, Thompson S, Epstein D, Sculpher M. The UK EndoVascular Aneurysm Repair (EVAR) trials: randomised trials of EVAR versus standard therapy. Health Technol Assess 2012;16(9):1–128. doi: 10.3310/hta16090

[5] Murray D, MacLennan G, Breeman S, Dakin H, Johnston L, Campbell M et al. A randomised controlled trial of the clinical effectiveness and cost-effectiveness of different knee prostheses: the Knee Arthroplasty Trial (KAT). *Health Technol Assess* 2014;18(19):1–235. doi: 10.3310/hta18190

[6] Michaels J, Campbell B, Brazier J, MacIntyre J, Palfreyman S. Ratcliffeand J, Rigby K. Randomised clinical trial, observational study and assessment of cost-effectiveness of the treatment of varicose veins (REACTIV trial). *Health Technol Assess* 2006;10(13):1–196. doi: 10.3310/hta10130

[7] Brittenden J, Cotton S, Elders A, Tassie E, Scotland G, Ramsay C, et al. Clinical and cost-effectiveness of foam sclerotherapy, endovenous laser ablation and surgery for varicose veins: results from the CLaSS trial. *Health Technol Assess* 2015;19(27). doi: 10.3310/hta19270
